# Mutant *C9orf72* human iPSC‐derived astrocytes cause non‐cell autonomous motor neuron pathophysiology

**DOI:** 10.1002/glia.23761

**Published:** 2019-12-16

**Authors:** Chen Zhao, Anna‐Claire Devlin, Amit K. Chouhan, Bhuvaneish T. Selvaraj, Maria Stavrou, Karen Burr, Veronica Brivio, Xin He, Arpan R. Mehta, David Story, Christopher E. Shaw, Owen Dando, Giles E. Hardingham, Gareth B. Miles, Siddharthan Chandran

**Affiliations:** ^1^ Euan MacDonald Centre for MND Research The University of Edinburgh Edinburgh UK; ^2^ Centre for Clinical Brain Sciences The University of Edinburgh Edinburgh UK; ^3^ School of Psychology and Neuroscience University of St Andrews St Andrews, Fife UK; ^4^ Dementia Research Institute at the University of Edinburgh Edinburgh UK; ^5^ Centre for Discovery Brain Sciences The University of Edinburgh Edinburgh UK; ^6^ MRC Centre for Neurodegeneration Research King's College London, Institute of Psychiatry London UK; ^7^ Dementia Research Institute at Kings College London, Maurice Wohl Clinical Neuroscience Institute London UK; ^8^ Centre for Brain Development and Repair Institute for Stem Cell Biology and Regenerative Medicine Bangalore India

**Keywords:** ALS, *C9orf72*, iPSCs, motor neuron, non‐cell autonomous

## Abstract

Mutations in *C9orf72* are the most common genetic cause of amyotrophic lateral sclerosis (ALS). Accumulating evidence implicates astrocytes as important non‐cell autonomous contributors to ALS pathogenesis, although the potential deleterious effects of astrocytes on the function of motor neurons remains to be determined in a completely humanized model of *C9orf72*‐mediated ALS. Here, we use a human iPSC‐based model to study the cell autonomous and non‐autonomous consequences of mutant *C9orf72* expression by astrocytes. We show that mutant astrocytes both recapitulate key aspects of *C9orf72*‐related ALS pathology and, upon co‐culture, cause motor neurons to undergo a progressive loss of action potential output due to decreases in the magnitude of voltage‐activated Na^+^ and K^+^ currents. Importantly, CRISPR/Cas‐9 mediated excision of the *C9orf72* repeat expansion reverses these phenotypes, confirming that the *C9orf72* mutation is responsible for both cell‐autonomous astrocyte pathology and non‐cell autonomous motor neuron pathophysiology.

## Main points

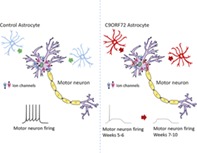

- Human iPSC‐derived astrocytes harboring *C9orf72* mutations recapitulate key aspects of ALS pathology and cause non‐cell autonomous pathophysiology in human iPSC‐derived motor neurons.
- The pathophysiology induced in motor neurons by ALS astrocytes is characterised by a progressive loss of action potential output due to a decrease in voltage‐gated sodium and potassium currents.
- CRISPR/Cas9 mediated excision of *C9orf72* repeat expansions reverses the pathophysiological effects of astrocytes on motor neurons.

## 1 INTRODUCTION

Although amyotrophic lateral sclerosis (ALS) is characterized by loss of motor neurons (MNs), accumulating experimental and pathological evidence reveal the involvement of other cell types that are implicated in non‐cell autonomous toxic effects on MN health (Boillee, Vande Velde, & Cleveland, 2006; Ilieva, Polymenidou, & Cleveland, 2009). Astrocyte pathology is prominent, with an emerging consensus, particularly from SOD1 based studies, that astrocytes appear critical to disease progression (Papadeas, Kraig, O'Banion, Lepore, & Maragakis, 2011; Wang, Gutmann, & Roos, 2011; Yamanaka et al., 2008). It has also been shown that astrocytes derived from sporadic or familial cases can, upon co‐culture or upon exposure to astrocyte conditioned media (ACM), be directly toxic to MNs leading to cell death (Cassina et al., 2008; Di Giorgio, Carrasco, Siao, Maniatis, & Eggan, 2007; Fritz et al., 2013; Haidet‐Phillips et al., 2011; Kia, McAvoy, Krishnamurthy, Trotti, & Pasinelli, 2018; Madill et al., 2017; Marchetto et al., 2008; Nagai et al., 2007; Phatnani et al., 2013; Re et al., 2014; Rojas, Cortes, Abarzua, Dyrda, & van Zundert, 2014). In contrast, the influence of astrocytes on MN function is unclear and comparatively understudied. Importantly, altered MN function, specifically perturbations in excitability that may initiate or contribute to a cascade of excitotoxic disease mechanisms, represent the earliest observed phenotype in animal models of ALS (Delestree et al., 2014; Kuo et al., 2004; Pieri et al., 2003; Quinlan, Schuster, Fu, Siddique, & Heckman, 2011; van Zundert et al., 2008). Changes in MN excitability have also been reported in ALS patients (Kanai et al., 2012; Vucic & Kiernan, 2006) and more recently in human induced pluripotent stem cell (iPSC)‐based models of ALS (Devlin et al., 2015; Naujock et al., 2016; Sareen et al., 2013; Wainger et al., 2014). Studies of MN dysfunction in ALS have primarily focused on cell autonomous mechanisms, whereas a recent study has shown that changes in the physiological properties of MNs can be mediated by ALS‐affected mouse astrocytes (Fritz et al., 2013). Together, these studies highlight the need to better understand the nature and functional consequences of astrocyte pathology in ALS.

The finding that sporadic ALS (sALS) is phenotypically and pathologically indistinguishable from familial ALS (fALS) (Ajroud‐Driss & Siddique, 2015; Freischmidt, Muller, Ludolph, Weishaupt, & Andersen, 2017; Sreedharan et al., 2008) highlights the value of studying fALS to determine common pathogenic pathways (Hardiman et al., 2017). The intronic hexanucleotide repeat expansion GGGGCC (G_4_C_2_) in the chromosome 9 open reading frame 72 (*C9orf72*) gene is the most common genetic cause of ALS, accounting for ~40% of fALS and ~10% of sALS (DeJesus‐Hernandez et al., 2011; Renton et al., 2011). In contrast to extensive studies focused on MN biology and pathology, the consequences of G_4_C_2_ expansion at physiological levels on astrocytes is understudied (Meyer et al., 2014). Moreover, the functional interaction between MNs and astrocytes is yet to be investigated in a completely humanized model of *C9orf72*‐mediated ALS.

We and others have previously shown that ALS patient‐derived iPSC lines recapitulate key aspects of ALS pathology and MN dysfunction (Bilican et al., 2012; Devlin et al., 2015; Donnelly et al., 2013; Serio et al., 2013; Shi et al., 2018). An important advance in human iPSC‐based disease modeling is the use of paired isogenic control lines which help establish causality between a given mutation and phenotypes (Sandoe & Eggan, 2013; Selvaraj, Livesey, & Chandran, 2017; Wang et al., 2017). Using CRISPR/Cas9‐mediated genome editing to selectively excise G_4_C_2_ repeats we have recently shown selective human MN vulnerability to AMPA receptor mediated excitotoxicity that is mutation dependent (Selvaraj et al., 2018). In this study, we have utilized this system to further explore the cell‐autonomous and non‐cell autonomous consequences of *C9orf72* mutation on iPSC‐derived astrocytes and MNs. We report *C9orf72*‐dependent cell autonomous astrocyte pathology and astrocyte mediated loss of MN function independent of overt effects on MN viability. Furthermore, we suggest possible molecular pathways, highlighted from RNA‐Seq data, which may underlie loss of MN function.

## 2 MATERIALS & METHODS

### 2.1 Generation of iPSC lines and isogenic control iPSC lines

Eight iPSC lines were used in this study, including two healthy controls (Con‐1 and Con‐2), three ALS patient lines carrying the G_4_C_2_ repeat expansion in the *C9orf72 gene* (C9‐1, C9‐2 and C9‐3) and 3 isogenic control lines C9‐1 (C9‐Δ1), C9‐2 (C9‐Δ2), and C9‐3 (C9‐Δ3). IPSCs were generated from fibroblasts using either Sendai virus or retrovirus expressing the Yamanaka transcription factors (OCT3/4, SOX2, c‐Myc, and KLF4) (Bilican et al., 2012; Devlin et al., 2015; Selvaraj et al., 2018; Takahashi et al., 2007). These were conducted under full Ethical/Institutional Review Board approval at the University of Edinburgh (Con‐1 and Con‐2), at University College London (C9‐1) and at King's College of London (C9‐2 and C9‐3). The isogenic control lines of C9‐1, C9‐2, and C9‐3, named C9‐Δ1, C9‐Δ2, and C9‐Δ3 respectively, were generated by using CRISPR/Cas9‐mediated genome editing to selectively excise the G_4_C_2_ repeat expansion as described in the following section (Ran et al., 2013; Selvaraj et al., 2018).

### 2.2 CRISPR/Cas9 gene correction

The C9‐Δ1, C9‐Δ2, and C9‐Δ3, were generated from their respective parental patient line C9‐1, C9‐2, and C9‐3 which have multiple G_4_C_2_ repeats in the wild‐type allele and in the mutant allele (Selvaraj et al., 2018). Two guide RNAs (gRNA‐1:5′GACTCAGGAGTCGCGCGCTA‐3′ and gRNA‐2: GGCCCGCCCCGACCACGCCC) flanking the G_4_C_2_ repeat expansion were cloned into the pSpCas9 (BB)‐2A‐GFP vector. C9ORF72 patient iPSCs were transfected with these vectors to induce double strand break in the DNA sequence at a precise locus resulting in the deletion of G_4_C_2_ repeats. Individual iPSC clones were screened for deletion of G_4_C_2_ using the repeat‐primed PCR. One positive clone for each isogenic control line C9‐Δ1, C9‐Δ2, and C9‐Δ3 was selected, and Sanger sequencing for the *C9orf72* G_4_C_2_ locus in these clone demonstrated complete deletion of G_4_C_2_ repeats in the mutant allele and one remaining G_4_C_2_ in the wild‐type allele (Selvaraj et al., 2018).

### 2.3 Generation of MNs from iPSCs

Differentiation of iPSCs into a neuronal and MN lineage was performed using minor modifications of previously established and validated protocols (Amoroso et al., 2013; Bilican et al., 2012; Devlin et al., 2015). The iPSCs were neutralized to neuroectoderm using dual SMAD inhibition in Phase I medium for 4–10 days. Neurospheres were patterned to a caudal, spinal cord identity in Phase II medium for 4–10 days. Caudalized neural stem cells (NSCs) were next ventralized in Phase III medium for 4–10 days, and then cultured in Phase III‐FGF for another 4–14 days. Caudalized and ventralized NSCs were transitioned to MN maturation medium for 2–6 weeks. These MN spheres were dissociated into single cells which were plated onto monolayers of astrocytes for co‐culture as described below. Complete media changes were conducted every 2–3 days during the MN generation process. Components of the media used are as follows. CDM contains 50% Iscove's Modified Dulbecco's Medium (IMDM) (Invitrogen), 50% F12, 5 mg/ml bovine serum albumin (BSA, Europa), 1% chemically defined Lipid 100x (Invitrogen), 450 mM Monothioglycerol (Sigma), 7 mg/ml Insulin (Roche), 15 mg/ml Transferrin (Roche), 1% penicillin/streptomycin (Invitrogen). Phase I medium contains CDM, 1 mM N‐ acetyl cysteine (Sigma), 10 μM Activin Inhibitor (R&D Systems) and 100 μM LDN (Merck Millipore). Phase II medium contains CDM, 1 mM N‐acetyl cysteine, 5 ng/ml fibroblast growth factor (FGF; PeproTech)/heparin (Sigma; 20 μg/ml), and 0.1 μM retinoic acid (Sigma). Phase III medium contains Advanced Dulbecco's Modified Eagle Medium (DMEM) Nutrient Mixture F12 (Invitrogen), 1% penicillin/streptomycin, 0.5% GlutaMAX, 1% B‐27, 0.5% N‐2 supplement, 5 ng/ml FGF + Heparin, 1 μM retinoic acid, and 1 μM purmorphamine (Merck Millipore). Phase III‐FGF contains Advanced Dulbecco's Modified Eagle Medium (DMEM) Nutrient Mixture F12 (Invitrogen), 1% penicillin/streptomycin, 0.5% GlutaMAX, 1% B‐27, 0.5% N‐2 supplement, 1 μM retinoic acid 0.5 μM purmorphamine (Merck Millipore). MN maturation medium contains advanced DMEM/F12, 1% penicillin/streptomycin, 0.5% B‐27, 0.5% N‐2 supplement, 2 ng/ml Heparin, 10 ng/ml brain‐derived neurotrophic factor (BDNF; R&D systems), 10 ng/ml glial cell line‐derived neurotrophic factor (GDNF; R&D systems), 10 μM forskolin (R&D systems), 0.1 μM retinoic acid, and 0.1 μM purmorphamine. At least 4 iPSC differentiations were performed for each experiment.

### 2.4 Generation of astrocytes from iPSCs

IPSCs were neutralized and then converted to spheres as described in the previous section (Bilican et al., 2012; Serio et al., 2013). Next, spheres were cultured in MN maturation medium for 2–4 weeks before being chopped and cultured in NSCR EL20 medium for 4–6 weeks to induce astrogliogenesis. At the end of this conversion phase, medium was switched to NSCR EF20 medium to maintain the proliferation of astrocyte progenitor cells (APCs) in spheres. These astrospheres were dissociated into single cells using the Papain Dissociation System (Worthington Biochemical) and plated onto 6‐well Matrigel (BD Biosciences, 1:80 diluted) coated plates at a density of 7.5 × 10^5^ to obtain monolayers of APCs, which were subsequently differentiated into astrocytes by switching the medium to AstroMed CNTF medium for 2 weeks. All media were changed every 2–3 days during the astrocyte generation process. NSCR EF20 contains Advanced DMEM/F12 (Invitrogen), 1% N2 supplement (Invitrogen), 1% B‐27 supplement (Invitrogen), 1% penicillin/streptomycin (Invitrogen), 1% GlutaMAX solution (Invitrogen), 20 ng/ml fibroblast‐growth factor 2 (FGF‐2; PeproTech), and 20 ng/ml epidermal growth factor (EGF; R&D Systems). AstroMED CNTF contains Neurobasal medium (Invitrogen), 2% B‐27 supplement (Invitrogen), 1% NEAA (Invitrogen), 1% penicillin/streptomycin (Invitrogen), 1% GlutaMAX (Invitrogen), and 10 ng/ml ciliary neurotrophic factor (CNTF; R&D Systems). At least 4 iPSC differentiations were performed for each line with control and patient iPSC differentiations always run in parallel. For co‐culture with enriched populations of MNs, astrocyte media was modified to contain 0.1% B‐27 supplement in NSCR EF‐20 and to 0.2% in AstroMED CNTF.

### 2.5 Generation of enriched MN cultures from iPSCs

Differentiation of iPSCs into a neuronal and MN lineage was performed using a modification of a previously published protocol (Maury et al., 2015) which allows for generation of an enriched culture with approximately 50–60% MNs, as evidenced by Isl‐1/2^+^ immunostaining within 14–16 days (Selvaraj et al., 2018) and minimal labeling for the astrocytic marker GFAP (Selvaraj et al., 2018, Figure S2). Neurospheres were dissociated, plated at a density of 30–40,000 cells per well in 24‐well plates (Fisher Scientific) on 13 mm glass coverslips (VWR) treated overnight with poly‐ornithine (0.01%, Sigma‐Aldrich), and further coated with Matrigel (1 in 10 dilution, VWR), fibronectin (10 μg/ml, Sigma‐Aldrich), and laminin (5 μg/ml, Sigma‐Aldrich). Plate down medium consisted of Neurobasal medium, 1% penicillin/streptomycin, 1% GlutaMAX, 1% NEAA, 1% B‐27, 1% N‐2, 2‐Mercaptoethanol (BME, 0.1 mM, Thermo Fisher Scientific), 10 ng/ml GDNF, 10 ng/ml BDNF, 10 ng/ml CNTF, 10 ng/ml IGF‐1(R&D Systems), 1 μM retinoic acid, 2.27 μM ascorbic acid (Sigma), 25 μM l‐glutamic acid (Sigma), and 1 μM Uridine/ 5‐Fluoro‐2′‐deoxyuridine U/FDU (Sigma). Twelve hours post‐plating, fresh media without addition of L‐Glutamic acid was added to cells. From this point onwards media, which contained no l‐glutamic acid but contained U/FDU, was changed on alternate days.

### 2.6 Generation of cortical neuron cultures from human stem cells

A complete and systematic description of the derivation of human cortical neurons from human stem cells can be found in Bilican et al. (Bilican et al., 2014). Briefly, human cortical neurons were differentiated from anterior neural precursors, which were derived from the H9 human embryonic stem cell line (WiCell), obtained under ethical/IRB approval of the University of Edinburgh. Experiments were carried out on human cortical neurons that had been differentiated and maintained in culture for at least 30 days in vitro (DIV). At these time points, around 70% of cells were neuronal (β3‐tubulin+), with little contamination from neural precursor cells (nestin+), astrocytes (GFAP+) or GABAergic (GAD65/67+) interneurons (Bilican et al., 2014; Livesey, Magnani, Hardingham, Chandran, & Wyllie, 2015). Neurons exhibited markers (VGLUT1+) consistent with an excitatory identity and also exhibited properties of neurones of the upper and lower layers of the cortex (Bilican et al., 2014; Livesey et al., 2015).

### 2.7 Immunofluorescence

Cells were fixed in 4% (wt/vol) paraformaldehyde for 10 min, permeabilized with 0.2% Triton X‐100 for 5 min and blocked in 3% (vol/vol) goat serum (Dako) or donkey serum (Sigma) for 45 min. They were then incubated in primary antibodies for 45 min followed by secondary antibodies for 30 min (Alexa Fluor dyes, 1:1000, Invitrogen). All antibodies were diluted in the blocking buffer. Nuclei were counterstained with DAPI (Sigma) for 5 min and coverslips were mounted on slides with FluorSave (Merck). All procedures were performed at room temperature. Primary antibodies used in this study were Vimentin (1:100, Millipore), NFIA (1:250, abcam), GFAP (1:500, Dako), GFAP (1:500, Sigma), S100B (1:500, Dako), βIII‐tubulin (1:1000, Sigma), TDP‐43 (1:250, Abnova), NANOG (1:250, R&D Systems), SOX2 (1:250, Millipore), TRA‐1‐60 (1:250, Santa Cruz), OCT3/4 (1:250, Santa Cruz), SOX1 (1:100, R&D Systems), Nestin (1:1000, Millipore), Brachyury (1:100, R&D Systems), EOMES (1:600, abcam), FOXA2 (1:100, R&D Systems), GATA‐4 (1:100, Santa Cruz), SMI32 (1:250, Covance), and Caspase‐3 (1:500, Abcam).

Fluorescent imaging was performed on fields of view containing uniform DAPI staining using either an Axio Observer.Z1 (Zeiss) epifluorescence microscope or an LSM710 confocal microscope (Carl Zeiss). Images were processed and blindly analyzed by using the ImageJ64 (v 1.47) software.

### 2.8 Glutamate uptake assay

2‐week old differentiated astrocytes were dissociated into single cells using Accutase and plated on 96‐well plates coated with Matrigel (1:80 diluted) at a density of 2.5x10^4^ cells per well in AstroMedCNTF medium. Five days later, astrocytes were exposed to 100 μM L‐glutamic acid (ATT Bioquest), and supernatants were collected at 30′, 60′, and 120′, respectively. A negative control was also setup by exposing astrocytes to 100 μM l‐glutamic acid supplemented with 2 mM l‐trans‐Pyrrolidine‐2,4‐dicarboxylic acid (PDC, Sigma) for 120′. Amplite™ Fluorimetric Glutamic Acid Assay Kit (ATT Bioquest) was used to determine the residual concentrations of l‐glutamic acid in supernatants following the manufacturer's instructions. The glutamate uptake was calculated by subtracting the remaining concentration from 100 μM. The cell number in each well was determined by using a CyQUANT® NF Cell Proliferation Assay Kit (Life Technologies, C35006) following the manufacturer's instructions. The glutamate uptake was normalized to the cell number and presented as uptake concentration per 1,000 cells.

### 2.9 Calcium imaging

2‐week old differentiated astrocytes were dissociated into single cells using Accutase and plated on μ‐Slide 8 Well Glass Bottom (ibidi) chambers coated with Matrigel (1:80 diluted) at a density of 1.5 × 10^5^ cells per well. Fourdays later, astrocytes were loaded with fluo‐4 acetoxymethyl ester (Fluor‐4 AM) (Life Technologies) diluted in Neurobasal® Medium (Invitrogen) for 1 hour at 37°C. Astrocytes were then washed for three times and left in Neurobasal® Medium for 30 min at 37°C. A negative control was set up by applying 50 μM 2‐APB (Calbiochem), an inhibitor of the IP3‐dependent calcium release, at this stage. The medium was switched to Dulbecco's Phosphate‐Buffered Saline (Life Technologies) prior to imaging. Glass beads (200 μm diameter) were dropped on top of astrocyte cultures as a mechanical stimulus. Time‐lapse imaging was performed using an Axio Observer.Z1 (Carl Zeiss) epifluorescence microscope at 10× magnification with a 488 nm excitation filter at 37°C and 5% CO_2_.

### 2.10 qRT‐PCR

Total RNA was isolated from 2‐week old differentiated astrocytes using an RNeasy Mini Kit (Qiagen) following the manufacturer's instructions. Five hundred nanograms of RNA was reverse transcribed to complementary DNA (cDNA) using a DyNAmocDNA Synthesis Kit (Thermo Scientific) following manufacturers' instructions. RT‐PCR reactions were performed in triplicate using a DyNAmo™ ColorFlash SYBR® Green qPCR Kit (Thermo Scientific, F‐416) following the manufacturers' instructions, and a C1000™ Thermal Cycler with a CFX96 Real‐time System (Bio‐Rad) was used to conduct the cycling. Primer sequences (5′➔3′) are C9orf72 total F TGTGACAGTTGGAATGCAGTGA, C9orf72 total R GCCACTTAAAGCAATCTCTGTCTTG, Beta‐Actin F GTTACAGGAAGTCCCTTGCCATCC, and Beta‐Actin R CACCTCCCCTGTGTGGACTTGGG. The CFX Manager™ Software (Bio‐Rad) with the 2^‐ΔΔCt^ method was used to calculate relative gene expression levels.

### 2.11 Western blot

2‐week old differentiated astrocytes were lysed in cold radioimmunoprecipitation assay (RIPA) buffer (50 mM Trizma® base, 150 mM NaCl, 1% TritonX‐100, 0.5% sodium deoxycholate, 0.1% SDS, and 2 mM EDTA, all from Sigma) supplemented with 1× protease inhibitor (Roche) and 1× phosphatase inhibitor (Roche), and incubated for 30 min on ice. Lysate was then centrifuged at 13000 rpm for 30 min at 4°C, and the resulting supernatant was collected as the RIPA‐soluble fraction. RIPA‐insoluble pellets were further washed with RIPA buffer once and then dissolved in Urea buffer (7 M Urea, 2 M Thiourea, 4% CHAPS, and 30 mM Trizma® base, all from Sigma) supplemented with 1× protease inhibitor and 1× phosphatase inhibitor of a volume in proportion to the soluble fraction. Sonication was performed to further dissolve the protein. After centrifugation at 13000 rpm for 30 min at 4°C, the supernatant was collected as the RIPA‐insoluble fraction.

Protein concentrations in the RIPA‐soluble fraction were determined using a Pierce™ BCA Protein Assay Kit (Thermo Scientific) following the manufacturer's instructions, and 10 μg of protein of each sample was loaded in an 8–20% Precise™ Protein Gel (Thermo Scientific) for SDS‐PAGE. The amount of insoluble protein was adjusted based on Coomassie Brilliant Blue staining on a duplicate gel to ensure equal loading across samples. Separated proteins were transferred to an Immobilon‐FL PVDF membrane (Millipore) and blocked in Odyssey Blocking Buffer (LI‐COR) for an hour at room temperature. Primary antibodies diluted in Odyssey Blocking Buffer (LI‐COR) were applied at 4°C overnight. After three washes with 0.1% PBS‐Tween, IRDye® Secondary Antibodies (LI‐COR) (1:15000 diluted in Odyssey Blocking Buffer) were applied for 1 hour at room temperature followed by another 3 washes with 0.1% PBS‐Tween. Membranes were imaged using an Odyssey® Fc Imager (LI‐COR), and images were processed and analyzed using the Image Studio™ software (LI‐COR). Primary antibodies used in this study were TDP‐43 (Proteintech 1:2000), GAPDH (Calbiochem, 1:15000), C9ORF72 (Santa Cruz, 1:2000), and poly‐GP (Proteintech, 1:2000).

### 2.12 RNA‐fish

Astrocytes on glass coverslips were fixed with 4% paraformaldehyde (Agar Scientific) for 15 min at room temperature followed by permeabilization in 70% ethanol at 4°C overnight. Cells were then re‐hydrated in 50% formamide (Sigma)/2x SSC (Sigma) for 10 min at room temperature and blocked in hybridization buffer (50% Formamide (Sigma), 2×SSC (Sigma), 10% Dextran Sulfate (Millipore), 1 mg/ml Yeast tRNA (Invitrogen) and 1 mg/ml Salmon Sperm DNA (Invitrogen)) for 30 min at 45°C. 50 ng of an Alexa Fluor® 546‐conjugated (GGCCCC)_4_ probe (IDT) diluted in the hybridization buffer was applied on cells for 2 hours at 45°C in a humidified chamber. After the hybridization, cells were washed twice with 50% formamide/2× SSC for 30 min at 45°C and then once with 2× SSC for 30 min at room temperature. After another three washes with PBS at room temperature, immunofluorescence imaging was performed as described previously.

As controls, cells were treated with either 3 U/ml DNase (Life Technologies) or 100 μg/ml RNase (Sigma) diluted in 2x SSC for 1 hour at 37°C prior to the hybridization step. In addition, an anti‐sense RNA probe against the CCTG repeat expansion was also applied on cells to assess the specificity of the (GGCCCC)_4_ probe.

### 2.13 LDH assay

Two‐week old differentiated astrocytes were dissociated into single cells using Accutase and plated on 96‐well plates coated with Matrigel (1:80 diluted) at a density of 2.5 × 10^4^ cells per well. Cells were washed once with Neurobasal® Medium prior to replacement with fresh AstroMed CNTF medium. Twenty‐four hours later, conditioned medium was collected and the concentration of lactate dehydrogenase (LDH) was measured using a CytoTox‐ONE™ Homogeneous Membrane Integrity Assay (Promega) following the manufacturer's instructions.

### 2.14 Population viability assay

APCs were plated at a density of 1.5x10^5^ cells per well in 24‐well plates on plastic coverslips (Thermo Scientific) coated with laminin (Sigma), fibronectin (Sigma), and Matrigel (BD Biosciences) in NSCR EF20 medium for 5–7 days followed by differentiation into astrocytes for a further 2 weeks in AstroMED CNTF medium. Control iPSC‐derived MN progenitors were dissociated with the Papain Dissociation System (Worthington Biochemical) and plated at a density of 5 × 10^4^ cells per well on top of the astrocytes once the astrocyte medium had been removed. MN plate down medium consisted of Neurobasal medium, 1% penicillin/streptomycin, 0.5% GlutaMAX, 0.5% B‐27, 0.5% N‐2 Supplement, 20 ng/ml basic FGF, 1 μM retinoic acid, 1 μM purmorphamine, 1 μM mouse Smo agonist SAG (Merck Millipore). Twenty‐four hours post MN plating, 20 ng/ml CNTF; R&D, 10 ng/ml GDNF, and 10 μM forskolin were added, with this medium used until day 14, feeding every 3 days. From day 14, RA, SAG, purmorphamine and forskolin was removed from the medium, with cells then maintained for up to 10 weeks.

Population cell viability of control iPSC‐derived MNs was performed with the observer blinded to the cell lines by counting the number of MNs on astrocytes stained with the MN marker SMI‐32, the apoptotic marker caspase‐3 and the nuclear marker DAPI. Twenty images were taken from each line at weeks 5–6 and 7–10 post‐plating.

### 2.15 RNA‐Seq

Total RNA from mature, iPSC‐derived astrocytes was assessed for quality (Agilent Bionalyzer) and quantity (Invitrogen Qubit) before library preparation. Illumina libraries were prepared from 1 μg of total RNA using TruSeq RNA Sample Prep Kit v2 with a 10 cycle enrichment step as per the manufacturer's recommendations. Final libraries were pooled in equimolar proportions before Illumina sequencing on a HiSeq 2500 platform using 100 base paired‐end reads. Reads were mapped to the primary assembly of the human (hg38) reference genome contained in Ensembl release 90 (Zerbino et al., 2018). Alignment was performed with STAR, version 2.5.3a (Dobin et al., 2013). Tables of per‐gene read counts were generated from the mapped reads with featureCounts v1.5.2 (Liao, Smyth, & Shi, 2014). Differential expression analysis was then performed using DESeq2 (R package version 1.18.1) (Love, Huber, & Anders, 2014). Gene ontology enrichment analysis was performed using topGO (R package version 2.30.1) (Alexa, Rahnenfuhrer, & Lengauer, 2006).

### 2.16 Electrophysiology

Whole‐cell patch‐clamp recordings were used to assess the functionality of iPSC‐derived MNs. Voltage‐clamp mode was used to investigate intrinsic membrane properties. Current‐clamp mode was used to investigate the firing properties of MNs. Experiments were carried out in a recording chamber which was perfused continuously with oxygenated artificial cerebral spinal fluid (aCSF) at room temperature (22–24°C). Whole‐cell patch‐clamp recordings were made from cells visualized by infrared‐differential interference contrast (IR‐DIC) microscopy using an Olympus upright BX51WI microscope with a 40X submersion lens. Patch electrodes (4.0–5.0 MΩ resistance) were pulled on a Sutter P‐97 horizontal puller (Sutter Instrument Company, Novato, CA) from borosilicate glass capillaries (World Precision Instruments, Sarasota, FL). Recorded signals were amplified and filtered (4 kHz low‐pass Bessel filter) using a MultiClamp 700B amplifier (Axon Instruments, Union City, CA) and acquired at ≥10 kHz using a Digidata 1440A analog‐to‐digital board and pClamp10 software (Axon Instruments). Whole‐cell capacitance (Cm), input resistance (RN), series resistance (RS), and resting membrane potential (RMP) values were calculated using pClamp10 software. Only cells with an Rs < 20 MΩ, a RMP more hyperpolarized than −20 mV and RN > 100 MΩ were included in data analysis. Rs values were not significantly different between control iPSC‐derived MNs co‐cultured with control, *C9orf72* or gene edited iPSC‐derived astrocytes. Cells were defined as neurons if they had clear fast‐inactivating inward currents (≥50 pA). Recordings from glial cells were excluded from all analyses. An on‐line P4 leak subtraction protocol was used for all recordings of voltage‐activated currents. Descriptions of voltage and current‐clamp protocols are provided in the results section.

The aCSF used for all electrophysiological recordings contained the following in mM; 127 NaCl, 3 KCl, 2 CaCl_2_, 1 MgSO_4_, 26 NaHCO_3_, 1.25 NaH_2_PO_4_, 10 d‐glucose (equilibrated with 95% O_2_ and 5% CO_2_ at room temperature, pH 7.45; osmolarity, ~ 310 mOsm). The pipette solution contained (in mM): 140 potassium methane‐sulfonate, 10 NaCl, 1 CaCl2, 10 HEPES, 1 EGTA, 3 ATP‐Mg, 0.4 GTP, (pH 7.2–7.3, adjusted with KOH; osmolarity adjusted to ~ 300 mOsm with sucrose).

Electrophysiological data were analyzed using Clampfit10 software (Axon Instruments). Data from control iPSC‐derived MNs co‐cultured with *C9orf72* iPSC‐derived astrocytes lines (3 lines) were pooled for all analyses. Peak Na^+^ currents and peak K^+^ currents (log10 transformed), Cm, RN, and RMP were compared across the three different co‐culture groups using one‐way ANOVAs.

For the purposes of statistical comparisons, action potential generation was classified as either present or absent. These binary data were fitted with a general linear model and contrasts were made using Wald's tests and *p* values adjusted using a Bonferroni correction.

### 2.17 Statistics

At least three independent derivations of astrocytes and MNs were used in each assay. All data are presented as mean ± S.E.M. Difference between means of two groups was analyzed by two‐sided Student's *t*‐test, whereas difference between means of three or more groups were analyzed by one‐way ANOVA with Bonferroni correction or Turkey's post‐hoc test. Two‐way ANOVA was performed where two independent factors were involved. For all analyses, the null hypothesis was rejected at 0.05.

## 3 RESULTS

### 3.1 Generation of functional astrocytes from iPSCs of healthy controls and *C9orf72* ALS patients

Dermal fibroblast derived iPSCs were generated from two healthy individuals (Con‐1 and Con‐2), three ALS patients carrying the *C9orf72* hexanucleotide repeat expansion (C9‐1, C9‐2, and C9‐3) as well as isogenic control lines for C9‐1, C9‐2, and C9‐3 wherein the G_4_C_2_ repeat expansion was corrected using CRISPR/Cas9 mediated genome editing (C9‐Δ) (Selvaraj et al., 2018). All iPSC lines were karyotypically normal, expressed pluripotent stem cell markers and were able to be differentiated in vitro into three germ layers (Figure S1a,b). Repeat‐primed PCR was used to confirm both the presence of G_4_C_2_ repeat expansion in all three *C9orf72* mutant lines and its absence in control and gene corrected lines (Figure S1c).

**Figure 1:**
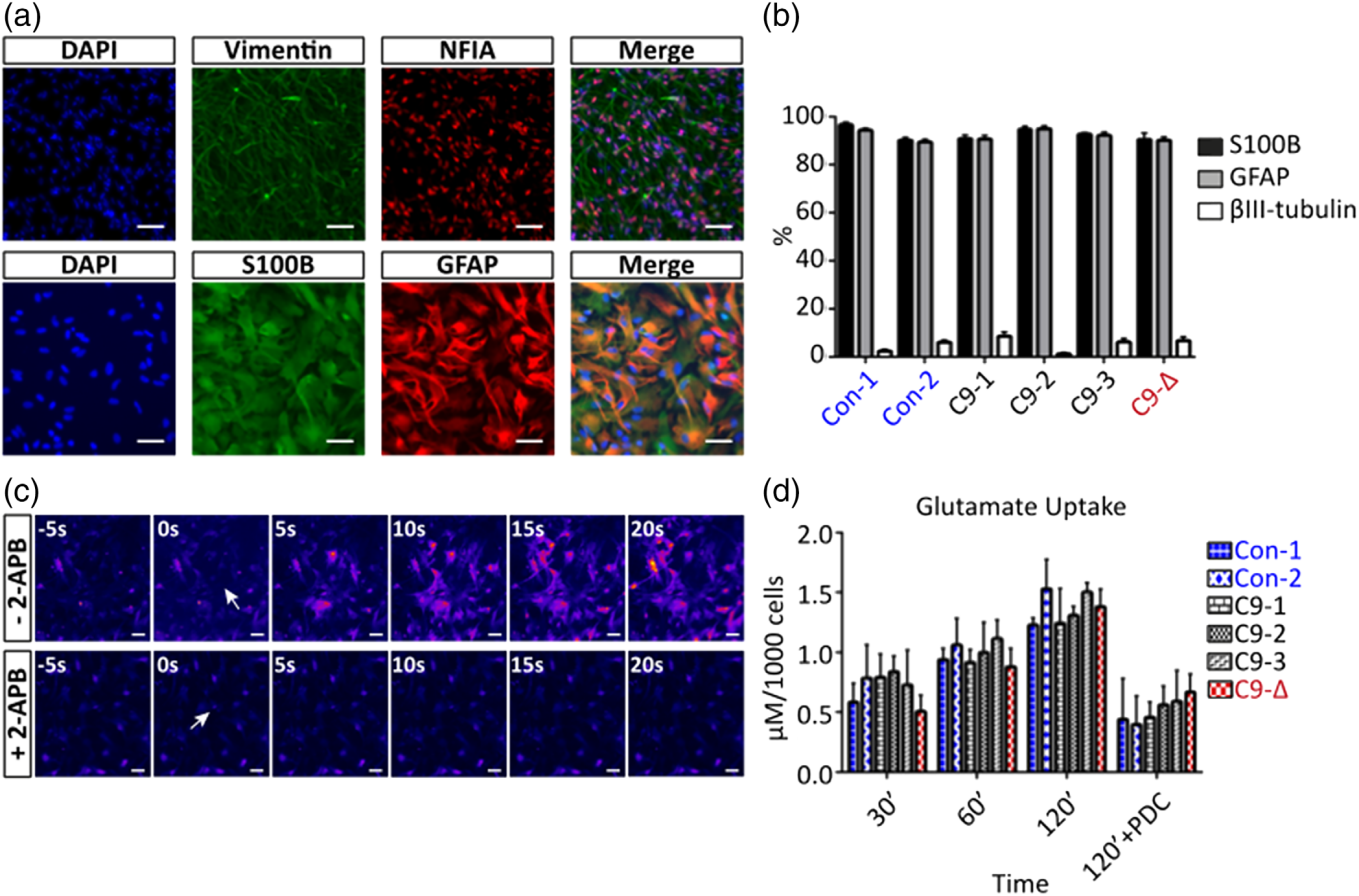
Generation and characterisation of astrocytes from iPSCs. (a) Representative images of vimentin and NFIA immunostaining in astrocyte progenitors (upper panel) and S100B and GFAP immunostaining in 2‐week old astrocyte cultures (lower panel). (Scale bars: 50 μm). (b) Percentage of S100B^+^, GFAP^+^ and βIII‐tubulin^+^ cells in 2‐week old astrocyte cultures derived from various iPSC lines (*N* = 3–7, at least 400 cells per cell line per experiment, one‐way ANOVA with Bonferroni correction). (c) Representative calcium imaging showed that iPSC‐derived astrocytes could propagate calcium waves under mechanical stimulation (upper panel), which was absent in the presence of 2‐APB (lower panel). (Arrows: location of mechanical stimulation at time 0 s; scale bars: 50 μm). (d) The glutamate uptake assay confirmed the capability of glutamate clearance in iPSC‐derived astrocytes without significant difference across various iPSC lines. Application of PDC from 0′ to 120′ was used as a negative control (*N* = 3–6, two‐way ANOVA with Bonferroni correction) [Color figure can be viewed at http://wileyonlinelibrary.com]

We next generated astrocyte progenitor cells (APCs) from iPSC lines and differentiated them into astrocytes using a previously established protocol (Serio et al., 2013). Immunocytochemistry showed high expression of APC markers vimentin and nuclear factor I‐A (NFIA), and quantitative immunolabeling at 2 weeks post differentiation revealed >90% of cells positive for astrocyte markers, S100 calcium‐binding protein B (S100B) and glial fibrillary acidic protein (GFAP). Comparable differentiation efficiency was observed across all six iPSC lines (Figure 1a,b). Functional evaluation of the iPSC‐derived astrocytes was next undertaken. All lines demonstrated propagation of calcium waves upon mechanical stimulation that was blocked by application of 2‐aminoethoxydiphenyl borate (2‐APB), an inhibitor of ionisitol‐3‐phosphate (IP3)‐dependent calcium release (Figure 1c). Astrocytes from all lines also exhibited the ability to take up extracellular L‐glutamic acid in a time‐dependent manner with no differences observed between lines (Figure 1d). Clearance of glutamate was reversed by the glutamate transporter inhibitor L‐trans‐pyrrolidine‐2,4‐dicarboxylic acid (PDC) (Figure 1d). These data demonstrate that the presence of the G_4_C_2_ repeat expansion does not affect differentiation efficiency or basic functional properties of astrocytes.

### 3.2 Mutant astrocytes manifest RNA foci and dipeptide repeats that are reversed upon gene correction

**Figure 2:**
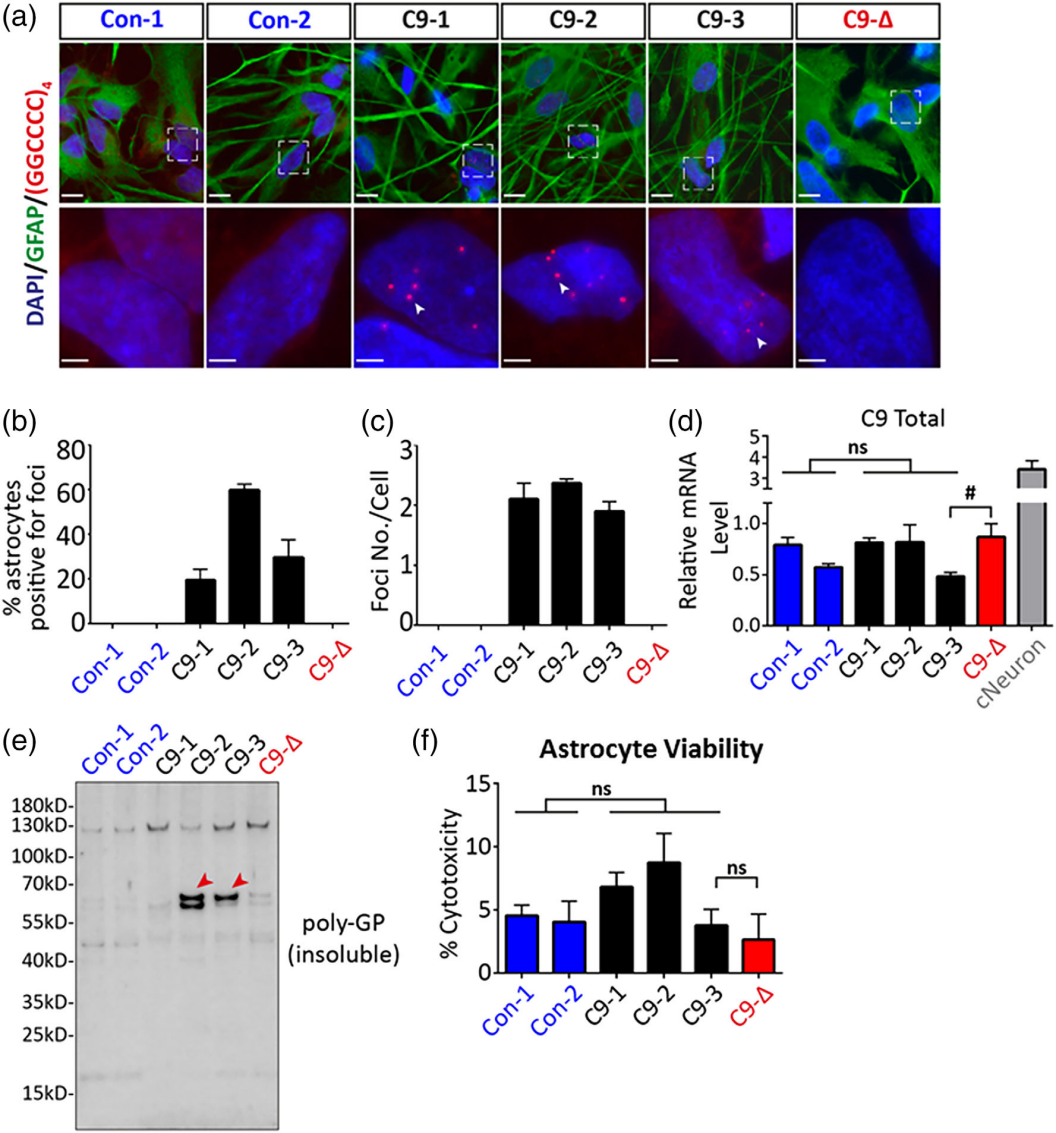
Rescue of mutant specific astrocyte pathology via gene editing. (a) RNA FISH showed nuclear RNA foci (arrow heads) in mutant astrocytes which were abolished in gene edited astrocytes C9‐Δ (cells were co‐stained with an astrocyte marker GFAP; the lower panel shows enlarged images of areas indicated by squares in the upper panel; scale bars, 10 μm—upper panel, 2.5 μm—lower panel). (b) Percentage of 2‐week old astrocytes positive for nuclear RNA foci across various iPSC lines. (c) Average number of RNA foci per cell in 2‐week old astrocytes derived from various iPSC lines. (d) Differentiated human astrocytes had significantly lower *C9orf72* transcript levels compared to cortical neurons, and a significant reduction of *C9orf72* transcript levels was detected when comparing C9‐3 astrocytes to its isogenic control C9‐Δ astrocytes. (ns, not significant, between control and C9; #, *p* < .05, between C9‐3 and C9‐Δ; Student's *t*‐test). (e) A western blot of urea‐soluble protein fraction showed presence of the poly‐GP DPR (indicated by red arrow heads) in C9‐2 and C9‐3 astrocytes, which was absent in the gene edited C9‐Δ astrocytes. (f) A population‐based LDH release assay revealed no differences in viability under basal culture conditions either between control and mutant astrocytes or between the isogenic pair (ns, not significant; Student's *t*‐test) [Color figure can be viewed at http://wileyonlinelibrary.com]

*C9orf72* is believed to cause disease by three putative mechanism(s); haploinsufficiency, sequestration of RNA binding proteins by RNA foci and/or di‐peptide repeat (DPR) mediated toxicity (Mizielinska & Isaacs, 2014; Shi et al., 2018; Tabet et al., 2018). As intranuclear RNA foci are observed in astrocytes in post‐mortem derived material from *C9orf72* patients (Lagier‐Tourenne et al., 2013), we first used fluorescent in situ hybridization (FISH) to confirm the presence of abundant intranuclear RNA foci in mutant astrocytes that were absent in controls (Figure 2a). Foci were absent upon RNase treatment but observed with DNase treatment validating that they are bona‐fide RNA foci (Figure S2). In addition, no foci were detected in *C9orf72* mutant astrocytes when using a probe against the myotonic dystrophy type 2 (DM2) repeat expansion (CCTG)_n_, confirming the specificity of the G_4_C_2_ anti‐sense probe (Figure S2). Quantification of RNA FISH revealed up to 60% of mutant astrocytes contained nuclear foci with no foci observed in control astrocytes (Figure 2b,c). Notably, RNA foci were absent in astrocytes derived from the gene‐corrected C9‐Δ line (Figure 2a–c), demonstrating a direct causal link between *C9orf72* mutation and the formation of RNA foci in astrocytes.

We next examined the transcript levels of total *C9orf72* in iPSC‐derived control and mutant astrocytes. *C9orf72* transcripts detected in astrocytes were almost four‐fold less compared to cortical neurons derived from a control human embryonic stem cell line (Figure 2d). These data are in agreement with the in vivo finding that *C9orf72* is more highly expressed in neurons compared to astrocytes (Jiang et al., 2016; Suzuki et al., 2013). Although no difference between control and mutant astrocytes was evident when data from all lines were pooled, a significant reduction was detected when comparing C9‐3 astrocytes directly to its isogenic control C9‐Δ astrocytes (Figure 2d; C9‐3, 0.482 ± 0.039, *n* = 5; C9‐Δ, 0.868 ± 0.129, *n* = 4; *p* < .05, Student's *t*‐test). We further performed western blot analysis in iPSC derived astrocytes and did not observe reduction in C9ORF72 protein levels (Figure S3).

Recent reports have shown that the G_4_C_2_ repeat expansions are translated by repeat‐associated non‐ATG (RAN) translation generating five different di‐peptide repeats (Ash et al., 2013; Donnelly et al., 2013; Mori et al., 2013). Using a commercially available antibody detecting poly‐GP DPR, we performed western blot analysis on urea‐soluble protein fractions isolated from control and mutant astrocyte samples. Two bands of ~60 kD were detected only in C9‐2 and C9‐3 astrocytes but not in control astrocytes (Figure 2e). Importantly, the bands found in C9‐3 astrocytes were absent in astrocytes derived from its isogenic control C9‐Δ (Figure 2e). However, no poly‐GA and poly‐PA DPR was detected in mutant astrocytes (data not shown).

TDP‐43 proteinopathy is a pathological hallmark of ALS with cytoplasmic misaccumulation of TDP‐43 found in MNs and glial cells (Arai et al., 2006; Ling, Polymenidou, & Cleveland, 2013; Neumann et al., 2006). However, using immunocytochemical labeling of TDP‐43 we found predominantly nuclear localization (Figure S4a) with no difference in nuclear or cytoplasmic TDP‐43 intensity upon densitometric analysis between control and mutant astrocytes, or between C9‐3 and C9‐Δ astrocytes (Figure S4b,c). In addition, quantitative immunoblot analysis showed equivalent protein levels of soluble TDP‐43 in astrocytes derived from all six iPSC lines (Figure S4d,e).

### 3.3 Mutant astrocytes cause control MNs to lose functional output without overtly effecting cell viability

**Figure 3:**
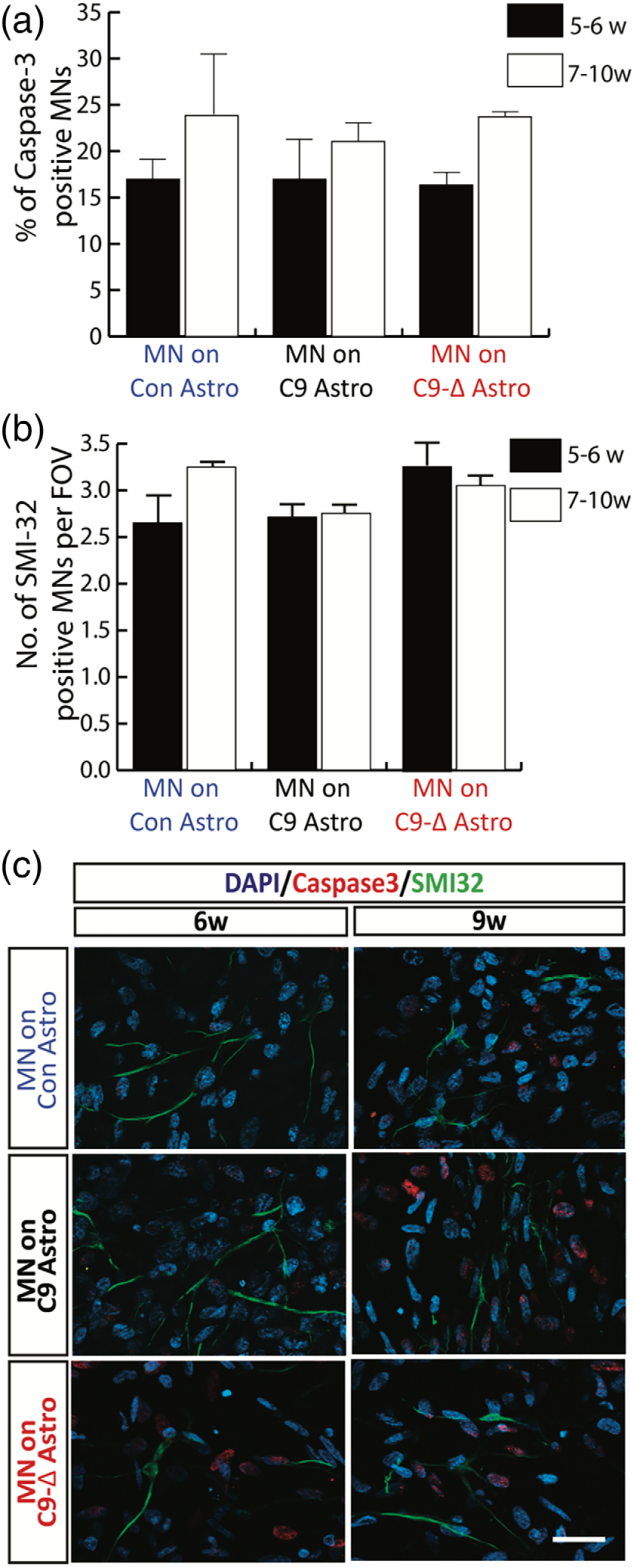
Mutant astrocytes have no clear impact on MN viability. (a,b) Quantitative analysis of the apoptotic marker caspase‐3 (a) and the MN marker SMI‐32 (b) reveal no effect of mutant astrocytes on MN viability in long‐term cultures (20 fields of view [F.O.V] per time‐point per line for minimum of 3 differentiations, one‐way ANOVA). (c) Representative images of Caspase‐3 and SMI‐32 immunostaining in MNs co‐cultured with control, mutant and gene‐edited astrocytes at 6 and 9 weeks post MN plating (scale bars: 20 μm) [Color figure can be viewed at http://wileyonlinelibrary.com]

Accumulating evidence from pathological and experimental studies suggests that astrocytes may both undergo degeneration in ALS (Serio et al., 2013; Tong et al., 2013) and exert toxic effects on MNs (Ilieva et al., 2009). To first address whether G_4_C_2_ expansion adversely affects the viability of isolated astrocytes, we undertook LDH assays that showed no difference between control and *C9orf72* mutant astrocytes (Figure 2f; ns, not significant; student t‐test). We next co‐cultured mutant astrocytes with wild‐type MNs and determined MN viability by quantitative caspase‐3 and SMI‐32 counts. No difference in MN survival was found even in co‐cultures maintained for up to 10 weeks post‐plating (Figure 3a–c; ns, not significant; one‐way ANOVA).

In view of the absence of any viability differences in co‐cultures and our previous finding of mutant iPSC‐derived MNs demonstrating physiological dysfunction prior to any changes in survival (Devlin et al., 2015), we next examined whether mutant astrocytes affect MN function. To facilitate comparisons with our previous study (Devlin et al., 2015), MNs derived from the same control iPSC line (Con‐2) that we used in our previous work were co‐cultured with astrocytes for up to 10 weeks post‐plating. Electrophysiological analyses were used to investigate whether patient iPSC‐derived astrocytes had any effect on the function of control MNs. Whole‐cell patch‐clamp recordings were obtained from the largest neurons visualized via IR‐DIC microscopy in the co‐cultures from 3 to 10 weeks post MN plating. Selecting the largest neurons ensured recordings were predominantly obtained from MNs (Devlin et al., 2015).

We first compared the passive membrane properties of control MNs co‐cultured with astrocytes from a healthy individual (Con‐2), three ALS patients carrying the *C9orf72* hexanucleotide repeat expansion (C9‐1, C9‐2, and C9‐3) as well as an isogenic control line for C9‐3 (C9‐Δ). For these and all other electrophysiological analyses, data were pooled for control MNs co‐cultured with mutant astrocytes (see Figure S5 for data from individual lines). At weeks 3–4 post‐plating, whole‐cell capacitance (*C*_m_) values were similar across MNs plated on control, mutant and gene edited‐astrocytes (see Table 1 for $\bar{x}\;$ ± *SEM* and sample sizes; one‐way ANOVA with Tukey's honest significant difference). From weeks 5–10, MNs plated on mutant astrocytes had smaller *C*_m_ values compared to those on gene‐edited astrocytes and from weeks 7–10 compared to MNs on control astrocytes (Table 1). Input resistance (*R*_N_) values were similar in MNs co‐cultured with control, mutant and gene‐edited astrocytes throughout the time period studied (Table 1). MNs co‐cultured with mutant astrocytes had a more depolarized resting membrane potential (RMP) at weeks 3–4 compared to MNs co‐cultured with gene‐edited astrocytes (Table 1; *p* < .05, one‐way ANOVA with Tukey's honest significant difference). However, resting membrane potential did not differ at other time points in co‐culture. These findings indicate that *C9orf72* patient iPSC‐derived astrocytes cause time‐dependent changes to some of the passive membrane properties of control iPSC‐derived MNs.

**Table 1:** Passive membrane properties

Passive membrane properties	MNs on control astros	MNs on C9 astros	MNs on C9‐Δ astros
***C***_**m**_ **(pF)**			
Weeks 3–4	23.8 ± 0.9 (*n* = 50)	26.2 ± 0.8 (*n* = 153)	27.3 ± 1.1 (*n* = 74)
Weeks 5–6		25.6 ± 1.4 (*n* = 53)^††††^	33.4 ± 1.6 (*n* = 49)
Weeks 7–10	27.2±1.6 (n=43)	20.2 ± 1.1* (*n* = 61)^***††††^	31.2 ± 2.6 (*n* = 33)
***R***_**N**_ **(MΩ)**			
Weeks 3–4	540 ± 45	579 ± 38	564 ± 46
Weeks 5–6		377 ± 38	391 ± 44
Weeks 7–10	457 ± 48	514 ± 55	470 ± 62
**RMP (mV)**			
Weeks 3–4	−48.3 ± 1.9	−49.5 ± 1.2	−42.0 ± 1.7
Weeks 5‐6		−44.0 ± 1.8†	−50.2 ± 2.2
Weeks 7‐10	−49.8 ± 2.1	−43.1 ± 1.5	−43.4 ± 2.4

* Significantly different to controls (*** *p* < .001; one way ANOVA with Tukey's honest significant difference).

† Significantly different to the gene‐edited line C9‐Δ (^†^ *p* < .05; ^††††^ *p* < .0001; one way ANOVA with Tukey's honest significant difference).

**Figure 4:**
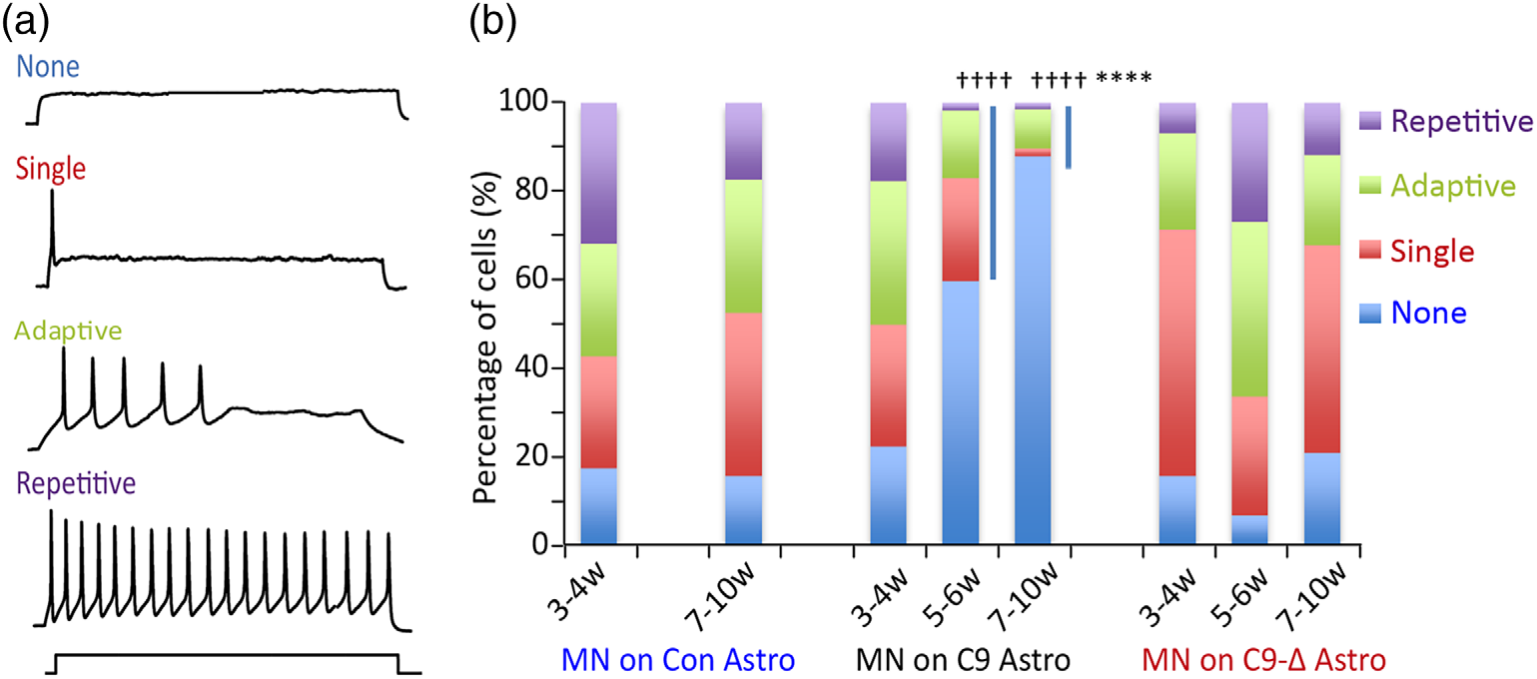
iPSC‐derived astrocytes from ALS patients cause loss of functional output in healthy control iPSC‐derived MNs. (a) Representative examples of the four categories of firing observed in iPSC‐derived MNs (repetitive, adaptive, single or no firing). (b) Percentage of cells exhibiting each firing category in MNs co‐cultured with astrocytes derived from various iPSC lines across weeks 3–10 post plating (Weeks 3–4: Control, *n* = 47; C9, *n* = 151; C9‐Δ, *n* = 73; Weeks 5–6: C9, *n* = 38; C9‐Δ, *n* = 48; Weeks 7–10: Control, *n* = 40; C9, *n* = 58; C9‐Δ *n* = 34; ****, *p* < .0001, significantly different to MNs on control astrocytes; ^††††^, *p* < .0001, significantly different to MNs on the gene‐edited C9‐Δ astrocytes; general linear model with multiple Wald's tests and Bonferroni correction) [Color figure can be viewed at http://wileyonlinelibrary.com]

As reported previously (Devlin et al., 2015), current injection elicited four output patterns described as repetitive, adaptive, single, and no firing, in MNs cultured on astrocytes (Figure 4a). Repetitive firing was defined as a train of action potentials that lasted for the duration of the square current injection (1 s), while adaptive firing was defined as multiple action potentials that stopped before the end of the current stimuli. Cells defined as having an adaptive output pattern were unable to repetitively fire in response to any of the series of current steps applied. In order to compare the excitability of repetitively firing MNs co‐cultured with control, mutant or gene‐edited astrocytes, frequency‐current (*f‐I*) relationships were generated from responses to a series of injected current steps (0 to 145 pA, in 10 pA increments, 1 s duration). Comparisons were performed on data pooled from recordings of repetitively firing cells at weeks 2–6 post MN plating. Analyses of the slope of the combined *f‐I* relationship found no differences between MNs co‐cultured with control, mutant or gene‐edited astrocytes. However, rheobase current was greater in MNs plated on mutant astrocytes compared to MNs plated on control astrocytes (control, $\bar{x}$ 15.6 ± *SEM* 1.4 pA, *n* = 16; mutant, 26.1 ± 3.2, *n* = 26; gene‐edited, 22.3 ± 1.2, *n* = 15; *p* < .05, one‐way ANOVA), suggesting some degree of hypoexcitability in MNs co‐cultured with mutant astrocytes (data not shown). Clear evidence of hypoexcitability (reduced output) was next revealed when the firing patterns of control iPSC‐derived MNs co‐cultured with control, mutant or gene‐edited astrocytes were compared. The relative proportions of firing versus non‐firing cells were similar in all cultures from weeks 3–4 post MN plating (Figure 4b; control firing, 82.9%, *n* = 47; mutant firing, 78.1%, *n* = 151; gene‐edited firing, 84.9%, *n* = 73). However, at weeks 5–6 and 7–10 post MN plating, the number of cells able to fire action potentials decreased significantly in MNs co‐cultured with mutant astrocytes while the ratio of firing versus non‐firing cells remained unchanged in MNs co‐cultured with control or gene‐edited astrocytes throughout these time‐points (Figure 4b; Weeks 5–6: mutant firing, 40.3%, *n* = 38; gene‐edited firing, 93.7%, *n* = 48; *p* < .0001; Weeks 7–10: control firing, 85%, *n* = 40; mutant firing, 12.0%, *n* = 58; gene‐edited firing, 79.4%, *n* = 34; *p* < .0001, general linear model with multiple Wald's tests and Bonferroni correction). These data demonstrate a clear loss of functional output in control MNs co‐cultured with mutant astrocytes compared to control or gene edited astrocytes, consistent with the idea that mutant astrocytes alone are sufficient to cause physiological toxicity to MNs.

### 3.4 Mutant astrocytes cause loss of voltage‐activated currents in control MNs

**Figure 5:**
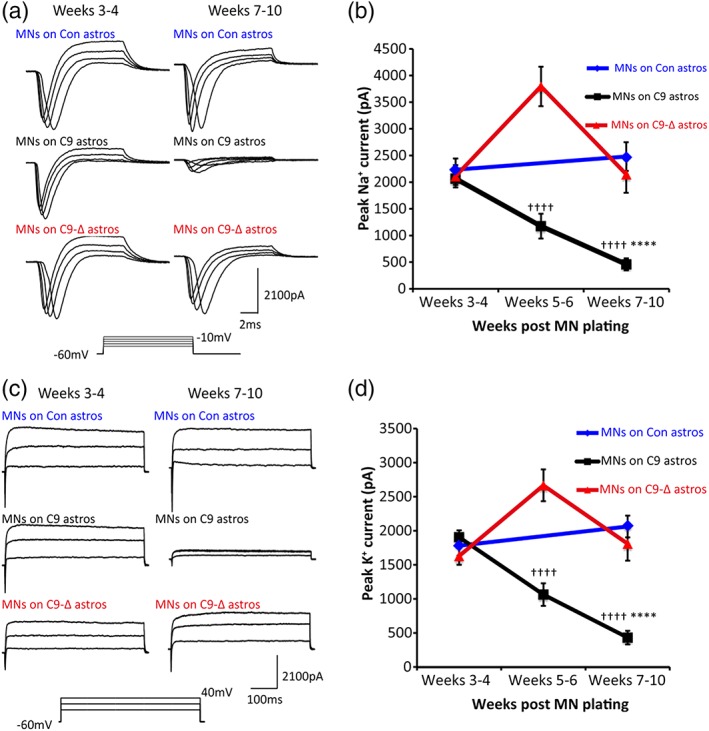
Loss of fast, inactivating Na^+^ currents and persistent K^+^ currents in control iPSC‐derived MNs co‐cultured with ALS patient iPSC‐derived astrocytes. (a) Raw data showing fast, inactivating Na^+^ currents in control iPSC‐derived MNs co‐cultured with astrocytes derived from various iPSC lines at weeks 3–4 and weeks 7–10 post plating. (b) Peak fast, inactivating Na^+^ currents plotted from weeks 3–10 for MNs co‐cultured with control (*n* = 93), C9 (*n* = 266) and C9‐Δ (*n* = 156) iPSC‐derived astrocytes (Weeks 3–4: Control, *n* = 49; C9, *n* = 153; C9‐Δ, *n* = 74; Weeks 5–6: C9, *n* = 53; C9‐Δ, *n* = 49; Weeks 7–10: Control, *n* = 44; C9, *n* = 60; C9‐Δ *n* = 33; ****, *p* < .0001, significantly different to MNs on control astrocytes; ^††††^, *p* < .0001, significantly different to MNs on the gene‐edited C9‐Δ astrocytes; one‐way ANOVA after log transformation with Tukey's post‐hoc test). (c) Raw data showing persistent K^+^ currents in control iPSC‐derived MNs co‐cultured with astrocytes derived from various iPSC lines at weeks 3–4 and weeks 7–10 post plating. (d) Peak persistent K^+^ currents plotted from weeks 3–10 for MNs co‐cultured with control (*n* = 93), C9 (*n* = 266) and C9‐Δ (*n* = 156) iPSC‐derived astrocytes (Weeks 3–4: Control, *n* = 49; C9, *n* = 153; C9‐Δ, *n* = 74; Weeks 5–6: C9, *n* = 53; C9‐Δ, *n* = 49; Weeks 7–10: Control, n = 44; C9, *n* = 60; C9‐Δ *n* = 33; ****, *p* < .0001, significantly different to MNs on control astrocytes; ^††††^, *p* < .0001, significantly different to MNs on the gene‐edited C9‐Δ astrocytes; one‐way ANOVA after log transformation with Tukey's post‐hoc test) [Color figure can be viewed at http://wileyonlinelibrary.com]

To investigate the mechanisms underlying the progressive loss of action potential output in control MNs co‐cultured with mutant astrocytes, voltage‐clamp recordings were performed to assess voltage‐activated currents involved in action potential generation. Fast inactivating Na^+^ currents were first investigated by using a series of voltage steps (−70 to 20 mV, 2.5 mV increments, 10 ms duration) from a holding potential of −60 mV (Figure 5a). We found no differences in the current (*I*–*V*) relationships (Figure S6a) or peak Na^+^ currents between MNs co‐cultured with control, mutant or gene‐edited astrocytes at 3–4 weeks post MN plating (Figure 5b; peak current: control, $\bar{x}$ 2,232 ± s.e.m. 211 pA, *n* = 49; mutant, 2,067 ± 120 pA, *n* = 153; gene‐edited 2,110 ± 209 pA, *n* = 74). However, from weeks 5–10 post MN plating, there was a progressive decrease in peak Na^+^ currents in MNs co‐cultured with mutant astrocytes compared to co‐cultures with control or gene‐edited astrocytes (Figure 5b and Figure S6b; Weeks 5–6: mutant $\bar{x}$ 1,174 ± s.e.m. 233 pA, *n* = 53; gene‐edited, 3,795 ± 371, *n* = 49; Weeks 7–10: control, 2,480 ± 268 pA, *n* = 44; mutant, 458 ± 113 pA, *n* = 60; gene‐edited 2,147 ± 345 pA, n = 33; *p* < .0001, one‐way ANOVA after log transformation with Tukey's post‐hoc test).

We next investigated whether the progressive loss of Na^+^ currents reflected a more general decrease in voltage‐activated currents in control MNs co‐cultured with mutant astrocytes. Persistent K^+^ currents were measured by using a series of voltage steps (−70 to 40 mV, 10 mV increments, 500 ms duration) from a holding potential of −60 mV (Figure 5c). At weeks 3–4 post MN plating, peak K^+^ currents were comparable in MNs co‐cultured with control, mutant or gene‐edited astrocytes (Figure 5d and Figure S6c; Peak current: control, $\bar{x}$ 1,788 ± s.e.m. 148 pA, *n* = 46; mutant, 1913 ± 98 pA, *n* = 147; gene‐edited, 1,634 ± 127 pA, *n* = 74). Similar to Na^+^ currents, peak K^+^ currents progressively declined in control MNs co‐cultured with mutant astrocytes from weeks 5–10 compared to MNs co‐cultured with control or gene‐edited astrocytes (Figure 5d and Figure S6d; Weeks 5–6: mutant, $\bar{x}$ 1,070 ± *SEM* 165 pA, *n* = 53; gene‐edited, 2,673 ± 233 pA, *n* = 43; Weeks 7–10: control, 2069 ± 160 pA, *n* = 41; mutant, 438 ± 99 pA, n = 60; gene‐edited, 1816 ± 248 pA, *n* = 33; *p* < .0001, one‐way ANOVA after log transformation with Tukey's post‐hoc test).

### 3.5 Functional perturbations in *C9orf72* mutant MNs are mediated by mutant astrocytes

We, and others, have previously shown that mutant *C9orf72* MN cultures demonstrate functional perturbations (Devlin et al., 2015; Naujock et al., 2016; Sareen et al., 2013). However, all these studies, including our own (Devlin et al., 2015), used MN generation protocols that also resulted in the production of a significant fraction of astrocytes (up to approximately 20%). One interpretation of these earlier findings is therefore that the observed pathophysiological phenotype was a consequence of contaminant astrocytes and not cell processes intrinsic to MNs. To address this possibility, we next used a recently published method to generate highly enriched mutant *C9orf72* MN cultures with negligible astrocyte contamination (Maury et al., 2015; Selvaraj et al., 2018). In cultures derived from the *C9orf72* ALS patient lines C9‐1 & C9‐3 and their respective gene edited controls (C9‐Δ1 & C9‐ Δ3), we assessed firing output and voltage‐gated Na^+^ and K^+^ currents using the same protocols described above for co‐culture experiments.

**Figure 6:**
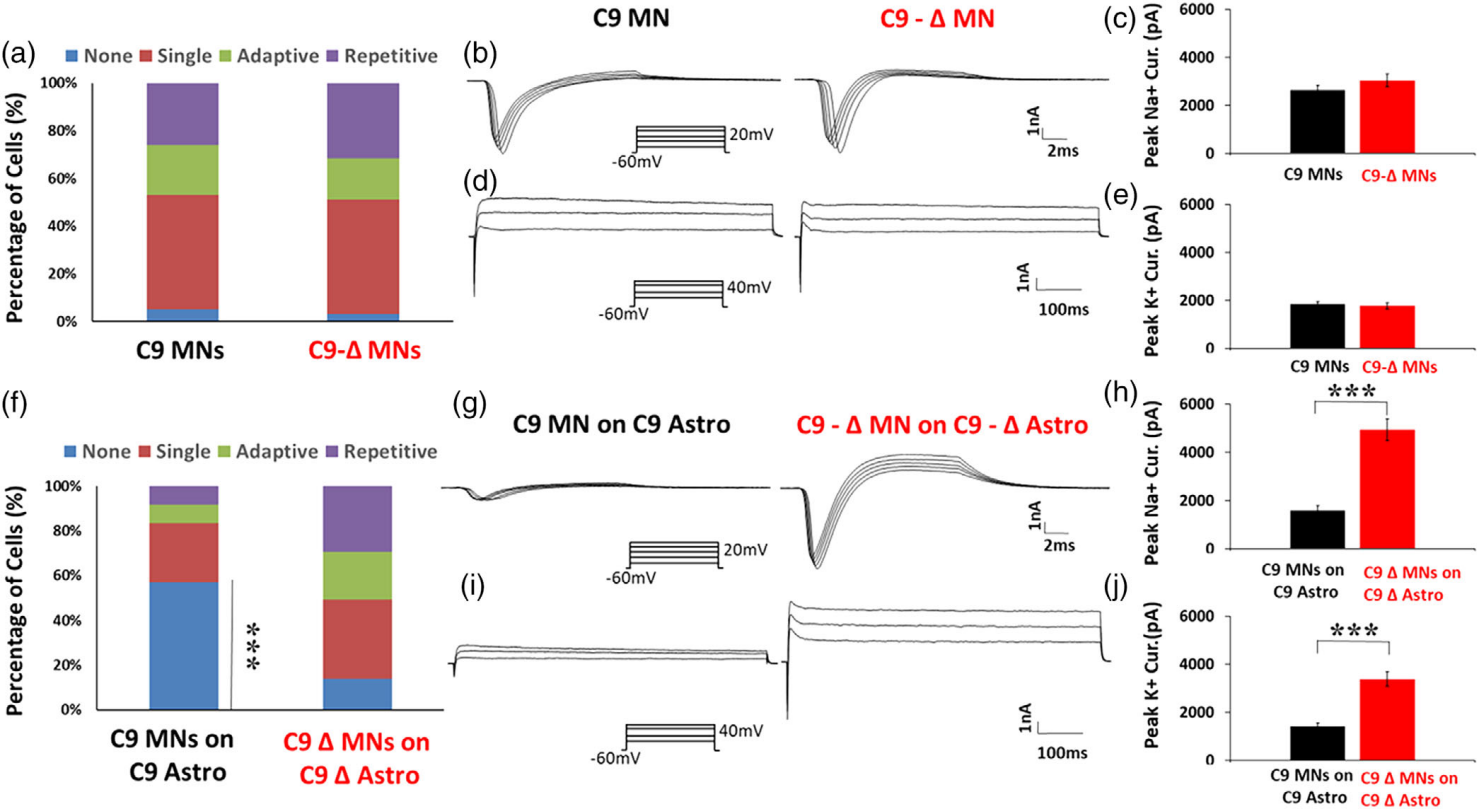
MN‐enriched cultures alone fail to recapitulate the pathophysiology observed when MNs are co‐cultured with mutant astrocytes. (a) Percentage of cells exhibiting each firing category in MN‐enriched cultures derived from mutant and gene‐edited patient iPSC lines across weeks 7–12 post plating (C9, *n* = 115; C‐1, *n* = 49; C‐3, *n* = 66, C9‐Δ, *n* = 92; C‐Δ1, *n* = 19; C‐Δ3, *n* = 73, fisher's exact test). (b) Raw data showing fast, inactivating Na^+^ currents in mutant and gene‐edited iPSC‐derived MNs in MN‐enriched cultures at weeks 7–12. (c) Peak fast, inactivating Na^+^ currents plotted at weeks 7–12 post‐plating for mutant and gene‐edited iPSC‐derived MNs in MN‐enriched cultures (C9, *n* = 110; C‐1, *n* = 48; C‐3, *n* = 62, C9‐Δ, *n* = 82; C‐Δ1, *n* = 17; C‐Δ3, *n* = 65; two tailed, equal variance, non‐paired, student *t*‐test). (d) Raw data showing persistent K^+^ currents in mutant and gene‐edited iPSC‐derived MNs in MN‐enriched cultures at weeks 7–12. (e) Peak persistent K^+^ currents plotted at weeks 7–12 post‐plating for mutant and gene‐edited iPSC‐derived MNs in MN‐enriched cultures (C9, *n* = 110; C‐1, *n* = 48; C‐3, *n* = 62, C9‐Δ, *n* = 82; C‐Δ1, *n* = 17; C‐Δ3, *n* = 65; two tailed, equal variance, non‐paired, student *t*‐test). (f) Percentage of cells exhibiting each firing category in mutant and gene‐ edited MNs co‐cultured with astrocytes derived from mutant and gene‐edited patient iPSC lines respectively, across weeks 7–12 weeks post plating (C9, *n* = 84; C‐2, *n* = 34; C‐3, *n* = 50, C9‐Δ, *n* = 65; C‐Δ2, *n* = 29; C‐Δ3, *n* = 36; ^††††^, *p* < .0001, significantly different to gene‐edited MNs on gene‐edited C9‐Δ astrocytes; fisher's exact test). (g) Raw data showing fast, inactivating Na^+^ currents in mutant and gene‐edited iPSC‐derived MNs from MN‐enriched cultures co‐cultured with mutant and gene‐edited astrocytes respectively at weeks 7–12. (h) Peak fast, inactivating Na^+^ currents plotted at weeks 7–12 post‐plating for MN‐enriched mutant and gene‐edited cultures co‐cultured with mutant and gene‐edited astrocytes respectively (C9, *n* = 78; C‐2, *n* = 31; C‐3, *n* = 47, C9‐Δ, *n* = 64; C‐Δ2, *n* = 27; C‐Δ3, *n* = 37; ^††††,^ *p* < .0001, significantly different to gene‐edited MNs on gene‐edited C9‐Δ astrocytes; student t test, two tailed, non‐paired, unequal variance). (i) Raw data showing persistent K^+^ currents in mutant and gene‐edited iPSC‐derived MNs from MN‐enriched cultures co‐cultured with mutant and gene‐edited astrocytes respectively at weeks 7–12. (j) Peak persistent K^+^ currents plotted at weeks 7–12 post‐plating for MN‐enriched mutant and gene‐edited MN cultures co‐cultured with mutant and gene‐edited astrocytes respectively (C9, *n* = 78; C‐2, *n* = 31; C‐3, *n* = 47, C9‐Δ, *n* = 64; C‐Δ2, *n* = 27; C‐Δ3, *n* = 37; ^††††^, *p* < .0001, significantly different to gene‐edited MNs on gene‐edited C9‐Δ astrocytes; student t test, two tailed, non‐paired, unequal variance) [Color figure can be viewed at http://wileyonlinelibrary.com]

Even after 7–12 weeks of culture, we found no difference in the proportion of cells able to fire action potentials in *C9orf72* versus gene‐edited MN cultures (Figure 6a; Weeks 7–12: mutant firing, 94.8%, *n* = 115; gene‐edited firing, 96.7%, *n* = 92; fisher's exact test). We also observed equivalent voltage gated Na^+^ and K^+^ currents in recordings from *C9orf72*, and gene‐edited MN cultures at weeks 7–12 (Figure 6b–e; Peak Na^+^ current: mutant, $\bar{x}$ 2,641 ± s.e.m. 201 pA, *n* = 110; gene‐edited, 3,043 ± 267 pA, *n* = 82; Peak K^+^ currents: mutant, 1,844 ± 108 pA, *n* = 110; gene‐edited, 1,772 ± 126 pA, *n* = 82; two tailed, equal variance, unpaired student *t*‐test).

Next, to ensure that the lack of pathophysiology was not related to differences in differentiation protocols, we co‐cultured these same highly enriched *C9orf72* (C9‐2 & C9‐3) and gene‐edited (C9‐Δ2 & C9‐Δ3) MNs with mutant (C9‐2, C9‐3) and gene‐corrected (C9‐Δ2, C9‐Δ3) astrocytes respectively. The presence of mutant, but not gene‐corrected astrocytes, was again sufficient to induce altered function in MNs as evidenced by a reduction in the proportion of cells able to fire action potentials (Figure 6f; Weeks 7–12; mutant firing, 42.8%, *n* = 84; gene‐edited firing, 86.1%, *n* = 65; *p* < .0001, fisher's exact test) and decreases in Na^+^ and K^+^ currents (Figure 6g–j; Peak Na^+^ current: mutant, $\bar{x}$ 1,582 ± s.e.m. 212 pA, *n* = 78; gene‐edited, 4,938 ± 448pA, *n* = 64; Peak K^+^ currents: mutant, 1,407 ± 150 pA, *n* = 78; gene‐edited, 3,387 ± 299 pA, *n* = 64; *p* < .0001, Mann Whitney test, unpaired). Together these findings demonstrate that mutant astrocytes, upon co‐culture, directly mediate perturbations in the physiological properties of control and mutant *C9orf72* MNs.

### 3.6 Multiple gene pathways are perturbed in *C9orf72* astrocytes

**Figure 7:**
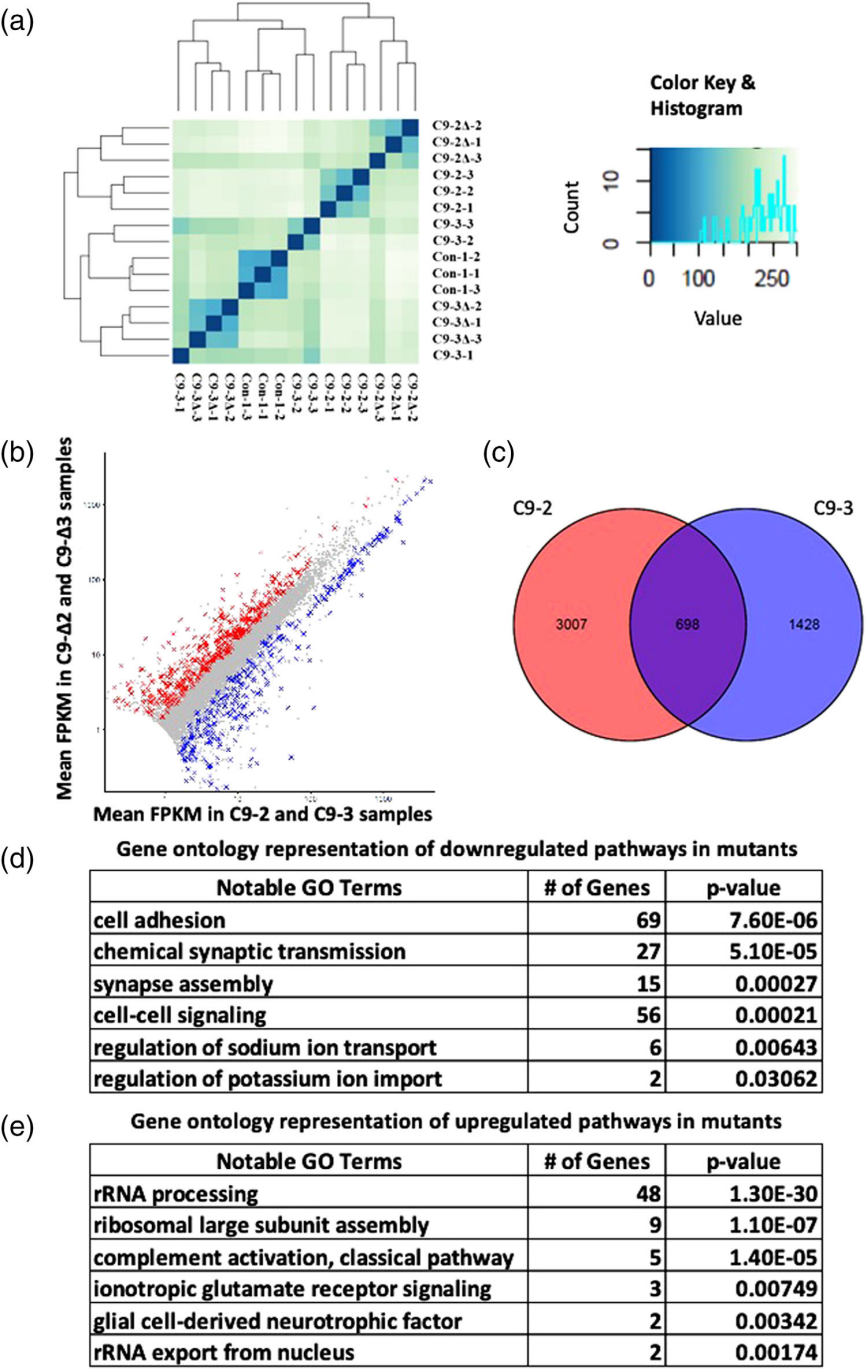
RNA Seq analysis of C9ORF72 astrocytes. (a) Heat map and dendrogram depicting hierarchical clustering of RNA sequencing reads from control (Con‐1), C9 mutant (C9‐2, C9‐3), and respective isogenic control (C9‐Δ2, C9‐Δ3) astrocytes. (b) Scatter plot showing comparison of transcriptome reads between 2 different C9 mutants and isogenic controls. Red crosses denote significantly upregulated genes and blue crosses denote significantly downregulated genes (*p* < 0.05, analysis performed using DESeq 2). (c) Venn diagram indicating total no. of genes differentially expressed between each C9ORF72 mutant and isogenic control astrocyte out of which 698 genes were found to be common between both C9 and isogenic pair. (d, e) Go ontology studies predicted pathways including significantly downregulated (d) and upregulated (e) genes in *C9orf72* astrocytes [Color figure can be viewed at http://wileyonlinelibrary.com]

In order to probe the cellular processes disrupted in *C9orf72* astrocytes we next undertook RNA sequencing on astrocytes derived from two independent *C9orf72* mutant iPSCs (C9‐2 & C9‐3) and their corresponding isogenic controls (C9‐Δ2 & C9‐Δ3). Heat maps and hierarchical clustering of the transcriptomic data obtained from astrocytes demonstrated greater differences between the two mutant lines than between mutant line and its corresponding isogenic corrected lines owing to heterogeneity across different iPSC lines. Therefore, to overcome this transcriptional heterogeneity we performed comparisons between each mutant and its corresponding isogenic control (Figure 7a). Differentially expressed genes were filtered using the following criteria: (a) genes must have significant differential expression in the 2 independent mutant lines when compared to respective independent isogenic controls (false discovery rate < 0.1 for each isogenic pair), (b) genes must be dysregulated in the same direction across both lines, (c) genes were also filtered to only retain those with mean fragments per kilobase per million (FPKM) > 1 (measured across all samples, which approximates to 0.5–1 mRNA per cell). Using this approach 698 dysregulated genes were identified (Figure 7b,c & Figures S8 and S9). Gene ontology analysis revealed that genes including those involved in ionotropic glutamate receptor signaling (GRIA1, GRIA4), complement activation, ribosomal subunit assembly (large and small) and nuclear RNA export were significantly upregulated in mutant *C9orf72* astrocytes. Downregulated genes in mutant *C9orf72* astrocytes included genes involved in cell adhesion (L1CAM, TSP1, NTN1), synapse assembly (BDNF, NRG1, THBS2), cell‐to‐cell signaling (GPC6), regulation of sodium ion transport (SLC8A1, ATP1B2, NKAIN4) and potassium ion import (DLG1, ATP1B2). These novel transcriptomic data reveal changes in multiple pathways that may contribute to the deleterious effects of *C9orf72* astrocytes on MN function.

## 4 DISCUSSION

Here, we show that expression of the *C9orf72* mutation in astrocytes recapitulates key aspects of *C9orf72*‐related ALS pathology and directly results in physiological dysfunction of control and *C9orf72* MNs upon co‐culture, thus highlighting both cell‐autonomous astrocyte pathology and non‐cell autonomous MN pathophysiology.

*C9orf72* mutant iPSC derived astrocytes displayed key pathological features of RNA foci and poly‐GP DPR. The loss of foci and DPR in gene‐edited astrocytes directly links the G4C2 repeat expansion with formation of RNA foci and poly‐GP DPR in human astrocytes. It remains to be determined whether other DPRs, in addition to poly‐GP, are also produced in patient iPSC‐derived astrocytes. In line with previous in vivo findings we observed relatively low levels of *C9orf72* transcripts and protein in astrocytes (Jiang et al., 2016). Although TDP‐43 proteinopathies are the pathological hallmark of ALS regardless of patients' genotypes, with TDP‐43 deposition observed in astrocytes in post‐mortem tissues (Yamanaka & Komine, 2018) no post‐mortem studies have yet reported TDP‐43 proteinopathies in astrocytes in *C9orf72*‐related ALS. Our previous study has shown that iPSC‐derived astrocytes carrying a *TARDBP* mutation do not display TDP‐43 aggregates or loss of nuclear TDP‐43 despite increased cytoplasmic TDP‐43 expression (Serio et al., 2013), nor do we detect TDP‐43 proteinopathies in astrocytes harboring a *C9orf72* mutation in the present study, suggesting that iPSC‐derived astrocytes may not manifest all TDP‐43 proteinopathies in vitro. Additionally, in the AAV‐G4C2‐66 mice, only 7–8% of cells in cortex and hippocampus display phosphorylated TDP‐43 inclusions (Chew et al., 2015), and no TDP‐43 aggregations were observed in BAC‐C9 (100–1,000) mice (O'Rourke et al., 2015).

Although there is consistent evidence of non‐cell autonomous toxicity mediated by astrocytes harboring *SOD1* mutations (Ilieva et al., 2009; Marchetto et al., 2008; Nagai et al., 2007; Papadeas et al., 2011; Tripathi et al., 2017; Tyzack et al., 2017), data are either lacking or conflicting for other ALS‐related mutations (Haidet‐Phillips et al., 2013; Serio et al., 2013; Tong et al., 2013). For example, human iPSC‐derived astrocytes from a patient harboring an *TARDBP* M337V mutation did not affect the survival of control iPSC‐derived MNs (Serio et al., 2013). This finding was supported by an independent study where astrocytes lacking TDP‐43 or overexpressing mutant *TARDBP* failed to cause the death of control MNs in co‐culture or when implanted into wild‐type rat spinal cords (Haidet‐Phillips et al., 2013). Conversely, wild‐type MNs in transgenic rats, where the *TARDBP* M337V mutation was restricted to astrocytes, progressively degenerate (Tong et al., 2013). Furthermore, cell culture experiments have shown that control MNs degenerate when exposed to astrocyte‐conditioned medium (ACM) collected from cultures of mouse astrocytes harboring mutant TDP‐43 (Rojas et al., 2014) or sporadic ALS patient astrocytes (Haidet‐Phillips et al., 2011).

Our finding, that *C9orf72* mutant astrocytes cause progressive dysfunction of control MNs, strongly support non‐cell autonomous disease mechanisms in *C9orf72*‐mediated ALS. Although we did not find an effect of *C9orf72* mutant astrocytes on MN survival, Meyer et al. previously reported that, upon co‐culture, mutant human *C9orf72* astrocytes led to the loss of control mouse MNs (Meyer et al., 2014). However, important differences between the studies limit direct comparison. These include the method of generation and regional identity of astrocytes. Meyer et al. derived astrocytes through direct conversion of adult skin fibroblasts into neural precursors, which were subsequently differentiated into astrocytes in serum‐containing media without patterning. In the current study, fibroblasts were first reprogrammed into iPSCs before generating spinal astrocytes in chemically defined media. These differences in generation methods can lead to distinct epigenetic and transcriptional patterns with functional consequences (Chandrasekaran, Avci, Leist, Kobolak, & Dinnyes, 2016). Critically, Meyer et al. also studied mouse ESC‐derived MNs in contrast to an entirely humanized co‐culture model as reported in this study. This is an important consideration, given that astrocytes derived from human versus rodent iPSCs exhibit different transcriptomic profiles and subtle functional differences (Y. Zhang et al., 2016). Notwithstanding experimental differences, both the present study and that of Meyer and colleagues demonstrate adverse effects of *C9orf72* mutant astrocytes on MNs, strongly supporting involvement of non‐cell autonomous disease mechanisms in *C9orf72*‐mediated ALS. Furthermore, our findings highlight the importance of investigating function as well as cell survival when determining whether non‐cell autonomous processes contribute to pathology.

Recent studies of human iPSC‐derived MNs from our group and others have demonstrated physiological changes in MNs harboring ALS‐related mutations (Devlin et al., 2015; Guo et al., 2017; Naujock et al., 2016; Sareen et al., 2013; Wainger et al., 2014; Z. Zhang et al., 2013). The most commonly reported physiological change in ALS‐affected MNs is a reduction in output, or hypoexcitability similar to that revealed in the present study (Devlin et al., 2015; Guo et al., 2017; Naujock et al., 2016; Sareen et al., 2013; Z. Zhang et al., 2013). Such perturbations in the function of iPSC‐derived MNs were previously assumed to reflect cell autonomous disease mechanisms in cultures consisting of approximately 80% neurons, 50% of which were MNs (Devlin et al., 2015). However, in the present study we found that patient iPSC‐derived astrocytes caused a reduction in the functional output of control and patient iPSC‐derived MNs, supporting non‐cell autonomous mechanisms. Our findings therefore implicate “contaminant” astrocytes present in previous studies of enriched motor neuron mixed cultures as key mediators of MN dysfunction. Interestingly, a previous rodent‐based study has also shown non‐cell autonomous effects of astrocytes on the electrophysiological properties of control MNs (Fritz et al., 2013). Fritz and colleagues showed that astrocyte conditioned medium, taken from primary cultures of mutant *SOD1* expressing mouse astrocytes, induced changes in the output of wild‐type mouse MNs, thus implicating toxic factors released by astrocytes as mediators of altered MN function (Yamanaka & Komine, 2018). Factors released by astrocytes which may alter MN function include the effectors of necroptosis: receptor‐integrating serine/threonine‐protein kinase 1 (RIP1) and mixed lineage kinase domain‐like (MLKL) (Re et al., 2014), proinflammatory cytokines and inflammatory mediators (Aebischer et al., 2011; Kia et al., 2018; Phatnani et al., 2013; Tripathi et al., 2017) as well as reactive oxygen species (ROS) (Marchetto et al., 2008; Rao & Weiss, 2004).

The toxic effects of the astrocyte secretome have also been demonstrated in the field of C9orf72‐mediated ALS, although this area remains grossly unexplored. Madill and colleagues showed that C9‐ALS patient iPSC‐derived astrocytes modulate the autophagy pathway in a non‐cell autonomous manner (Madill et al., 2017). Cells treated with patient conditioned medium demonstrated decreased expression of LC3‐II, a key adapter autophagy protein, with a concomitant accumulation of p62 and increased SOD1 expression. Additionally, micro‐RNAs secreted through astrocyte‐derived extracellular vesicles cause increased neuronal death and deficits in neurite outgrowth in control mouse MNs (Varcianna et al., 2019). Through pathway analysis, they identified that hsa‐miR‐494‐3p regulates axonal maintenance, with its primary target being Semaphorin 3A (SEM3A). Treatment of the MNs with a miR‐494‐3p mimic in the presence of C9 iAstrocyte conditioned medium significantly reduced the levels of SEM3A by 25% in the MNs, and increased branching and neurite length, and survival.

An additional hypothesis to explain the toxic effect of ALS astrocytes on MN function is the loss or reduction of normal supportive roles fulfilled by astrocytes, including homeostatic regulation of extracellular glutamate (Foran & Trotti, 2009; Sasabe et al., 2012). This hypothesis is supported by a recent study that showed a reduction in the ability of *VCP* mutant astrocytes to support MN survival (Hall et al., 2017).

RNA‐Seq analysis carried out on *C9orf72* astrocytes in this study further highlighted alterations in multiple new gene pathways which may be causative towards both cell autonomous and non‐cell autonomous pathophysiology. We observed upregulation of many genes involved in ribosome biogenesis and assembly. This is of interest in view of recent interactome studies and yeast genetic modifier screens that show toxic di‐peptide repeat proteins play a role in ribosomal processing/biogenesis and reduce overall cell translation (Chai & Gitler, 2018; Hartmann et al., 2018). These studies thus provide indirect support for astrocyte DPRs having a role in the observed dysregulation of ribosomal processing genes. Na^+^/K^+^ ATPase is a membrane bound pump that exchanges Na^+^ and K^+^ across the plasma membrane to maintain ionic concentration gradients, whilst also modulating neuronal excitability in an activity dependent manner (Picton, Nascimento, Broadhead, Sillar, & Miles, 2017). In our transcriptome analysis we observed upregulation of glial specific Na^+^/K^+^ ATPase (ATP1B2). ATP1B2 knock‐out mice exhibit deficits in motor co‐ordination and develop tremors leading to premature death due to osmotic imbalance (Magyar et al., 1994). Furthermore, ATP1A2 is found to be upregulated in astrocytes expressing mutant SOD1 and contributes to non‐cell autonomous toxicity to motor neurons (Gallardo et al., 2014). Furthermore, astrocytic focal adhesion molecules have been implicated in modulating neuronal excitability in seizure paradigms (Cho, Muthukumar, Stork, Coutinho‐Budd, & Freeman, 2018) and dysregulation of cell‐adhesion by L1CAM deficiency leads to impairment of action potential initiation (Valente et al., 2016). Taken together, the transcriptomic data are consistent with the possibility that impairments in astrocytic cellular processes could lead to a Na^+^/K^+^ ionic imbalance in the synaptic cleft, leading to pathological changes in neuronal excitability. Given the complex and dynamic interplay between astrocytes and MNs, it is likely that mechanisms underlying non‐cell autonomous dysfunction and neurodegeneration are varied and reflect an imbalance between loss of homeostatic function and gain of toxic effects. It will therefore be important in future studies to comprehensively evaluate these newly discovered gene pathways and determine the consequences of perturbations in the transcriptome and proteome of astrocytes expressing the human *C9orf72* mutation in order to fully define the mechanism(s) that underlie the observed pathological consequences of human *C9orf72* astrocytes on human MN function.

In summary, our study provides the first report of the direct molecular and cellular impact of the *C9orf72* mutation on human astrocytes and their interaction with human MNs. Findings here demonstrate that astrocytes, in addition to MNs, are affected by expression of mutant *C9orf72*, which leads to the development of pathological changes. In addition, expression of mutant *C9orf72* in astrocytes induces progressive dysfunction of MNs due to the loss of voltage‐activated currents. These data suggest that non‐cell autonomous disease mechanisms are a contributor to *C9orf72*‐mediated ALS. Furthermore, our study demonstrates the value of combining gene‐editing with sensitive physiological studies in human iPSC‐based neurodegenerative disease modeling.

## CONFLICT OF INTEREST

The authors declare no potential conflict of interest.

## AUTHOR CONTRIBUTIONS

C.Z, A.C.D., A.K.C., S.C., and G.B.M. conceived and designed the experiments. C.Z, A.C.D, A.K.C, M.S, B.T.S, and V.B performed experiments and analyzed the data. K.B. maintained iPSC lines. K.B. and A.R.M differentiated and validated iPSC lines. C.E.S generated and provided iPSC lines. C.Z, A.C.D, A.K.C, G.B.M, and S.C. interpreted the data and wrote the manuscript. C.Z, A.C.D., A.K.C, M.S, B.T.S, G.B.M., and S.C. and edited and revised the manuscript.

## Supporting information


**Figure S1** Validation of iPSCs
(a) Representative immunocytochemistry showed iPSCs positive for pluripotent markers NANOG, SOX2, OCT3/4 and TRA‐1‐60. (Scale bars: 50 μm)
(b) Representative immunocytochemistry showed iPSC‐derived cells positive for markers of three germ layers following differentiation: neuroectroderm (SOX1 and NESTIN), mesoderm (BRACHYURY and EOMES) and endoderm (FOXA2 and GATA‐4). (Scale bars: 50 μm)
(c) Representative repeat‐primed PCR results showed the G_4_C_2_ repeat expansion present in C9 lines but absent in control or the C9‐Δ line.

**Figure S2** Validation of RNA FISH
Representative RNA FISH images of RNase & DNase treatment with a probe against *C9orf72* repeat expansion and with a probe against the DM2 repeat expansion, respectively.

**Figure S3** Mutant and gene‐edited *C9orf72* iPSC‐derived astrocytes have similar levels of the *C9orf72* protein
(a) A representative western blot showing *C9orf72* protein level isolated from iPSC‐derived astrocytes. GAPDH was used as a loading control.
(b) Quantification of relative protein levels of C9ORF72 showed no change between gene edited (C9‐Δ) and mutant astrocyte (C9‐3) or between Ctrl‐2 and C9‐1, C9‐2, C9‐3 astrocytes when compared with loading controls GAPDH (ns, not significant; Student's *t*‐test).

**Figure S4** TDP‐43 proteinopathies were not observed in patient iPSC‐derived astrocytes
(a) Representative images of TDP‐43 immunostaining in astrocytes derived from a control, a C9 and the C9‐Δ iPSC lines. TDP‐43 showed predominant nuclear staining in the cytoplasm. No apparent aggregates or inclusions of TDP‐43 were detected in mutant astrocytes. (Scale bars: 10 μm)
(b‐c) Densitometric analysis of cytoplasmic (b) and nuclear (c) TDP‐43 showed no change between control and C9 astrocytes or between C9‐3 and C9‐Δ astrocytes (ns, not significant; Student's *t*‐test).
(d) A representative western blot of TDP‐43 in the soluble protein fraction isolated from iPSC‐derived astrocytes. GAPDH was used as a loading control.
(e) Quantification of relative protein levels of soluble TDP‐43 showed no change between control and mutant astrocytes or between C9‐3 and C9‐Δ astrocytes compared with loading controls GAPDH (ns, not significant; Student's *t*‐test).

**Figure S5** Physiological properties of MNs derived from individual iPSC lines
(a) Firing properties of the control iPSC‐derived MNs co‐cultured with each of the iPSC‐derived astrocytes lines utilised: 1 control line (n = 87), 3 *C9ORF72* (C9‐1, n = 77; C9‐2, n = 81, C9‐3, n = 103) lines and 1 gene‐edited C9‐Δ (n = 155) astrocyte lines. (b) Peak Na^+^ currents and (c) peak K^+^ currents of control MNs co‐cultured with each iPSC line (Control, n = 93; C9‐1, n = 79; C9‐2, n = 82, C9‐3, n = 105; C9‐Δ, n = 156) from 3–10 weeks post‐plating respectively.

**Figure S6** Current–voltage relationships of Na^+^ and K^+^ currents
(a‐b) Current–voltage relationships of Na^+^ currents recorded from control iPSC‐derived MNs on astrocytes derived from various iPSC lines (Control, n = 93; C9‐1, n = 79; C9‐2, n = 82, C9‐3, n = 105; C9‐Δ, n = 156) from 3–10 weeks post‐plating respectively.
(c‐d) Current–voltage relationships of K^+^ currents recorded from control iPSC‐derived MNs on astrocytes derived from various iPSC lines (Control, n = 93; C9‐1, n = 79; C9‐2, n = 82, C9‐3, n = 105; C9‐Δ, n = 156) from 3–10 weeks post‐plating respectively.

**Figure S7** Current–voltage relationships of Na^+^ and K^+^ currents
(a) Current–voltage relationships of Na^+^ currents recorded at weeks 7–12 weeks post‐plating from mutant and gene‐edited iPSC‐derived MNs in MN‐enriched cultures. (C9‐1, n = 48; C9‐3, n = 62; C9‐Δ1, n = 17; C9‐Δ3, n = 65)
(b) Current–voltage relationships of K^+^ currents recorded at weeks 7–12 weeks post‐plating from mutant and gene‐edited iPSC‐derived MNs in MN‐enriched cultures. (C9‐1, n = 48; C9‐3, n = 62; C9‐Δ1, n = 17; C9‐Δ3, n = 65)
(c) Current–voltage relationships of Na^+^ currents recorded from mutant and gene‐edited iPSC‐derived MNs co‐cultured with mutant and gene‐edited astrocytes respectively at weeks 7–12. (C9‐2, n = 31; C9‐3, n = 47; C9‐Δ2, n = 27; C9‐Δ3, n = 37)
(d) Current–voltage relationships of K^+^ currents recorded from mutant and gene‐edited iPSC‐derived MNs co‐cultured with mutant and gene‐edited astrocytes respectively at weeks 7–12. (C9‐2, n = 31; C9‐3, n = 47; C9‐Δ2, n = 27; C9‐Δ3, n = 37)

**Figure S8** List of genes that are significantly upregulated in C9ORF72 mutant astrocytes (FDR 0.1)

**Figure S9** List of genes that are significantly downregulated in C9ORF72 mutant astrocytes (FDR 0.1)

## Data Availability

The data that support the findings of this study are available from the corresponding author upon reasonable request.
