# Supplementary material for: Mutant C9orf72 human iPSC‐derived astrocytes cause non‐cell autonomous motor neuron pathophysiology
Source: Glia. 2019 Dec 16;68(5):1046–64. doi: 10.1002/glia.23761 (PMC7078830; doi:10.1002/glia.23761)
Supplement: Supplementary file 1 — Figure S1 Validation of iPSCs (a) Representative immunocytochemistry showed iPSCs positive for pluripotent markers NANOG, SOX2, OCT3/4 and TRA‐1‐60. (Scale bars: 50 μm) (b) Representative immunocytochemistry showed iPSC‐derived cells positive for markers of three germ layers following differentiation: neuroectroderm (SOX1 and NESTIN), mesoderm (BRACHYURY and EOMES) and endoderm (FOXA2 and GATA‐4). (Scale bars: 50 μm) (c) Representative repeat‐primed PCR results showed the G4C2 repeat expansion present in C9 lines but absent in control or the C9‐Δ line. [file GLIA-68-1046-s001.docx]

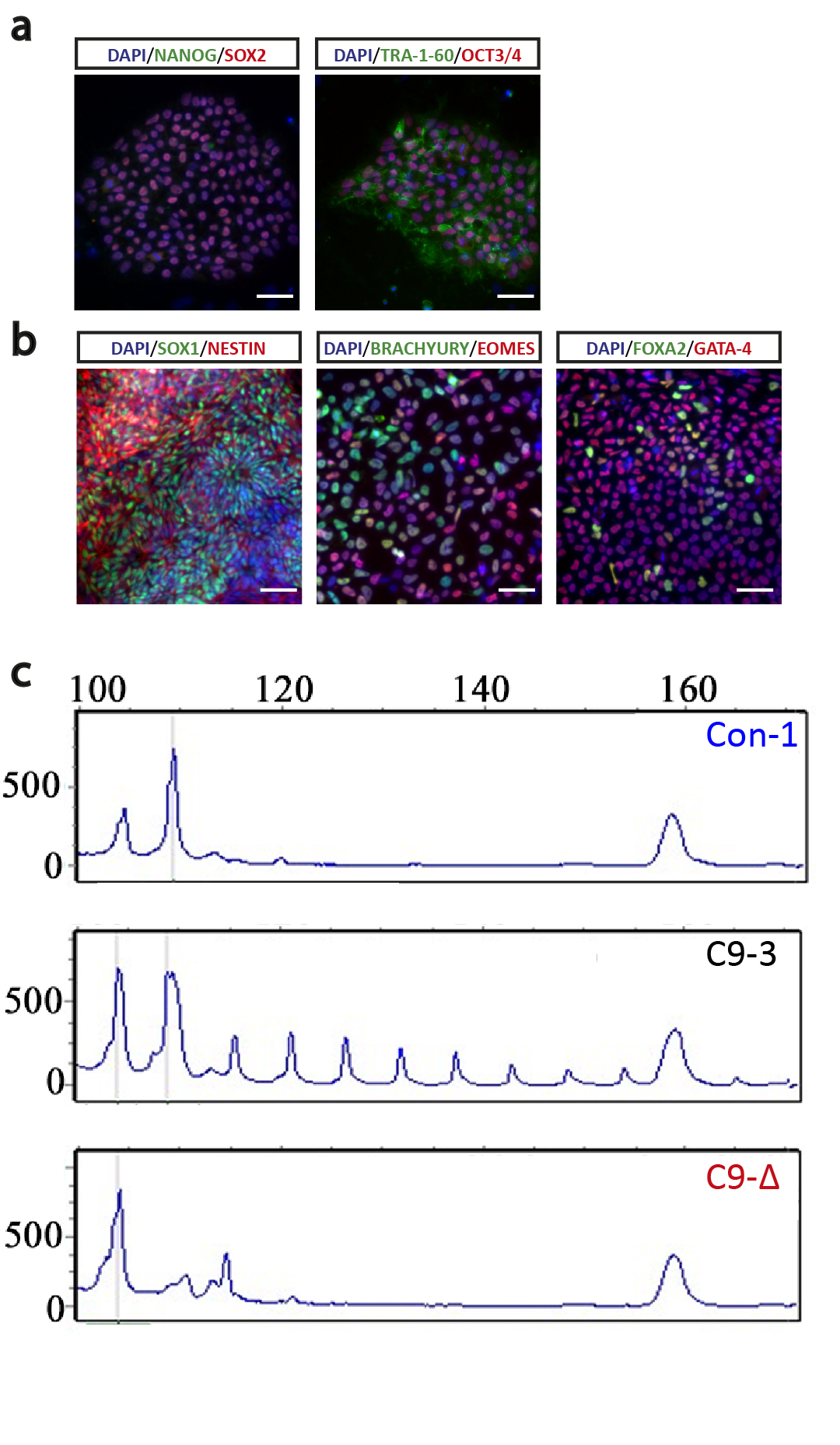


**Supplementary Figure 1. Validation of iPSCs**

(a) Representative immunocytochemistry showed iPSCs positive for pluripotent markers NANOG, SOX2, OCT3/4 and TRA-1-60. (Scale bars: 50 µm)

(b) Representative immunocytochemistry showed iPSC-derived cells positive for markers of three germ layers following differentiation: neuroectroderm (SOX1 and NESTIN), mesoderm (BRACHYURY and EOMES) and endoderm (FOXA2 and GATA-4). (Scale bars: 50 µm)

(c) Representative repeat-primed PCR results showed the G_4_C_2_ repeat expansion present in C9 lines but absent in control or the C9-Δ line.
