# Supplementary material for: Mutant C9orf72 human iPSC‐derived astrocytes cause non‐cell autonomous motor neuron pathophysiology
Source: Glia. 2019 Dec 16;68(5):1046–64. doi: 10.1002/glia.23761 (PMC7078830; doi:10.1002/glia.23761)
Supplement: Supplementary file 2 — Figure S2 Validation of RNA FISH Representative RNA FISH images of RNase & DNase treatment with a probe against C9orf72 repeat expansion and with a probe against the DM2 repeat expansion, respectively. [file GLIA-68-1046-s002.docx]

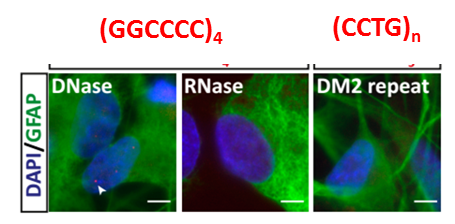


**Supplementary Figure 2. Validation of RNA FISH**

Representative RNA FISH images of RNase & DNase treatment with a probe against *C9orf72* repeat expansion and with a probe against the DM2 repeat expansion, respectively.
