# Supplementary material for: Mutant C9orf72 human iPSC‐derived astrocytes cause non‐cell autonomous motor neuron pathophysiology
Source: Glia. 2019 Dec 16;68(5):1046–64. doi: 10.1002/glia.23761 (PMC7078830; doi:10.1002/glia.23761)
Supplement: Supplementary file 3 — Figure S3 Mutant and gene‐edited C9orf72 iPSC‐derived astrocytes have similar levels of the C9orf72 protein (a) A representative western blot showing C9orf72 protein level isolated from iPSC‐derived astrocytes. GAPDH was used as a loading control. (b) Quantification of relative protein levels of C9ORF72 showed no change between gene edited (C9‐Δ) and mutant astrocyte (C9‐3) or between Ctrl‐2 and C9‐1, C9‐2, C9‐3 astrocytes when compared with loading controls GAPDH (ns, not significant; Student's t‐test). [file GLIA-68-1046-s003.docx]

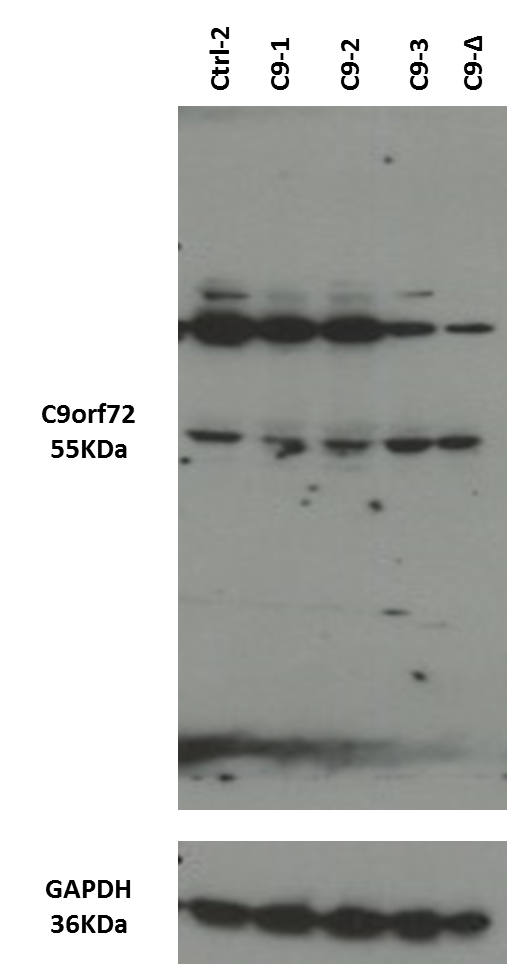


**a**


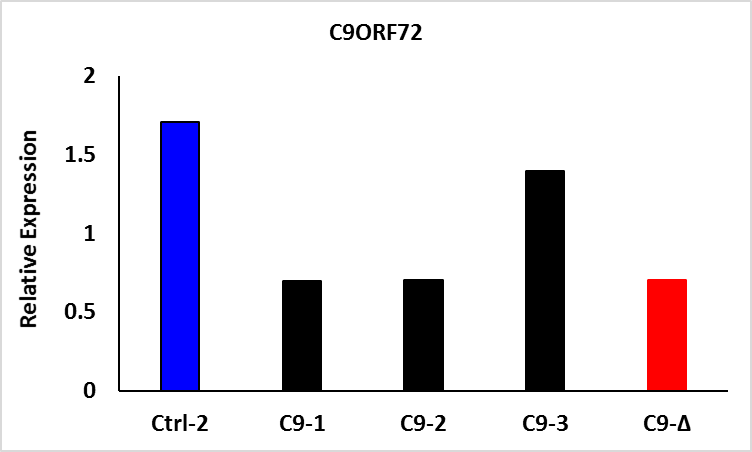


**b**

**Supplementary Figure 3. Mutant and gene-edited *C9orf72* iPSC-derived astrocytes have similar levels of the *C9orf72* protein**

1. A representative western blot showing *C9orf72* protein level isolated from iPSC-derived astrocytes. GAPDH was used as a loading control.
2. Quantification of relative protein levels of C9ORF72 showed no change between gene edited (C9-Δ) and mutant astrocyte (C9-3) or between Ctrl-2 and C9-1, C9-2, C9-3 astrocytes when compared with loading controls GAPDH (ns, not significant; Student’s t-test).
