# Supplementary material for: Mutant C9orf72 human iPSC‐derived astrocytes cause non‐cell autonomous motor neuron pathophysiology
Source: Glia. 2019 Dec 16;68(5):1046–64. doi: 10.1002/glia.23761 (PMC7078830; doi:10.1002/glia.23761)
Supplement: Supplementary file 4 — Figure S4 TDP‐43 proteinopathies were not observed in patient iPSC‐derived astrocytes (a) Representative images of TDP‐43 immunostaining in astrocytes derived from a control, a C9 and the C9‐Δ iPSC lines. TDP‐43 showed predominant nuclear staining in the cytoplasm. No apparent aggregates or inclusions of TDP‐43 were detected in mutant astrocytes. (Scale bars: 10 μm) (b‐c) Densitometric analysis of cytoplasmic (b) and nuclear (c) TDP‐43 showed no change between control and C9 astrocytes or between C9‐3 and C9‐Δ astrocytes (ns, not significant; Student's t‐test). (d) A representative western blot of TDP‐43 in the soluble protein fraction isolated from iPSC‐derived astrocytes. GAPDH was used as a loading control. (e) Quantification of relative protein levels of soluble TDP‐43 showed no change between control and mutant astrocytes or between C9‐3 and C9‐Δ astrocytes compared with loading controls GAPDH (ns, not significant; Student's t‐test). [file GLIA-68-1046-s004.docx]

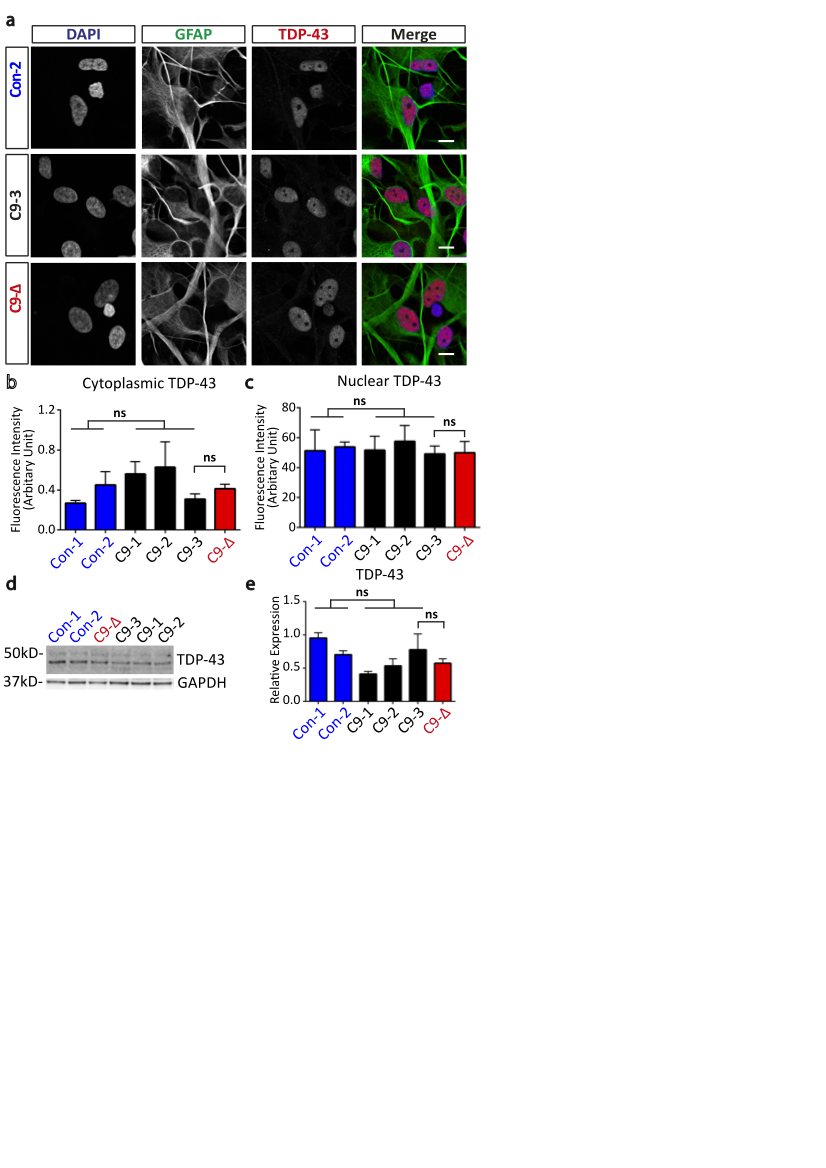


**Supplementary Figure 4. TDP-43 proteinopathies were not observed in patient iPSC-derived astrocytes**

(a) Representative images of TDP-43 immunostaining in astrocytes derived from a control, a C9 and the C9-Δ iPSC lines. TDP-43 showed predominant nuclear staining in the cytoplasm. No apparent aggregates or inclusions of TDP-43 were detected in mutant astrocytes. (Scale bars: 10 µm)

(b-c) Densitometric analysis of cytoplasmic (b) and nuclear (c) TDP-43 showed no change between control and C9 astrocytes or between C9-3 and C9-Δ astrocytes (ns, not significant; Student’s t-test).

(d) A representative western blot of TDP-43 in the soluble protein fraction isolated from iPSC-derived astrocytes. GAPDH was used as a loading control.

(e) Quantification of relative protein levels of soluble TDP-43 showed no change between control and mutant astrocytes or between C9-3 and C9-Δ astrocytes compared with loading controls GAPDH (ns, not significant; Student’s t-test).
