# Supplementary material for: Mutant C9orf72 human iPSC‐derived astrocytes cause non‐cell autonomous motor neuron pathophysiology
Source: Glia. 2019 Dec 16;68(5):1046–64. doi: 10.1002/glia.23761 (PMC7078830; doi:10.1002/glia.23761)
Supplement: Supplementary file 5 — Figure S5 Physiological properties of MNs derived from individual iPSC lines (a) Firing properties of the control iPSC‐derived MNs co‐cultured with each of the iPSC‐derived astrocytes lines utilised: 1 control line (n = 87), 3 C9ORF72 (C9‐1, n = 77; C9‐2, n = 81, C9‐3, n = 103) lines and 1 gene‐edited C9‐Δ (n = 155) astrocyte lines. (b) Peak Na+ currents and (c) peak K+ currents of control MNs co‐cultured with each iPSC line (Control, n = 93; C9‐1, n = 79; C9‐2, n = 82, C9‐3, n = 105; C9‐Δ, n = 156) from 3–10 weeks post‐plating respectively. [file GLIA-68-1046-s005.docx]

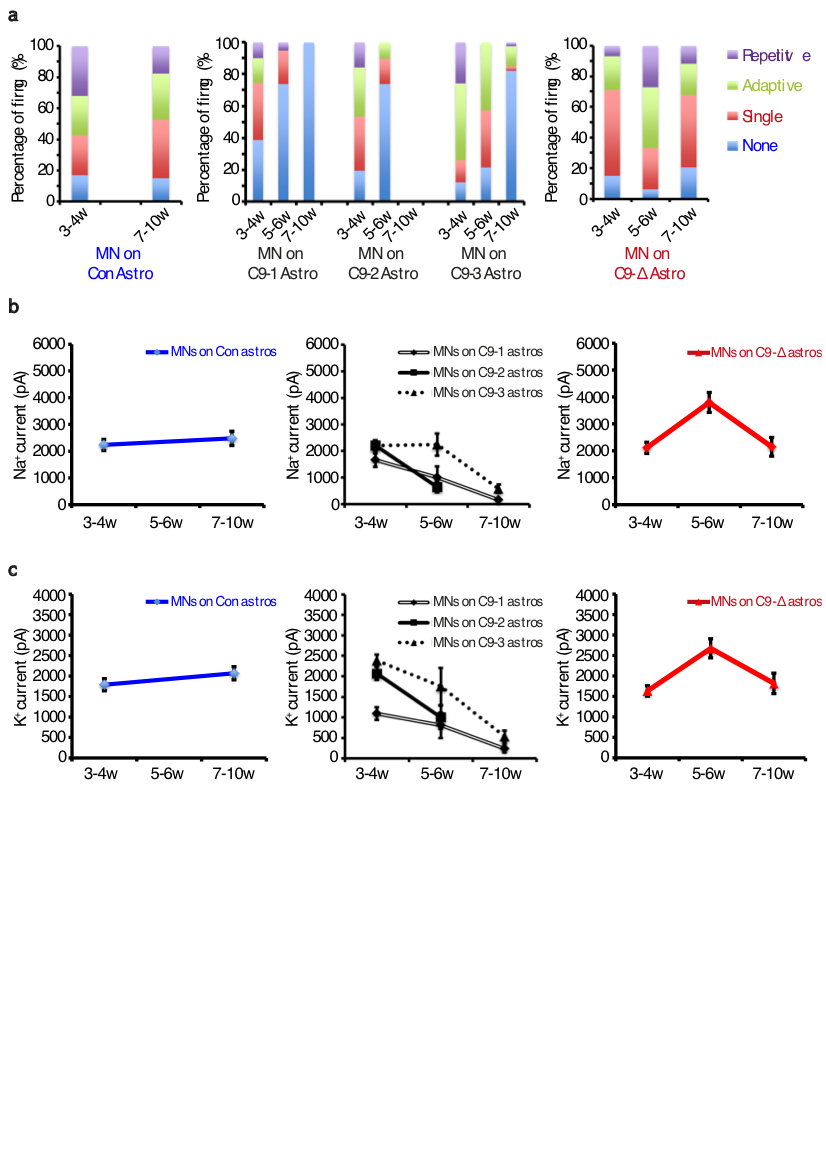


**Supplementary Figure 5. Physiological properties of MNs derived from individual iPSC lines**

(a) Firing properties of the control iPSC-derived MNs co-cultured with each of the iPSC-derived astrocytes lines utilised: 1 control line (n=87), 3 *C9ORF72* (C9-1, n=77; C9-2, n=81, C9-3, n=103) lines and 1 gene-edited C9-Δ (n=155) astrocyte lines. (b) Peak Na^+^ currents and (c) peak K^+^ currents of control MNs co-cultured with each iPSC line (Control, n=93; C9-1, n=79; C9-2, n=82, C9-3, n=105; C9-Δ, n=156) from 3- 10 weeks post-plating respectively.
