# Supplementary material for: Mutant C9orf72 human iPSC‐derived astrocytes cause non‐cell autonomous motor neuron pathophysiology
Source: Glia. 2019 Dec 16;68(5):1046–64. doi: 10.1002/glia.23761 (PMC7078830; doi:10.1002/glia.23761)
Supplement: Supplementary file 6 — Figure S6 Current–voltage relationships of Na+ and K+ currents (a‐b) Current–voltage relationships of Na+ currents recorded from control iPSC‐derived MNs on astrocytes derived from various iPSC lines (Control, n = 93; C9‐1, n = 79; C9‐2, n = 82, C9‐3, n = 105; C9‐Δ, n = 156) from 3–10 weeks post‐plating respectively. (c‐d) Current–voltage relationships of K+ currents recorded from control iPSC‐derived MNs on astrocytes derived from various iPSC lines (Control, n = 93; C9‐1, n = 79; C9‐2, n = 82, C9‐3, n = 105; C9‐Δ, n = 156) from 3–10 weeks post‐plating respectively. [file GLIA-68-1046-s006.docx]

**
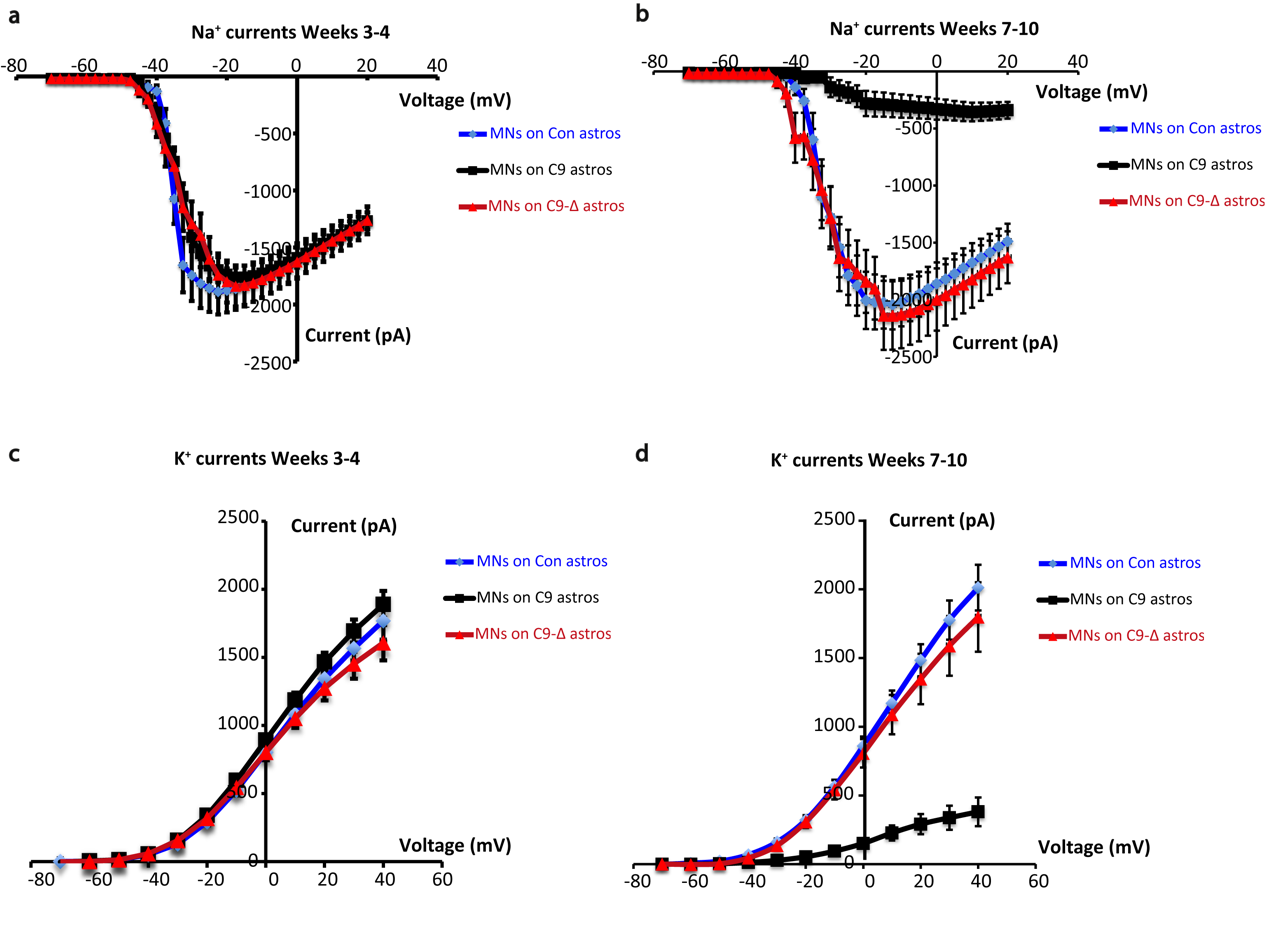
**

**Supplementary Figure 6. Current–voltage relationships of Na^+^ and K^+^ currents**

(a-b) Current–voltage relationships of Na^+^ currents recorded from control iPSC-derived MNs on astrocytes derived from various iPSC lines (Control, n=93; C9-1, n=79; C9-2, n=82, C9-3, n=105; C9-Δ, n=156) from 3- 10 weeks post-plating respectively.

(c-d) Current–voltage relationships of K^+^ currents recorded from control iPSC-derived MNs on astrocytes derived from various iPSC lines (Control, n=93; C9-1, n=79; C9-2, n=82, C9-3, n=105; C9-Δ, n=156) from 3- 10 weeks post-plating respectively.
