# Supplementary material for: Mutant C9orf72 human iPSC‐derived astrocytes cause non‐cell autonomous motor neuron pathophysiology
Source: Glia. 2019 Dec 16;68(5):1046–64. doi: 10.1002/glia.23761 (PMC7078830; doi:10.1002/glia.23761)
Supplement: Supplementary file 7 — Figure S7 Current–voltage relationships of Na+ and K+ currents (a) Current–voltage relationships of Na+ currents recorded at weeks 7–12 weeks post‐plating from mutant and gene‐edited iPSC‐derived MNs in MN‐enriched cultures. (C9‐1, n = 48; C9‐3, n = 62; C9‐Δ1, n = 17; C9‐Δ3, n = 65) (b) Current–voltage relationships of K+ currents recorded at weeks 7–12 weeks post‐plating from mutant and gene‐edited iPSC‐derived MNs in MN‐enriched cultures. (C9‐1, n = 48; C9‐3, n = 62; C9‐Δ1, n = 17; C9‐Δ3, n = 65) (c) Current–voltage relationships of Na+ currents recorded from mutant and gene‐edited iPSC‐derived MNs co‐cultured with mutant and gene‐edited astrocytes respectively at weeks 7–12. (C9‐2, n = 31; C9‐3, n = 47; C9‐Δ2, n = 27; C9‐Δ3, n = 37) (d) Current–voltage relationships of K+ currents recorded from mutant and gene‐edited iPSC‐derived MNs co‐cultured with mutant and gene‐edited astrocytes respectively at weeks 7–12. (C9‐2, n = 31; C9‐3, n = 47; C9‐Δ2, n = 27; C9‐Δ3, n = 37) [file GLIA-68-1046-s007.docx]

**
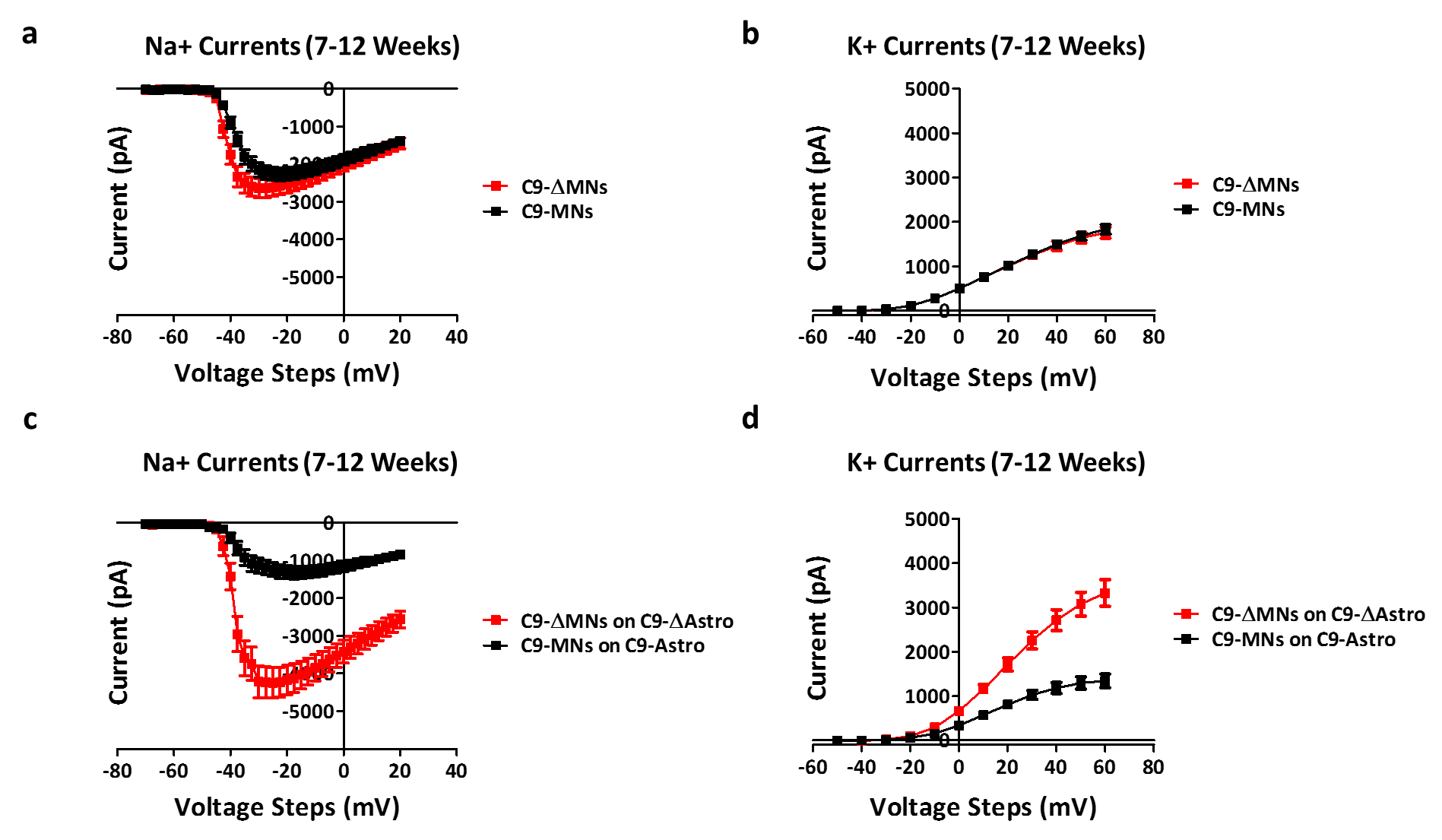
**

**Supplementary Figure 7. Current–voltage relationships of Na^+^ and K^+^ currents**

(a) Current–voltage relationships of Na^+^ currents recorded at weeks 7-12 weeks post-plating from mutant and gene-edited iPSC-derived MNs in MN-enriched cultures. (C9-1, n=48; C9-3, n=62; C9-Δ1, n=17; C9-Δ3, n=65)

(b) Current–voltage relationships of K^+^ currents recorded at weeks 7-12 weeks post-plating from mutant and gene-edited iPSC-derived MNs in MN-enriched cultures. (C9-1, n=48; C9-3, n=62; C9-Δ1, n=17; C9-Δ3, n=65)

(c) Current–voltage relationships of Na^+^ currents recorded from mutant and gene-edited iPSC-derived MNs co-cultured with mutant and gene-edited astrocytes respectively at weeks 7-12. (C9-2, n=31; C9-3, n=47; C9-Δ2, n=27; C9-Δ3, n=37)

(d) Current–voltage relationships of K^+^ currents recorded from mutant and gene-edited iPSC-derived MNs co-cultured with mutant and gene-edited astrocytes respectively at weeks 7-12. (C9-2, n=31; C9-3, n=47; C9-Δ2, n=27; C9-Δ3, n=37)
