# Supplementary material for: Mutant C9orf72 human iPSC‐derived astrocytes cause non‐cell autonomous motor neuron pathophysiology
Source: Glia. 2019 Dec 16;68(5):1046–64. doi: 10.1002/glia.23761 (PMC7078830; doi:10.1002/glia.23761)
Supplement: Supplementary file 8 — Figure S8 List of genes that are significantly upregulated in C9ORF72 mutant astrocytes (FDR 0.1) [file GLIA-68-1046-s008.docx]

**Supplementary Figure 8. List of genes that are significantly upregulated in C9ORF72 mutant astrocytes (FDR 0.1)**

| **Gene** | **Gene Name** | **C9-2** | **C9-Δ2** | **C9-3** | **C9-Δ3** |
| --- | --- | --- | --- | --- | --- |
|  |  |  |  |  |  |
| ENSG00000215190 | LINC00680 | 0.50 | 0.02 | 10.17 | 0.01 |
| ENSG00000267034 | AC010980.2 | 0.30 | 0.01 | 5.23 | 0.01 |
| ENSG00000237732 | AC010980.1 | 0.32 | 0.03 | 3.82 | 0.01 |
| ENSG00000167785 | ZNF558 | 1.77 | 0.02 | 5.64 | 0.04 |
| ENSG00000145506 | NKD2 | 0.25 | 0.06 | 9.40 | 0.08 |
| ENSG00000125730 | C3 | 1.39 | 0.27 | 8.90 | 0.06 |
| ENSG00000104435 | STMN2 | 0.74 | 0.05 | 10.15 | 0.08 |
| ENSG00000211445 | GPX3 | 20.57 | 2.21 | 200.35 | 1.74 |
| ENSG00000130035 | GALNT8 | 1.15 | 0.02 | 3.80 | 0.05 |
| ENSG00000171388 | APLN | 1.83 | 0.18 | 11.98 | 0.16 |
| ENSG00000066248 | NGEF | 4.83 | 0.63 | 11.03 | 0.16 |
| ENSG00000197406 | DIO3 | 1.47 | 0.56 | 6.39 | 0.09 |
| ENSG00000168081 | PNOC | 4.85 | 0.23 | 24.38 | 0.42 |
| ENSG00000158270 | COLEC12 | 3.26 | 0.11 | 32.73 | 0.60 |
| ENSG00000186642 | PDE2A | 2.83 | 0.76 | 4.00 | 0.06 |
| ENSG00000164651 | SP8 | 2.34 | 0.06 | 4.64 | 0.11 |
| ENSG00000204556 | AL450124.1 | 4.21 | 0.38 | 2.70 | 0.03 |
| ENSG00000176956 | LY6H | 0.73 | 0.26 | 7.50 | 0.23 |
| ENSG00000135333 | EPHA7 | 3.17 | 0.03 | 27.07 | 0.77 |
| ENSG00000162804 | SNED1 | 0.13 | 0.05 | 5.08 | 0.18 |
| ENSG00000168743 | NPNT | 1.17 | 0.45 | 95.19 | 3.44 |
| ENSG00000262655 | SPON1 | 0.42 | 0.10 | 8.12 | 0.27 |
| ENSG00000105996 | HOXA2 | 6.18 | 0.01 | 7.85 | 0.22 |
| ENSG00000143502 | SUSD4 | 1.01 | 0.03 | 7.16 | 0.37 |
| ENSG00000154928 | EPHB1 | 18.98 | 0.35 | 13.50 | 0.72 |
| ENSG00000253552 | HOXA-AS2 | 3.51 | 0.01 | 5.88 | 0.23 |
| ENSG00000125084 | WNT1 | 0.86 | 0.08 | 4.10 | 0.19 |
| ENSG00000119147 | C2orf40 | 2.86 | 0.10 | 1.71 | 0.07 |
| ENSG00000114541 | FRMD4B | 5.27 | 0.85 | 21.55 | 1.28 |
| ENSG00000171246 | NPTX1 | 0.60 | 0.19 | 4.04 | 0.25 |
| ENSG00000197576 | HOXA4 | 1.20 | 0.02 | 4.80 | 0.27 |
| ENSG00000185052 | SLC24A3 | 1.34 | 0.03 | 2.82 | 0.19 |
| ENSG00000163032 | VSNL1 | 0.41 | 0.02 | 9.87 | 0.70 |
| ENSG00000229891 | LINC01315 | 6.13 | 2.76 | 17.54 | 1.22 |
| ENSG00000181218 | HIST3H2A | 12.86 | 9.14 | 32.39 | 2.12 |
| ENSG00000196954 | CASP4 | 0.20 | 0.02 | 8.31 | 0.62 |
| ENSG00000183570 | PCBP3 | 4.40 | 0.35 | 44.04 | 3.16 |
| ENSG00000152578 | GRIA4 | 0.39 | 0.02 | 25.36 | 2.64 |
| ENSG00000063015 | SEZ6 | 12.70 | 1.70 | 66.02 | 5.34 |
| ENSG00000100095 | SEZ6L | 0.85 | 0.04 | 9.52 | 0.81 |
| ENSG00000076864 | RAP1GAP | 5.77 | 2.14 | 35.67 | 3.75 |
| ENSG00000154783 | FGD5 | 0.16 | 0.00 | 4.76 | 0.29 |
| ENSG00000075043 | KCNQ2 | 0.82 | 0.08 | 10.51 | 1.20 |
| ENSG00000123689 | G0S2 | 28.50 | 6.72 | 22.73 | 1.59 |
| ENSG00000250722 | SELENOP | 4.74 | 0.80 | 20.00 | 2.27 |
| ENSG00000138944 | KIAA1644 | 10.26 | 0.53 | 8.10 | 0.90 |
| ENSG00000142494 | SLC47A1 | 4.18 | 0.92 | 7.79 | 0.83 |
| ENSG00000149571 | KIRREL3 | 2.24 | 0.31 | 8.64 | 0.84 |
| ENSG00000169255 | B3GALNT1 | 1.11 | 0.01 | 2.82 | 0.22 |
| ENSG00000176907 | C8orf4 | 4.27 | 0.91 | 26.74 | 2.31 |
| ENSG00000182742 | HOXB4 | 3.97 | 0.77 | 7.34 | 0.70 |
| ENSG00000164440 | TXLNB | 3.83 | 1.30 | 0.34 | 0.03 |
| ENSG00000105976 | MET | 3.01 | 1.06 | 16.67 | 1.80 |
| ENSG00000143416 | SELENBP1 | 6.78 | 2.72 | 34.10 | 4.06 |
| ENSG00000225472 | AL136366.1 | 3.67 | 1.03 | 5.93 | 0.65 |
| ENSG00000125869 | LAMP5 | 12.59 | 9.66 | 44.23 | 3.85 |
| ENSG00000154856 | APCDD1 | 12.61 | 0.89 | 8.30 | 1.14 |
| ENSG00000107317 | PTGDS | 25.96 | 6.71 | 160.22 | 19.04 |
| ENSG00000121966 | CXCR4 | 42.00 | 13.44 | 91.78 | 11.59 |
| ENSG00000226752 | PSMD5-AS1 | 12.14 | 5.45 | 1.16 | 0.14 |
| ENSG00000183036 | PCP4 | 8.48 | 1.56 | 9.37 | 1.28 |
| ENSG00000007516 | BAIAP3 | 12.41 | 0.75 | 2.27 | 0.33 |
| ENSG00000147408 | CSGALNACT1 | 5.54 | 2.59 | 12.47 | 2.22 |
| ENSG00000213853 | EMP2 | 6.84 | 1.56 | 19.78 | 2.97 |
| ENSG00000049540 | ELN | 189.26 | 3.21 | 804.43 | 120.57 |
| ENSG00000204389 | HSPA1A | 1.71 | 0.42 | 6.53 | 1.00 |
| ENSG00000118898 | PPL | 2.52 | 1.09 | 1.20 | 0.17 |
| ENSG00000249395 | CASC9 | 5.86 | 0.09 | 1.24 | 0.18 |
| ENSG00000007944 | MYLIP | 1.99 | 0.47 | 19.52 | 2.87 |
| ENSG00000168899 | VAMP5 | 22.79 | 1.12 | 16.78 | 2.61 |
| ENSG00000067715 | SYT1 | 6.33 | 1.00 | 14.15 | 2.13 |
| ENSG00000047457 | CP | 104.23 | 0.24 | 3.31 | 0.61 |
| ENSG00000100077 | GRK3 | 0.81 | 0.37 | 2.93 | 0.44 |
| ENSG00000154102 | C16orf74 | 7.86 | 1.93 | 7.77 | 1.16 |
| ENSG00000112378 | PERP | 6.34 | 1.20 | 17.61 | 2.95 |
| ENSG00000120093 | HOXB3 | 8.49 | 1.66 | 15.14 | 2.18 |
| ENSG00000123500 | COL10A1 | 0.35 | 0.03 | 4.24 | 0.79 |
| ENSG00000140807 | NKD1 | 2.69 | 0.46 | 6.57 | 1.32 |
| ENSG00000179981 | TSHZ1 | 10.08 | 4.51 | 17.10 | 2.87 |
| ENSG00000188257 | PLA2G2A | 8.12 | 0.07 | 0.36 | 0.04 |
| ENSG00000079257 | LXN | 1.24 | 0.10 | 2.88 | 0.49 |
| ENSG00000171812 | COL8A2 | 37.60 | 1.07 | 90.00 | 16.31 |
| ENSG00000234690 | AC106869.1 | 1.32 | 0.73 | 2.28 | 0.40 |
| ENSG00000104518 | GSDMD | 2.57 | 0.41 | 4.82 | 0.90 |
| ENSG00000115041 | KCNIP3 | 1.22 | 0.66 | 1.93 | 0.33 |
| ENSG00000132481 | TRIM47 | 11.00 | 6.29 | 33.34 | 5.89 |
| ENSG00000235859 | AC006978.1 | 3.47 | 2.18 | 7.23 | 1.44 |
| ENSG00000122824 | NUDT10 | 5.16 | 0.68 | 4.41 | 0.84 |
| ENSG00000237928 | NFIA-AS2 | 1.82 | 0.48 | 2.92 | 0.61 |
| ENSG00000178814 | OPLAH | 0.57 | 0.08 | 7.96 | 1.54 |
| ENSG00000138678 | GPAT3 | 16.40 | 3.65 | 0.72 | 0.13 |
| ENSG00000166582 | CENPV | 4.04 | 1.52 | 14.81 | 3.13 |
| ENSG00000259610 | AC023034.2 | 1.75 | 0.01 | 2.10 | 0.40 |
| ENSG00000197444 | OGDHL | 1.52 | 1.01 | 6.22 | 1.25 |
| ENSG00000167588 | GPD1 | 6.26 | 1.25 | 2.51 | 0.66 |
| ENSG00000169884 | WNT10B | 3.52 | 1.23 | 6.11 | 1.14 |
| ENSG00000172935 | MRGPRF | 1.17 | 0.19 | 2.59 | 0.57 |
| ENSG00000148735 | PLEKHS1 | 20.13 | 0.05 | 0.10 | 0.02 |
| ENSG00000103316 | CRYM | 2.04 | 0.04 | 18.54 | 4.03 |
| ENSG00000128833 | MYO5C | 8.24 | 4.37 | 3.33 | 0.75 |
| ENSG00000103196 | CRISPLD2 | 3.52 | 0.81 | 4.14 | 0.86 |
| ENSG00000171189 | GRIK1 | 5.48 | 0.23 | 1.84 | 0.53 |
| ENSG00000226742 | HSBP1L1 | 2.25 | 0.27 | 2.85 | 0.60 |
| ENSG00000115468 | EFHD1 | 59.43 | 13.27 | 23.68 | 5.04 |
| ENSG00000136099 | PCDH8 | 4.34 | 3.02 | 17.10 | 4.33 |
| ENSG00000188747 | NOXA1 | 5.11 | 2.90 | 8.43 | 1.82 |
| ENSG00000130222 | GADD45G | 13.19 | 2.83 | 10.18 | 2.82 |
| ENSG00000119866 | BCL11A | 1.55 | 0.17 | 3.11 | 0.80 |
| ENSG00000128805 | ARHGAP22 | 2.50 | 0.78 | 3.64 | 0.88 |
| ENSG00000143772 | ITPKB | 33.65 | 18.61 | 27.25 | 6.29 |
| ENSG00000183778 | B3GALT5 | 4.21 | 1.93 | 2.57 | 0.68 |
| ENSG00000235109 | ZSCAN31 | 1.51 | 0.85 | 2.87 | 0.70 |
| ENSG00000176204 | LRRTM4 | 0.15 | 0.02 | 3.92 | 0.82 |
| ENSG00000154027 | AK5 | 3.92 | 0.48 | 1.31 | 0.31 |
| ENSG00000156298 | TSPAN7 | 2.17 | 0.22 | 13.04 | 3.21 |
| ENSG00000179344 | HLA-DQB1 | 16.41 | 2.05 | 0.23 | 0.05 |
| ENSG00000186973 | FAM183A | 3.03 | 0.12 | 5.27 | 1.17 |
| ENSG00000142937 | RPS8 | 401.32 | 302.38 | 1767.04 | 435.72 |
| ENSG00000167526 | RPL13 | 139.25 | 102.05 | 584.27 | 140.14 |
| ENSG00000162551 | ALPL | 1.83 | 0.36 | 6.05 | 1.55 |
| ENSG00000197958 | RPL12 | 170.37 | 134.14 | 823.64 | 201.77 |
| ENSG00000234741 | GAS5 | 56.41 | 44.17 | 265.71 | 65.67 |
| ENSG00000063177 | RPL18 | 88.70 | 64.80 | 330.78 | 81.53 |
| ENSG00000180638 | SLC47A2 | 27.92 | 8.35 | 4.22 | 1.17 |
| ENSG00000135052 | GOLM1 | 262.15 | 173.38 | 320.21 | 86.78 |
| ENSG00000205403 | CFI | 156.36 | 101.61 | 316.42 | 72.16 |
| ENSG00000135905 | DOCK10 | 7.81 | 0.73 | 2.62 | 0.68 |
| ENSG00000231500 | RPS18 | 561.03 | 424.81 | 2832.01 | 713.81 |
| ENSG00000063438 | AHRR | 2.75 | 0.69 | 6.63 | 1.73 |
| ENSG00000151490 | PTPRO | 5.64 | 0.03 | 2.61 | 0.64 |
| ENSG00000122406 | RPL5 | 444.42 | 310.38 | 1555.39 | 403.76 |
| ENSG00000198034 | RPS4X | 402.45 | 322.69 | 1341.83 | 350.01 |
| ENSG00000188846 | RPL14 | 34.83 | 27.48 | 150.75 | 38.33 |
| ENSG00000162244 | RPL29 | 445.87 | 324.75 | 1737.87 | 452.27 |
| ENSG00000105373 | NOP53 | 29.81 | 20.95 | 95.97 | 25.85 |
| ENSG00000204252 | HLA-DOA | 31.76 | 6.25 | 0.53 | 0.15 |
| ENSG00000145423 | SFRP2 | 0.84 | 0.04 | 73.62 | 22.21 |
| ENSG00000170382 | LRRN2 | 27.91 | 2.40 | 21.13 | 5.61 |
| ENSG00000137154 | RPS6 | 516.66 | 343.18 | 2022.47 | 544.93 |
| ENSG00000021645 | NRXN3 | 2.24 | 1.61 | 3.48 | 0.98 |
| ENSG00000148303 | RPL7A | 774.27 | 536.89 | 2549.97 | 690.83 |
| ENSG00000168546 | GFRA2 | 0.20 | 0.03 | 6.93 | 1.88 |
| ENSG00000175084 | DES | 0.85 | 0.24 | 4.64 | 1.77 |
| ENSG00000163710 | PCOLCE2 | 4.70 | 1.97 | 8.51 | 2.21 |
| ENSG00000071082 | RPL31 | 142.12 | 102.43 | 491.15 | 134.65 |
| ENSG00000142541 | RPL13A | 496.50 | 381.63 | 1728.56 | 483.20 |
| ENSG00000197442 | MAP3K5 | 1.23 | 0.07 | 3.08 | 0.73 |
| ENSG00000138326 | RPS24 | 119.35 | 90.86 | 420.11 | 114.85 |
| ENSG00000106069 | CHN2 | 1.81 | 0.93 | 2.85 | 0.84 |
| ENSG00000224389 | C4B | 10.79 | 6.98 | 6.45 | 1.91 |
| ENSG00000137970 | RPL7P9 | 7.02 | 4.79 | 20.29 | 5.60 |
| ENSG00000281508 | CDR1 | 2.38 | 0.11 | 11.73 | 3.23 |
| ENSG00000204628 | RACK1 | 419.05 | 319.38 | 1626.79 | 459.06 |
| ENSG00000151789 | ZNF385D | 2.49 | 0.38 | 1.94 | 0.57 |
| ENSG00000210082 | MT-RNR2 | 3504.97 | 2622.77 | 5345.47 | 1456.66 |
| ENSG00000133048 | CHI3L1 | 56.89 | 9.35 | 5.27 | 1.29 |
| ENSG00000106483 | SFRP4 | 149.95 | 5.15 | 43.84 | 11.74 |
| ENSG00000175928 | LRRN1 | 66.45 | 12.49 | 92.13 | 25.42 |
| ENSG00000283041 | AC008038.1 | 10.44 | 6.82 | 24.29 | 6.91 |
| ENSG00000188931 | CFAP126 | 7.70 | 0.50 | 2.86 | 1.02 |
| ENSG00000145425 | RPS3A | 343.49 | 237.23 | 1160.06 | 334.47 |
| ENSG00000161970 | RPL26 | 75.41 | 53.86 | 268.74 | 77.86 |
| ENSG00000134419 | RPS15A | 28.07 | 19.92 | 95.77 | 27.34 |
| ENSG00000142676 | RPL11 | 420.39 | 312.84 | 1478.28 | 428.16 |
| ENSG00000149273 | RPS3 | 232.51 | 165.73 | 977.35 | 285.61 |
| ENSG00000266865 | AC138207.8 | 1.27 | 0.03 | 3.53 | 1.02 |
| ENSG00000125691 | RPL23 | 125.28 | 89.13 | 435.08 | 125.36 |
| ENSG00000163682 | RPL9 | 213.37 | 163.63 | 550.47 | 158.87 |
| ENSG00000204338 | CYP21A1P | 4.25 | 2.93 | 1.14 | 0.34 |
| ENSG00000121104 | FAM117A | 4.98 | 2.47 | 9.31 | 2.79 |
| ENSG00000244731 | C4A | 13.04 | 8.11 | 3.40 | 1.05 |
| ENSG00000140988 | RPS2 | 718.99 | 553.93 | 2333.45 | 682.35 |
| ENSG00000271369 | AC087783.2 | 1.89 | 0.06 | 17.59 | 5.48 |
| ENSG00000144713 | RPL32 | 167.93 | 130.20 | 632.59 | 184.99 |
| ENSG00000196639 | HRH1 | 6.51 | 3.60 | 7.56 | 2.20 |
| ENSG00000145592 | RPL37 | 49.39 | 36.38 | 179.47 | 52.61 |
| ENSG00000124126 | PREX1 | 21.45 | 5.35 | 11.52 | 3.63 |
| ENSG00000174444 | RPL4 | 369.41 | 276.75 | 1318.54 | 401.27 |
| ENSG00000088992 | TESC | 19.61 | 1.42 | 2.03 | 0.75 |
| ENSG00000162105 | SHANK2 | 4.52 | 0.06 | 0.69 | 0.21 |
| ENSG00000104112 | SCG3 | 4.97 | 1.04 | 2.11 | 0.75 |
| ENSG00000172809 | RPL38 | 58.81 | 45.62 | 195.16 | 57.77 |
| ENSG00000083845 | RPS5 | 168.90 | 133.49 | 542.68 | 166.79 |
| ENSG00000160179 | ABCG1 | 3.36 | 0.03 | 1.67 | 0.50 |
| ENSG00000109475 | RPL34 | 87.52 | 66.60 | 289.70 | 87.18 |
| ENSG00000186468 | RPS23 | 96.33 | 71.94 | 405.74 | 123.95 |
| ENSG00000142534 | RPS11 | 484.29 | 392.11 | 1380.18 | 420.68 |
| ENSG00000196782 | MAML3 | 3.40 | 0.94 | 3.65 | 1.13 |
| ENSG00000130066 | SAT1 | 448.28 | 201.10 | 233.93 | 64.01 |
| ENSG00000178035 | IMPDH2 | 88.52 | 60.40 | 276.95 | 84.46 |
| ENSG00000095261 | PSMD5 | 14.67 | 6.92 | 5.79 | 1.85 |
| ENSG00000099998 | GGT5 | 1.25 | 0.12 | 2.32 | 0.73 |
| ENSG00000157796 | WDR19 | 12.15 | 8.57 | 17.75 | 5.76 |
| ENSG00000105640 | RPL18A | 156.18 | 127.56 | 733.61 | 231.23 |
| ENSG00000180592 | SKIDA1 | 1.70 | 0.86 | 1.90 | 0.65 |
| ENSG00000175061 | LRRC75A-AS1 | 172.72 | 114.96 | 590.10 | 190.59 |
| ENSG00000044524 | EPHA3 | 17.98 | 0.47 | 30.57 | 9.07 |
| ENSG00000075429 | CACNG5 | 0.68 | 0.02 | 2.87 | 0.92 |
| ENSG00000114391 | RPL24 | 133.76 | 105.09 | 483.94 | 153.21 |
| ENSG00000161016 | RPL8 | 476.94 | 383.76 | 1704.74 | 550.77 |
| ENSG00000138411 | HECW2 | 3.37 | 0.62 | 1.67 | 0.55 |
| ENSG00000164587 | RPS14 | 116.98 | 94.57 | 406.61 | 130.73 |
| ENSG00000176438 | SYNE3 | 1.11 | 0.44 | 3.50 | 1.14 |
| ENSG00000100316 | RPL3 | 799.64 | 616.03 | 2270.56 | 744.51 |
| ENSG00000104408 | EIF3E | 105.63 | 70.90 | 286.96 | 95.12 |
| ENSG00000198755 | RPL10A | 355.42 | 287.46 | 1215.81 | 408.45 |
| ENSG00000166682 | TMPRSS5 | 14.15 | 6.44 | 5.45 | 2.48 |
| ENSG00000140022 | STON2 | 13.89 | 5.75 | 3.52 | 1.26 |
| ENSG00000180233 | ZNRF2 | 2.20 | 1.29 | 2.23 | 0.83 |
| ENSG00000179104 | TMTC2 | 14.49 | 10.13 | 28.75 | 10.27 |
| ENSG00000089009 | RPL6 | 339.93 | 247.31 | 980.39 | 332.28 |
| ENSG00000234779 | AL450003.2 | 1.27 | 0.37 | 2.52 | 0.85 |
| ENSG00000101955 | SRPX | 23.72 | 17.57 | 50.38 | 17.14 |
| ENSG00000196562 | SULF2 | 23.14 | 12.03 | 278.62 | 99.01 |
| ENSG00000130287 | NCAN | 12.92 | 1.30 | 39.85 | 13.78 |
| ENSG00000204634 | TBC1D8 | 3.29 | 1.50 | 5.05 | 1.79 |
| ENSG00000166441 | RPL27A | 77.27 | 60.23 | 286.62 | 99.05 |
| ENSG00000147604 | RPL7 | 433.82 | 309.10 | 1219.55 | 432.75 |
| ENSG00000197182 | MIRLET7BHG | 3.14 | 1.53 | 3.83 | 1.39 |
| ENSG00000165730 | STOX1 | 7.18 | 3.60 | 2.55 | 1.03 |
| ENSG00000177108 | ZDHHC22 | 1.33 | 0.20 | 2.20 | 0.95 |
| ENSG00000116251 | RPL22 | 99.57 | 79.59 | 221.92 | 80.54 |
| ENSG00000147403 | RPL10 | 327.09 | 251.90 | 1142.32 | 413.68 |
| ENSG00000198786 | MT-ND5 | 2526.31 | 1668.79 | 3266.37 | 1142.00 |
| ENSG00000184809 | B3GALT5-AS1 | 16.91 | 9.20 | 7.36 | 3.03 |
| ENSG00000203930 | LINC00632 | 0.86 | 0.05 | 4.09 | 1.56 |
| ENSG00000231584 | FAHD2CP | 10.87 | 3.96 | 11.42 | 4.19 |
| ENSG00000182774 | RPS17 | 128.53 | 87.74 | 370.48 | 134.87 |
| ENSG00000176978 | DPP7 | 24.70 | 5.87 | 71.05 | 25.75 |
| ENSG00000161243 | FBXO27 | 3.44 | 1.24 | 4.28 | 1.58 |
| ENSG00000114200 | BCHE | 4.87 | 0.21 | 24.72 | 11.50 |
| ENSG00000059378 | PARP12 | 1.84 | 0.48 | 2.16 | 0.82 |
| ENSG00000008394 | MGST1 | 4.33 | 0.10 | 8.12 | 3.03 |
| ENSG00000167658 | EEF2 | 915.99 | 678.44 | 2364.93 | 869.83 |
| ENSG00000101224 | CDC25B | 37.60 | 18.53 | 40.39 | 16.96 |
| ENSG00000111728 | ST8SIA1 | 1.82 | 0.38 | 1.85 | 0.72 |
| ENSG00000072210 | ALDH3A2 | 69.92 | 35.81 | 39.45 | 15.46 |
| ENSG00000240342 | AC026366.1 | 192.26 | 146.66 | 487.17 | 180.52 |
| ENSG00000137818 | RPLP1 | 314.53 | 255.94 | 901.01 | 333.31 |
| ENSG00000225630 | MTND2P28 | 41.41 | 30.44 | 57.77 | 20.71 |
| ENSG00000089157 | RPLP0 | 455.38 | 318.78 | 1324.58 | 504.04 |
| ENSG00000143947 | RPS27A | 274.23 | 214.44 | 833.98 | 319.14 |
| ENSG00000177706 | FAM20C | 67.06 | 27.93 | 71.85 | 27.71 |
| ENSG00000145675 | PIK3R1 | 11.38 | 7.34 | 21.18 | 7.66 |
| ENSG00000135144 | DTX1 | 4.08 | 0.31 | 1.78 | 0.68 |
| ENSG00000175175 | PPM1E | 2.58 | 1.77 | 4.95 | 1.87 |
| ENSG00000164929 | BAALC | 14.69 | 3.59 | 9.49 | 4.67 |
| ENSG00000185201 | IFITM2 | 48.85 | 17.34 | 148.45 | 56.05 |
| ENSG00000174748 | RPL15 | 272.84 | 207.90 | 608.73 | 238.93 |
| ENSG00000228623 | ZNF883 | 3.96 | 2.80 | 1.64 | 0.63 |
| ENSG00000186868 | MAPT | 7.17 | 2.14 | 3.09 | 1.19 |
| ENSG00000175899 | A2M | 538.03 | 79.58 | 476.87 | 193.05 |
| ENSG00000147862 | NFIB | 34.46 | 10.44 | 22.66 | 9.08 |
| ENSG00000107833 | NPM3 | 15.24 | 10.65 | 33.81 | 13.37 |
| ENSG00000196205 | EEF1A1P5 | 22.16 | 14.78 | 42.65 | 16.49 |
| ENSG00000070756 | PABPC1 | 168.76 | 138.72 | 577.45 | 229.30 |
| ENSG00000168913 | ENHO | 41.54 | 28.87 | 13.30 | 5.83 |
| ENSG00000198888 | MT-ND1 | 1530.91 | 1209.24 | 2392.51 | 930.02 |
| ENSG00000168028 | RPSA | 278.63 | 203.61 | 755.71 | 305.40 |
| ENSG00000165474 | GJB2 | 21.89 | 1.06 | 4.80 | 1.90 |
| ENSG00000198763 | MT-ND2 | 1990.86 | 1414.40 | 2546.60 | 979.61 |
| ENSG00000131095 | GFAP | 2767.81 | 251.01 | 934.76 | 379.46 |
| ENSG00000185652 | NTF3 | 2.89 | 0.03 | 5.41 | 2.19 |
| ENSG00000110841 | PPFIBP1 | 10.97 | 7.88 | 28.99 | 11.62 |
| ENSG00000198804 | MT-CO1 | 7729.64 | 5821.07 | 10047.04 | 3861.96 |
| ENSG00000180011 | ZADH2 | 6.40 | 3.91 | 7.61 | 3.18 |
| ENSG00000245060 | LINC00847 | 9.77 | 0.30 | 7.93 | 3.26 |
| ENSG00000155511 | GRIA1 | 139.76 | 80.17 | 92.83 | 37.19 |
| ENSG00000181163 | NPM1 | 193.26 | 154.47 | 498.05 | 204.44 |
| ENSG00000137098 | SPAG8 | 2.37 | 1.47 | 1.94 | 0.84 |
| ENSG00000175497 | DPP10 | 2.30 | 0.00 | 2.47 | 1.05 |
| ENSG00000119139 | TJP2 | 13.70 | 3.41 | 4.64 | 1.88 |
| ENSG00000204264 | PSMB8 | 30.52 | 19.04 | 17.34 | 8.06 |
| ENSG00000143842 | SOX13 | 10.78 | 7.85 | 4.14 | 1.73 |
| ENSG00000115738 | ID2 | 21.87 | 9.85 | 50.91 | 22.48 |
| ENSG00000110881 | ASIC1 | 11.51 | 3.58 | 9.89 | 4.36 |
| ENSG00000136826 | KLF4 | 1.28 | 0.15 | 9.60 | 4.12 |
| ENSG00000272398 | CD24 | 354.30 | 65.83 | 16.50 | 7.17 |
| ENSG00000107829 | FBXW4 | 3.08 | 0.51 | 12.73 | 5.45 |
| ENSG00000135540 | NHSL1 | 14.61 | 3.94 | 5.19 | 2.25 |
| ENSG00000135245 | HILPDA | 7.09 | 4.79 | 14.09 | 6.07 |
| ENSG00000146281 | PM20D2 | 4.21 | 3.16 | 6.21 | 2.89 |
| ENSG00000152518 | ZFP36L2 | 31.53 | 15.14 | 24.87 | 11.75 |
| ENSG00000156508 | EEF1A1 | 2412.20 | 1456.59 | 4416.49 | 1887.13 |
| ENSG00000063046 | EIF4B | 104.23 | 75.93 | 198.76 | 84.32 |
| ENSG00000106624 | AEBP1 | 179.99 | 77.70 | 103.57 | 47.50 |
| ENSG00000184465 | WDR27 | 7.47 | 5.47 | 9.74 | 4.23 |
| ENSG00000278970 | HEIH | 6.90 | 0.23 | 9.60 | 4.26 |
| ENSG00000227063 | RPL41P1 | 126.00 | 80.36 | 281.79 | 125.74 |
| ENSG00000177519 | RPRM | 0.58 | 0.03 | 5.11 | 2.31 |
| ENSG00000102024 | PLS3 | 218.34 | 45.21 | 268.06 | 117.61 |
| ENSG00000143409 | MINDY1 | 15.90 | 10.70 | 12.58 | 5.86 |
| ENSG00000099204 | ABLIM1 | 29.51 | 8.80 | 53.79 | 24.34 |
| ENSG00000130382 | MLLT1 | 48.50 | 28.13 | 49.28 | 22.75 |
| ENSG00000206561 | COLQ | 3.39 | 1.71 | 2.31 | 0.99 |
| ENSG00000129654 | FOXJ1 | 21.81 | 2.77 | 4.45 | 2.73 |
| ENSG00000101236 | RNF24 | 11.22 | 5.48 | 15.45 | 6.93 |
| ENSG00000127989 | MTERF1 | 4.36 | 1.73 | 3.96 | 1.83 |
| ENSG00000042493 | CAPG | 25.54 | 0.63 | 11.52 | 5.43 |
| ENSG00000198727 | MT-CYB | 3038.93 | 2296.48 | 4300.44 | 1889.91 |
| ENSG00000197568 | HHLA3 | 33.61 | 12.10 | 21.46 | 9.98 |
| ENSG00000133943 | C14orf159 | 27.31 | 21.06 | 15.50 | 7.74 |
| ENSG00000078295 | ADCY2 | 11.10 | 6.38 | 7.91 | 3.85 |
| ENSG00000113119 | TMCO6 | 3.38 | 1.92 | 5.57 | 2.64 |
| ENSG00000278845 | MRPL45 | 16.24 | 13.00 | 30.10 | 14.91 |
| ENSG00000113552 | GNPDA1 | 41.10 | 0.14 | 32.91 | 16.46 |
| ENSG00000231806 | PCAT7 | 15.79 | 10.20 | 11.72 | 5.96 |
| ENSG00000183111 | ARHGEF37 | 7.02 | 1.71 | 2.50 | 1.29 |
| ENSG00000026508 | CD44 | 492.74 | 372.91 | 308.52 | 153.77 |
| ENSG00000165416 | SUGT1 | 4.19 | 2.77 | 6.44 | 3.49 |
| ENSG00000142599 | RERE | 21.51 | 15.77 | 25.75 | 13.79 |
| ENSG00000166925 | TSC22D4 | 29.19 | 23.45 | 42.12 | 22.65 |
| ENSG00000106992 | AK1 | 3.82 | 2.00 | 5.35 | 2.97 |
| ENSG00000088280 | ASAP3 | 35.34 | 25.69 | 36.38 | 20.34 |
| ENSG00000115525 | ST3GAL5 | 6.47 | 1.35 | 2.79 | 1.66 |
