# Supplementary material for: Mutant C9orf72 human iPSC‐derived astrocytes cause non‐cell autonomous motor neuron pathophysiology
Source: Glia. 2019 Dec 16;68(5):1046–64. doi: 10.1002/glia.23761 (PMC7078830; doi:10.1002/glia.23761)
Supplement: Supplementary file 9 — Figure S9 List of genes that are significantly downregulated in C9ORF72 mutant astrocytes (FDR 0.1) [file GLIA-68-1046-s009.docx]

**Supplementary Figure 9. List of genes that are significantly downregulated in C9ORF72 mutant astrocytes (FDR 0.1)**

| **Gene** | **Gene Name** | **C9-2** | **C9-Δ2** | **C9-3** | **C9-Δ3** |
| --- | --- | --- | --- | --- | --- |
|  |  |  |  |  |  |
| ENSG00000160293 | VAV2 | 15.01 | 27.99 | 13.54 | 18.12 |
| ENSG00000131378 | RFTN1 | 43.17 | 84.19 | 39.94 | 53.80 |
| ENSG00000088854 | C20orf194 | 8.92 | 13.58 | 5.09 | 6.87 |
| ENSG00000133216 | EPHB2 | 7.19 | 20.13 | 6.49 | 8.75 |
| ENSG00000145555 | MYO10 | 7.35 | 11.98 | 6.70 | 9.23 |
| ENSG00000136274 | NACAD | 6.62 | 11.25 | 5.39 | 7.53 |
| ENSG00000103005 | USB1 | 15.39 | 22.31 | 12.51 | 17.60 |
| ENSG00000198585 | NUDT16 | 10.07 | 15.72 | 6.51 | 9.11 |
| ENSG00000092421 | SEMA6A | 24.08 | 44.02 | 14.96 | 21.05 |
| ENSG00000167657 | DAPK3 | 21.80 | 30.90 | 26.61 | 37.31 |
| ENSG00000179588 | ZFPM1 | 0.96 | 1.71 | 0.88 | 1.26 |
| ENSG00000183688 | RFLNB | 1.62 | 4.10 | 5.97 | 8.72 |
| ENSG00000033170 | FUT8 | 13.08 | 23.71 | 8.15 | 11.88 |
| ENSG00000185361 | TNFAIP8L1 | 1.77 | 3.47 | 1.01 | 1.61 |
| ENSG00000197381 | ADARB1 | 3.63 | 6.75 | 3.87 | 5.75 |
| ENSG00000100504 | PYGL | 18.11 | 32.12 | 16.24 | 24.75 |
| ENSG00000163412 | EIF4E3 | 1.62 | 2.62 | 1.24 | 1.80 |
| ENSG00000075624 | ACTB | 1386.96 | 1994.40 | 1587.04 | 2327.59 |
| ENSG00000130158 | DOCK6 | 5.80 | 9.71 | 5.91 | 8.77 |
| ENSG00000109046 | WSB1 | 75.74 | 124.35 | 65.06 | 95.35 |
| ENSG00000164620 | RELL2 | 2.45 | 4.03 | 1.14 | 1.81 |
| ENSG00000153046 | CDYL | 8.90 | 12.74 | 6.13 | 9.23 |
| ENSG00000237765 | FAM200B | 12.30 | 24.94 | 6.29 | 9.74 |
| ENSG00000166086 | JAM3 | 51.41 | 87.90 | 32.69 | 50.74 |
| ENSG00000090097 | PCBP4 | 39.78 | 72.02 | 36.10 | 55.63 |
| ENSG00000099139 | PCSK5 | 11.52 | 18.32 | 2.46 | 3.89 |
| ENSG00000096968 | JAK2 | 2.85 | 4.48 | 1.84 | 2.84 |
| ENSG00000100842 | EFS | 7.80 | 23.05 | 11.29 | 18.92 |
| ENSG00000088899 | LZTS3 | 4.04 | 8.35 | 2.87 | 4.51 |
| ENSG00000099625 | CBARP | 3.19 | 7.13 | 2.72 | 4.39 |
| ENSG00000129244 | ATP1B2 | 104.27 | 207.49 | 28.55 | 46.47 |
| ENSG00000001461 | NIPAL3 | 17.19 | 38.21 | 9.28 | 14.31 |
| ENSG00000094804 | CDC6 | 0.45 | 1.32 | 1.70 | 2.72 |
| ENSG00000141429 | GALNT1 | 63.86 | 109.80 | 44.51 | 68.82 |
| ENSG00000048740 | CELF2 | 16.05 | 26.51 | 7.04 | 11.42 |
| ENSG00000224081 | SLC44A3-AS1 | 1.76 | 2.88 | 0.36 | 0.60 |
| ENSG00000205413 | SAMD9 | 2.35 | 3.75 | 0.73 | 1.25 |
| ENSG00000177606 | JUN | 29.35 | 56.11 | 25.65 | 41.61 |
| ENSG00000056972 | TRAF3IP2 | 2.90 | 7.99 | 1.90 | 3.16 |
| ENSG00000164877 | MICALL2 | 7.08 | 13.98 | 5.45 | 8.85 |
| ENSG00000157600 | TMEM164 | 13.57 | 24.76 | 5.51 | 9.15 |
| ENSG00000171067 | C11orf24 | 16.84 | 27.87 | 22.83 | 38.36 |
| ENSG00000118503 | TNFAIP3 | 0.95 | 1.51 | 0.82 | 1.41 |
| ENSG00000137166 | FOXP4 | 5.57 | 13.14 | 5.21 | 8.60 |
| ENSG00000198795 | ZNF521 | 10.92 | 25.45 | 8.97 | 16.51 |
| ENSG00000108018 | SORCS1 | 2.29 | 41.00 | 1.81 | 2.97 |
| ENSG00000182957 | SPATA13 | 4.24 | 6.91 | 2.12 | 3.66 |
| ENSG00000075945 | KIFAP3 | 25.88 | 37.65 | 12.71 | 21.56 |
| ENSG00000020577 | SAMD4A | 7.19 | 15.05 | 6.97 | 11.76 |
| ENSG00000148429 | USP6NL | 3.31 | 5.98 | 2.32 | 4.01 |
| ENSG00000175567 | UCP2 | 7.88 | 12.87 | 6.18 | 10.41 |
| ENSG00000159840 | ZYX | 47.12 | 70.57 | 114.08 | 198.62 |
| ENSG00000130294 | KIF1A | 24.23 | 67.86 | 7.57 | 14.21 |
| ENSG00000164970 | FAM219A | 18.06 | 29.33 | 8.10 | 14.20 |
| ENSG00000177409 | SAMD9L | 6.20 | 12.24 | 2.74 | 4.84 |
| ENSG00000105329 | TGFB1 | 28.09 | 54.04 | 21.97 | 39.19 |
| ENSG00000279821 | AC145098.2 | 0.72 | 1.44 | 1.73 | 2.89 |
| ENSG00000138193 | PLCE1 | 3.70 | 5.70 | 1.77 | 3.19 |
| ENSG00000168394 | TAP1 | 26.74 | 40.23 | 10.07 | 18.17 |
| ENSG00000188158 | NHS | 5.42 | 10.08 | 1.51 | 2.63 |
| ENSG00000175662 | TOM1L2 | 36.78 | 62.41 | 26.16 | 46.52 |
| ENSG00000119403 | PHF19 | 3.60 | 7.71 | 2.67 | 5.09 |
| ENSG00000185386 | MAPK11 | 13.28 | 22.27 | 10.30 | 18.13 |
| ENSG00000105255 | FSD1 | 6.46 | 12.37 | 3.20 | 5.99 |
| ENSG00000198517 | MAFK | 3.37 | 6.34 | 5.90 | 10.57 |
| ENSG00000171314 | PGAM1 | 51.69 | 73.85 | 46.15 | 83.02 |
| ENSG00000142235 | LMTK3 | 2.88 | 5.13 | 0.96 | 1.81 |
| ENSG00000174672 | BRSK2 | 0.87 | 2.15 | 1.03 | 2.49 |
| ENSG00000111666 | CHPT1 | 13.75 | 27.20 | 8.86 | 16.51 |
| ENSG00000137996 | RTCA | 12.40 | 17.64 | 9.40 | 16.83 |
| ENSG00000272341 | AL137003.2 | 1.90 | 6.31 | 1.44 | 2.71 |
| ENSG00000205542 | TMSB4X | 837.10 | 1376.51 | 298.51 | 533.07 |
| ENSG00000107438 | PDLIM1 | 34.78 | 66.96 | 37.48 | 65.68 |
| ENSG00000182749 | PAQR7 | 6.20 | 11.29 | 4.49 | 8.06 |
| ENSG00000162976 | PQLC3 | 8.69 | 14.19 | 6.54 | 12.01 |
| ENSG00000092871 | RFFL | 3.18 | 5.14 | 1.59 | 2.92 |
| ENSG00000126878 | AIF1L | 8.47 | 22.51 | 6.25 | 14.24 |
| ENSG00000115457 | IGFBP2 | 57.67 | 105.93 | 92.19 | 171.37 |
| ENSG00000099992 | TBC1D10A | 1.47 | 2.95 | 1.90 | 3.58 |
| ENSG00000198961 | PJA2 | 74.02 | 111.32 | 34.56 | 63.28 |
| ENSG00000105928 | DFNA5 | 9.32 | 13.52 | 5.30 | 9.90 |
| ENSG00000114268 | PFKFB4 | 5.58 | 9.07 | 2.44 | 4.64 |
| ENSG00000076513 | ANKRD13A | 18.36 | 34.51 | 12.40 | 23.60 |
| ENSG00000036530 | CYP46A1 | 2.73 | 4.67 | 0.60 | 1.19 |
| ENSG00000157110 | RBPMS | 8.22 | 21.02 | 8.77 | 16.36 |
| ENSG00000145386 | CCNA2 | 1.30 | 3.18 | 2.90 | 6.32 |
| ENSG00000186352 | ANKRD37 | 0.99 | 2.05 | 1.89 | 3.77 |
| ENSG00000060656 | PTPRU | 1.33 | 6.07 | 5.39 | 10.78 |
| ENSG00000069974 | RAB27A | 3.15 | 7.87 | 1.81 | 3.40 |
| ENSG00000007866 | TEAD3 | 8.84 | 13.91 | 11.05 | 20.52 |
| ENSG00000114861 | FOXP1 | 0.78 | 1.86 | 6.09 | 11.39 |
| ENSG00000163251 | FZD5 | 3.28 | 5.98 | 3.39 | 6.70 |
| ENSG00000236824 | BCYRN1 | 48.30 | 93.35 | 42.63 | 81.55 |
| ENSG00000166173 | LARP6 | 4.13 | 6.04 | 5.16 | 10.17 |
| ENSG00000163873 | GRIK3 | 6.48 | 23.37 | 5.19 | 9.88 |
| ENSG00000087053 | MTMR2 | 10.83 | 17.40 | 10.39 | 20.52 |
| ENSG00000178445 | GLDC | 33.05 | 59.43 | 7.75 | 15.91 |
| ENSG00000196923 | PDLIM7 | 64.02 | 130.53 | 127.90 | 244.33 |
| ENSG00000006327 | TNFRSF12A | 33.42 | 65.58 | 56.66 | 109.18 |
| ENSG00000146112 | PPP1R18 | 17.42 | 25.58 | 15.14 | 29.47 |
| ENSG00000139641 | ESYT1 | 19.66 | 29.92 | 20.16 | 40.25 |
| ENSG00000213626 | LBH | 12.46 | 26.57 | 22.77 | 51.13 |
| ENSG00000107796 | ACTA2 | 222.42 | 401.22 | 1000.96 | 2021.51 |
| ENSG00000167703 | SLC43A2 | 22.22 | 32.92 | 13.10 | 26.22 |
| ENSG00000213390 | ARHGAP19 | 3.92 | 6.28 | 1.71 | 3.92 |
| ENSG00000160326 | SLC2A6 | 3.83 | 8.16 | 3.80 | 7.78 |
| ENSG00000185022 | MAFF | 0.55 | 1.29 | 1.60 | 3.18 |
| ENSG00000104368 | PLAT | 20.73 | 47.59 | 6.47 | 12.95 |
| ENSG00000167100 | SAMD14 | 3.08 | 9.70 | 2.85 | 5.30 |
| ENSG00000012660 | ELOVL5 | 39.54 | 89.53 | 28.63 | 59.18 |
| ENSG00000161513 | FDXR | 19.25 | 28.53 | 6.71 | 13.62 |
| ENSG00000105825 | TFPI2 | 0.39 | 3.38 | 10.65 | 22.09 |
| ENSG00000112852 | PCDHB2 | 16.02 | 30.54 | 10.06 | 21.38 |
| ENSG00000140839 | CLEC18B | 28.66 | 64.49 | 12.81 | 26.86 |
| ENSG00000269834 | ZNF528-AS1 | 4.76 | 7.97 | 3.28 | 7.80 |
| ENSG00000091129 | NRCAM | 45.61 | 87.24 | 7.35 | 14.81 |
| ENSG00000118263 | KLF7 | 3.18 | 7.63 | 3.90 | 8.14 |
| ENSG00000143367 | TUFT1 | 6.98 | 12.45 | 12.85 | 27.76 |
| ENSG00000067057 | PFKP | 19.81 | 54.97 | 19.16 | 42.09 |
| ENSG00000052802 | MSMO1 | 25.08 | 43.69 | 22.93 | 47.34 |
| ENSG00000107518 | ATRNL1 | 0.85 | 4.89 | 1.52 | 3.25 |
| ENSG00000167614 | TTYH1 | 74.74 | 162.68 | 17.15 | 50.54 |
| ENSG00000187608 | ISG15 | 16.91 | 51.07 | 8.28 | 17.90 |
| ENSG00000109089 | CDR2L | 5.28 | 9.85 | 6.31 | 13.82 |
| ENSG00000277531 | PNMA8C | 1.79 | 3.24 | 0.70 | 1.74 |
| ENSG00000115556 | PLCD4 | 2.37 | 6.28 | 1.78 | 3.78 |
| ENSG00000187164 | SHTN1 | 14.62 | 27.14 | 4.02 | 10.05 |
| ENSG00000005469 | CROT | 18.62 | 32.30 | 4.21 | 9.71 |
| ENSG00000131979 | GCH1 | 1.36 | 4.14 | 0.64 | 1.56 |
| ENSG00000261786 | AC006058.1 | 8.55 | 26.49 | 1.62 | 3.96 |
| ENSG00000175352 | NRIP3 | 4.53 | 25.08 | 0.47 | 1.06 |
| ENSG00000157613 | CREB3L1 | 4.69 | 9.22 | 19.40 | 44.73 |
| ENSG00000239268 | AC092691.1 | 1.87 | 5.21 | 0.89 | 2.02 |
| ENSG00000146592 | CREB5 | 2.17 | 3.73 | 1.49 | 3.40 |
| ENSG00000150672 | DLG2 | 1.33 | 2.76 | 1.29 | 3.31 |
| ENSG00000137460 | FHDC1 | 2.20 | 3.94 | 1.33 | 2.98 |
| ENSG00000125430 | HS3ST3B1 | 5.91 | 17.27 | 0.97 | 2.13 |
| ENSG00000179820 | MYADM | 21.58 | 36.37 | 33.59 | 77.42 |
| ENSG00000147852 | VLDLR | 24.14 | 33.42 | 11.87 | 26.81 |
| ENSG00000244405 | ETV5 | 6.71 | 17.92 | 8.96 | 19.93 |
| ENSG00000183098 | GPC6 | 16.99 | 32.79 | 11.22 | 26.14 |
| ENSG00000139910 | NOVA1 | 5.03 | 9.03 | 1.82 | 5.12 |
| ENSG00000255737 | AGAP2-AS1 | 1.77 | 7.54 | 0.30 | 0.70 |
| ENSG00000274964 | AC026356.1 | 1.44 | 2.62 | 0.98 | 2.34 |
| ENSG00000124225 | PMEPA1 | 30.33 | 53.05 | 24.59 | 61.15 |
| ENSG00000196358 | NTNG2 | 1.75 | 11.02 | 0.60 | 1.42 |
| ENSG00000111252 | SH2B3 | 5.52 | 9.91 | 3.15 | 7.41 |
| ENSG00000072682 | P4HA2 | 13.14 | 31.95 | 40.61 | 104.26 |
| ENSG00000144366 | GULP1 | 0.42 | 1.08 | 0.87 | 2.10 |
| ENSG00000119729 | RHOQ | 26.50 | 45.06 | 18.61 | 45.25 |
| ENSG00000057019 | DCBLD2 | 54.13 | 98.06 | 17.25 | 41.79 |
| ENSG00000203867 | RBM20 | 2.94 | 5.07 | 0.88 | 2.16 |
| ENSG00000163888 | CAMK2N2 | 1.03 | 4.33 | 1.08 | 2.90 |
| ENSG00000197093 | GAL3ST4 | 11.51 | 21.11 | 3.96 | 9.78 |
| ENSG00000054967 | RELT | 2.61 | 4.69 | 1.83 | 4.83 |
| ENSG00000109189 | USP46 | 7.09 | 10.96 | 3.53 | 8.74 |
| ENSG00000250091 | DNAH10OS | 1.62 | 3.22 | 0.84 | 2.23 |
| ENSG00000144810 | COL8A1 | 9.37 | 14.84 | 65.60 | 164.08 |
| ENSG00000198910 | L1CAM | 4.61 | 58.35 | 2.44 | 10.20 |
| ENSG00000151746 | BICD1 | 7.45 | 16.87 | 3.19 | 8.03 |
| ENSG00000162188 | GNG3 | 5.87 | 20.00 | 1.38 | 3.19 |
| ENSG00000108691 | CCL2 | 34.36 | 72.00 | 82.30 | 198.27 |
| ENSG00000139324 | TMTC3 | 7.29 | 12.14 | 2.60 | 6.47 |
| ENSG00000011638 | TMEM159 | 7.57 | 14.94 | 5.71 | 14.37 |
| ENSG00000005238 | FAM214B | 11.43 | 20.10 | 7.02 | 18.14 |
| ENSG00000173846 | PLK3 | 5.98 | 8.73 | 5.11 | 13.20 |
| ENSG00000180611 | MB21D2 | 1.78 | 3.14 | 1.59 | 4.22 |
| ENSG00000164574 | GALNT10 | 75.51 | 164.87 | 39.42 | 103.52 |
| ENSG00000106688 | SLC1A1 | 0.57 | 3.79 | 1.07 | 2.87 |
| ENSG00000131409 | LRRC4B | 19.30 | 36.64 | 8.53 | 24.18 |
| ENSG00000177707 | NECTIN3 | 12.53 | 22.08 | 5.18 | 12.89 |
| ENSG00000143013 | LMO4 | 18.75 | 33.02 | 9.92 | 27.04 |
| ENSG00000126458 | RRAS | 10.81 | 25.79 | 17.55 | 48.08 |
| ENSG00000279041 | AC102945.2 | 0.81 | 2.26 | 0.50 | 1.30 |
| ENSG00000127252 | HRASLS | 2.59 | 5.79 | 0.62 | 1.91 |
| ENSG00000076706 | MCAM | 19.76 | 33.69 | 19.22 | 50.77 |
| ENSG00000167178 | ISLR2 | 0.85 | 2.48 | 1.39 | 3.60 |
| ENSG00000110876 | SELPLG | 0.41 | 1.19 | 1.18 | 3.19 |
| ENSG00000073712 | FERMT2 | 23.58 | 36.20 | 18.72 | 48.88 |
| ENSG00000127124 | HIVEP3 | 1.66 | 2.99 | 0.57 | 1.53 |
| ENSG00000172020 | GAP43 | 64.58 | 141.57 | 11.11 | 29.61 |
| ENSG00000105290 | APLP1 | 79.02 | 157.54 | 25.49 | 68.80 |
| ENSG00000197959 | DNM3 | 1.44 | 3.21 | 0.72 | 1.99 |
| ENSG00000116717 | GADD45A | 23.98 | 51.26 | 17.69 | 47.55 |
| ENSG00000240764 | PCDHGC5 | 3.14 | 6.57 | 1.89 | 4.77 |
| ENSG00000250320 | AC113383.1 | 0.03 | 0.69 | 1.19 | 3.09 |
| ENSG00000185070 | FLRT2 | 0.12 | 0.42 | 1.68 | 5.27 |
| ENSG00000136378 | ADAMTS7 | 3.12 | 7.69 | 3.47 | 9.22 |
| ENSG00000182287 | AP1S2 | 161.46 | 241.32 | 17.76 | 53.19 |
| ENSG00000159674 | SPON2 | 1.94 | 13.21 | 4.02 | 11.45 |
| ENSG00000117228 | GBP1 | 8.14 | 16.38 | 3.83 | 11.02 |
| ENSG00000164308 | ERAP2 | 12.21 | 18.07 | 1.51 | 4.41 |
| ENSG00000158186 | MRAS | 10.55 | 16.30 | 9.92 | 27.63 |
| ENSG00000113319 | RASGRF2 | 5.08 | 11.83 | 2.84 | 8.13 |
| ENSG00000162458 | FBLIM1 | 8.41 | 18.68 | 9.17 | 26.13 |
| ENSG00000144136 | SLC20A1 | 15.57 | 27.53 | 10.68 | 30.55 |
| ENSG00000187678 | SPRY4 | 0.91 | 1.78 | 2.04 | 6.00 |
| ENSG00000182667 | NTM | 8.91 | 16.45 | 10.25 | 31.82 |
| ENSG00000153404 | PLEKHG4B | 7.14 | 13.84 | 0.53 | 1.52 |
| ENSG00000130635 | COL5A1 | 18.43 | 43.23 | 140.54 | 470.16 |
| ENSG00000075711 | DLG1 | 18.41 | 45.69 | 10.86 | 32.91 |
| ENSG00000157168 | NRG1 | 1.43 | 8.90 | 5.58 | 17.79 |
| ENSG00000101198 | NKAIN4 | 7.90 | 34.87 | 3.42 | 11.51 |
| ENSG00000181649 | PHLDA2 | 1.30 | 4.15 | 3.50 | 10.96 |
| ENSG00000247095 | MIR210HG | 0.97 | 2.63 | 2.73 | 8.84 |
| ENSG00000122863 | CHST3 | 47.42 | 98.10 | 17.25 | 54.33 |
| ENSG00000214517 | PPME1 | 20.19 | 33.86 | 27.23 | 88.00 |
| ENSG00000175592 | FOSL1 | 2.53 | 6.10 | 1.21 | 3.80 |
| ENSG00000168528 | SERINC2 | 9.79 | 26.92 | 3.99 | 12.64 |
| ENSG00000176463 | SLCO3A1 | 2.51 | 5.25 | 0.86 | 2.63 |
| ENSG00000100505 | TRIM9 | 17.17 | 40.92 | 4.94 | 19.76 |
| ENSG00000272841 | AL139393.2 | 14.17 | 56.15 | 10.26 | 36.86 |
| ENSG00000109472 | CPE | 30.35 | 100.84 | 34.31 | 106.58 |
| ENSG00000188766 | SPRED3 | 1.35 | 3.24 | 1.53 | 4.83 |
| ENSG00000138821 | SLC39A8 | 0.67 | 1.90 | 0.75 | 2.52 |
| ENSG00000125355 | TMEM255A | 16.25 | 30.20 | 1.90 | 6.98 |
| ENSG00000144802 | NFKBIZ | 1.59 | 4.51 | 3.64 | 12.41 |
| ENSG00000148604 | RGR | 2.02 | 4.74 | 0.15 | 0.57 |
| ENSG00000088836 | SLC4A11 | 1.84 | 4.30 | 0.26 | 0.87 |
| ENSG00000105251 | SHD | 0.50 | 1.58 | 0.48 | 3.09 |
| ENSG00000128606 | LRRC17 | 15.76 | 38.15 | 12.37 | 45.61 |
| ENSG00000222041 | CYTOR | 12.22 | 18.23 | 12.60 | 41.33 |
| ENSG00000146197 | SCUBE3 | 0.53 | 3.81 | 1.77 | 5.81 |
| ENSG00000251450 | RASGRF2-AS1 | 0.79 | 2.75 | 0.59 | 2.20 |
| ENSG00000071575 | TRIB2 | 12.94 | 22.75 | 11.03 | 35.84 |
| ENSG00000072163 | LIMS2 | 1.14 | 3.68 | 3.89 | 13.16 |
| ENSG00000165868 | HSPA12A | 12.82 | 71.77 | 3.81 | 12.49 |
| ENSG00000138311 | ZNF365 | 2.59 | 5.73 | 0.89 | 3.49 |
| ENSG00000117069 | ST6GALNAC5 | 11.00 | 34.75 | 5.06 | 19.12 |
| ENSG00000177283 | FZD8 | 2.60 | 9.53 | 4.63 | 16.53 |
| ENSG00000117226 | GBP3 | 10.17 | 21.35 | 2.19 | 7.24 |
| ENSG00000123560 | PLP1 | 121.35 | 199.31 | 12.23 | 48.86 |
| ENSG00000243232 | PCDHAC2 | 2.33 | 4.11 | 1.13 | 3.89 |
| ENSG00000204262 | COL5A2 | 27.17 | 103.38 | 78.13 | 284.07 |
| ENSG00000174721 | FGFBP3 | 6.70 | 18.07 | 3.32 | 13.68 |
| ENSG00000104267 | CA2 | 9.54 | 136.15 | 2.79 | 11.00 |
| ENSG00000115641 | FHL2 | 6.46 | 10.08 | 6.82 | 24.90 |
| ENSG00000261115 | TMEM178B | 7.03 | 12.67 | 1.06 | 3.92 |
| ENSG00000029153 | ARNTL2 | 2.65 | 4.18 | 0.93 | 3.33 |
| ENSG00000132718 | SYT11 | 155.98 | 238.69 | 24.76 | 88.46 |
| ENSG00000166831 | RBPMS2 | 0.81 | 2.28 | 0.85 | 2.98 |
| ENSG00000146216 | TTBK1 | 2.95 | 6.63 | 0.63 | 2.42 |
| ENSG00000003249 | DBNDD1 | 6.75 | 49.33 | 4.95 | 18.27 |
| ENSG00000183023 | SLC8A1 | 1.31 | 2.31 | 0.71 | 2.47 |
| ENSG00000140511 | HAPLN3 | 0.99 | 3.61 | 4.87 | 18.62 |
| ENSG00000105974 | CAV1 | 15.38 | 28.66 | 10.26 | 36.47 |
| ENSG00000126785 | RHOJ | 14.80 | 24.91 | 5.60 | 20.51 |
| ENSG00000120318 | ARAP3 | 1.20 | 4.59 | 0.48 | 1.75 |
| ENSG00000087085 | ACHE | 3.31 | 9.98 | 0.67 | 2.57 |
| ENSG00000235162 | C12orf75 | 5.70 | 11.86 | 5.02 | 19.27 |
| ENSG00000121900 | TMEM54 | 9.35 | 22.34 | 3.31 | 11.82 |
| ENSG00000140416 | TPM1 | 109.46 | 176.08 | 158.84 | 599.44 |
| ENSG00000042062 | RIPOR3 | 1.19 | 5.33 | 0.06 | 0.43 |
| ENSG00000188290 | HES4 | 7.36 | 17.48 | 4.24 | 16.53 |
| ENSG00000070404 | FSTL3 | 8.53 | 22.73 | 13.82 | 55.69 |
| ENSG00000137331 | IER3 | 22.72 | 41.86 | 37.51 | 145.86 |
| ENSG00000253910 | PCDHGB2 | 0.71 | 2.60 | 0.54 | 2.06 |
| ENSG00000166863 | TAC3 | 1.29 | 5.93 | 1.39 | 5.87 |
| ENSG00000115884 | SDC1 | 6.37 | 23.12 | 5.13 | 17.90 |
| ENSG00000131037 | EPS8L1 | 0.46 | 2.20 | 0.31 | 1.40 |
| ENSG00000113448 | PDE4D | 0.78 | 3.60 | 1.67 | 6.61 |
| ENSG00000112183 | RBM24 | 2.33 | 4.33 | 0.74 | 3.11 |
| ENSG00000095587 | TLL2 | 1.69 | 3.32 | 0.46 | 1.91 |
| ENSG00000196376 | SLC35F1 | 28.66 | 48.71 | 8.70 | 37.29 |
| ENSG00000184524 | CEND1 | 8.34 | 18.98 | 2.23 | 9.32 |
| ENSG00000127863 | TNFRSF19 | 22.67 | 88.76 | 12.17 | 52.65 |
| ENSG00000106976 | DNM1 | 10.34 | 20.74 | 3.82 | 16.00 |
| ENSG00000182010 | RTKN2 | 0.61 | 1.83 | 0.43 | 2.09 |
| ENSG00000168280 | KIF5C | 29.34 | 54.08 | 5.34 | 24.38 |
| ENSG00000139926 | FRMD6 | 3.85 | 10.57 | 6.07 | 29.05 |
| ENSG00000189060 | H1F0 | 3.27 | 12.78 | 3.92 | 17.13 |
| ENSG00000110446 | SLC15A3 | 1.01 | 2.44 | 0.20 | 0.90 |
| ENSG00000171819 | ANGPTL7 | 0.58 | 2.46 | 0.60 | 4.23 |
| ENSG00000079156 | OSBPL6 | 3.87 | 6.36 | 0.65 | 2.77 |
| ENSG00000174804 | FZD4 | 2.87 | 5.92 | 1.44 | 6.60 |
| ENSG00000117394 | SLC2A1 | 45.27 | 123.15 | 13.77 | 61.24 |
| ENSG00000139970 | RTN1 | 11.92 | 30.37 | 6.36 | 27.81 |
| ENSG00000135454 | B4GALNT1 | 6.96 | 15.30 | 1.00 | 5.06 |
| ENSG00000140545 | MFGE8 | 66.11 | 128.41 | 66.68 | 309.56 |
| ENSG00000184838 | PRR16 | 0.48 | 2.62 | 1.66 | 7.56 |
| ENSG00000186340 | THBS2 | 1.96 | 10.94 | 10.31 | 99.67 |
| ENSG00000106278 | PTPRZ1 | 57.39 | 86.67 | 9.39 | 46.32 |
| ENSG00000120875 | DUSP4 | 11.50 | 18.55 | 3.48 | 17.35 |
| ENSG00000153976 | HS3ST3A1 | 3.92 | 13.93 | 0.78 | 3.70 |
| ENSG00000173482 | PTPRM | 9.46 | 17.64 | 4.89 | 24.25 |
| ENSG00000133135 | RNF128 | 4.10 | 11.27 | 0.10 | 0.53 |
| ENSG00000113248 | PCDHB15 | 3.44 | 21.97 | 0.67 | 3.73 |
| ENSG00000110436 | SLC1A2 | 2.49 | 5.36 | 0.48 | 2.30 |
| ENSG00000183671 | GPR1 | 1.03 | 3.78 | 1.31 | 7.45 |
| ENSG00000147223 | RIPPLY1 | 1.01 | 4.37 | 0.06 | 0.45 |
| ENSG00000122641 | INHBA | 0.05 | 0.32 | 2.55 | 37.98 |
| ENSG00000165891 | E2F7 | 1.26 | 2.08 | 0.56 | 2.79 |
| ENSG00000164197 | RNF180 | 6.30 | 11.87 | 0.32 | 2.00 |
| ENSG00000047662 | FAM184B | 0.77 | 3.44 | 0.05 | 0.29 |
| ENSG00000280143 | AP000892.3 | 1.47 | 3.81 | 4.94 | 29.59 |
| ENSG00000144476 | ACKR3 | 8.99 | 35.99 | 2.77 | 14.73 |
| ENSG00000148704 | VAX1 | 2.73 | 11.43 | 0.58 | 4.30 |
| ENSG00000152954 | NRSN1 | 0.67 | 4.41 | 0.31 | 1.99 |
| ENSG00000283154 | IQCJ-SCHIP1 | 0.58 | 1.61 | 0.67 | 3.79 |
| ENSG00000173406 | DAB1 | 8.90 | 16.82 | 0.60 | 3.57 |
| ENSG00000122877 | EGR2 | 0.84 | 2.06 | 2.06 | 10.68 |
| ENSG00000151572 | ANO4 | 0.35 | 2.25 | 2.40 | 12.93 |
| ENSG00000248383 | PCDHAC1 | 1.25 | 2.79 | 0.32 | 1.93 |
| ENSG00000179546 | HTR1D | 0.61 | 5.63 | 0.38 | 2.47 |
| ENSG00000172738 | TMEM217 | 1.21 | 2.58 | 0.66 | 4.27 |
| ENSG00000128342 | LIF | 12.53 | 63.22 | 8.95 | 58.28 |
| ENSG00000107551 | RASSF4 | 48.32 | 100.68 | 4.26 | 34.11 |
| ENSG00000280744 | LINC01173 | 7.51 | 34.08 | 0.14 | 1.28 |
| ENSG00000141526 | SLC16A3 | 1.17 | 4.87 | 1.29 | 8.01 |
| ENSG00000253549 | CA3-AS1 | 1.25 | 4.55 | 0.13 | 1.05 |
| ENSG00000159784 | FAM131B | 2.79 | 8.53 | 0.93 | 5.57 |
| ENSG00000237552 | AC099060.1 | 0.34 | 3.81 | 0.12 | 1.38 |
| ENSG00000150687 | PRSS23 | 85.16 | 166.66 | 37.33 | 244.99 |
| ENSG00000113721 | PDGFRB | 3.89 | 8.47 | 7.97 | 53.00 |
| ENSG00000069869 | NEDD4 | 5.69 | 16.28 | 1.78 | 11.14 |
| ENSG00000008517 | IL32 | 1.36 | 6.71 | 0.57 | 4.28 |
| ENSG00000113070 | HBEGF | 3.94 | 8.97 | 8.48 | 59.37 |
| ENSG00000154127 | UBASH3B | 3.66 | 10.79 | 0.57 | 3.70 |
| ENSG00000213949 | ITGA1 | 0.03 | 0.32 | 0.93 | 12.57 |
| ENSG00000230316 | FEZF1-AS1 | 1.50 | 5.40 | 0.83 | 5.76 |
| ENSG00000139278 | GLIPR1 | 11.03 | 31.76 | 3.56 | 24.65 |
| ENSG00000162630 | B3GALT2 | 0.74 | 3.52 | 1.04 | 6.63 |
| ENSG00000266928 | AC020905.1 | 1.81 | 4.25 | 0.18 | 1.85 |
| ENSG00000152952 | PLOD2 | 36.04 | 71.45 | 21.93 | 150.96 |
| ENSG00000254416 | AP000924.1 | 1.71 | 7.30 | 0.14 | 1.45 |
| ENSG00000124762 | CDKN1A | 375.62 | 571.58 | 54.33 | 402.72 |
| ENSG00000178776 | C5orf46 | 7.80 | 13.27 | 6.51 | 51.09 |
| ENSG00000148848 | ADAM12 | 37.22 | 71.38 | 13.42 | 97.19 |
| ENSG00000135842 | FAM129A | 9.27 | 61.07 | 2.50 | 18.72 |
| ENSG00000189058 | APOD | 0.08 | 1.10 | 0.37 | 2.87 |
| ENSG00000101384 | JAG1 | 25.43 | 67.49 | 8.14 | 57.15 |
| ENSG00000117152 | RGS4 | 0.44 | 2.20 | 0.86 | 6.58 |
| ENSG00000074527 | NTN4 | 0.66 | 1.50 | 1.98 | 22.08 |
| ENSG00000105971 | CAV2 | 9.51 | 25.28 | 2.01 | 15.62 |
| ENSG00000156453 | PCDH1 | 4.68 | 10.56 | 2.22 | 17.91 |
| ENSG00000172575 | RASGRP1 | 2.71 | 6.93 | 0.23 | 1.80 |
| ENSG00000086991 | NOX4 | 0.62 | 1.28 | 1.10 | 8.87 |
| ENSG00000196083 | IL1RAP | 6.06 | 13.40 | 6.20 | 48.48 |
| ENSG00000133816 | MICAL2 | 4.70 | 11.11 | 3.88 | 32.81 |
| ENSG00000177875 | CCDC184 | 1.43 | 2.59 | 0.58 | 5.68 |
| ENSG00000135074 | ADAM19 | 7.86 | 15.88 | 5.08 | 46.30 |
| ENSG00000223756 | TSSC2 | 0.09 | 4.37 | 0.12 | 1.09 |
| ENSG00000172403 | SYNPO2 | 0.09 | 0.55 | 0.40 | 13.73 |
| ENSG00000105605 | CACNG7 | 20.51 | 33.11 | 2.34 | 25.49 |
| ENSG00000132205 | EMILIN2 | 0.41 | 2.23 | 0.19 | 2.15 |
| ENSG00000139155 | SLCO1C1 | 1.88 | 3.97 | 0.24 | 3.70 |
| ENSG00000105246 | EBI3 | 0.34 | 3.73 | 0.08 | 1.11 |
| ENSG00000196569 | LAMA2 | 0.10 | 0.27 | 0.33 | 4.70 |
| ENSG00000184564 | SLITRK6 | 0.00 | 0.19 | 0.65 | 6.88 |
| ENSG00000149599 | DUSP15 | 16.55 | 31.02 | 0.96 | 10.84 |
| ENSG00000106366 | SERPINE1 | 7.17 | 26.50 | 33.92 | 434.73 |
| ENSG00000118971 | CCND2 | 27.73 | 64.72 | 3.86 | 41.22 |
| ENSG00000154553 | PDLIM3 | 0.76 | 2.50 | 0.40 | 5.03 |
| ENSG00000146006 | LRRTM2 | 13.67 | 25.05 | 0.96 | 11.21 |
| ENSG00000065320 | NTN1 | 64.16 | 152.51 | 7.25 | 87.90 |
| ENSG00000095752 | IL11 | 0.74 | 4.30 | 0.50 | 6.41 |
| ENSG00000242242 | NECTIN3-AS1 | 0.46 | 6.07 | 0.46 | 6.51 |
| ENSG00000157680 | DGKI | 0.75 | 2.31 | 0.28 | 3.66 |
| ENSG00000197177 | ADGRA1 | 0.57 | 5.41 | 0.09 | 1.52 |
| ENSG00000206532 | AC117402.1 | 1.27 | 27.95 | 2.03 | 31.31 |
| ENSG00000174792 | C4orf26 | 1.26 | 8.55 | 0.40 | 8.71 |
| ENSG00000128610 | FEZF1 | 1.18 | 4.75 | 0.24 | 4.27 |
| ENSG00000148677 | ANKRD1 | 9.42 | 32.43 | 10.03 | 196.52 |
| ENSG00000115232 | ITGA4 | 1.24 | 6.47 | 0.98 | 22.54 |
| ENSG00000235597 | LINC01102 | 1.06 | 2.40 | 0.05 | 1.08 |
| ENSG00000182916 | TCEAL7 | 21.89 | 57.29 | 0.46 | 9.64 |
| ENSG00000171476 | HOPX | 4.28 | 19.08 | 0.70 | 14.79 |
| ENSG00000256894 | AC022509.3 | 1.18 | 2.74 | 0.04 | 1.90 |
| ENSG00000112333 | NR2E1 | 3.28 | 20.76 | 0.18 | 3.79 |
| ENSG00000128052 | KDR | 3.78 | 8.26 | 0.43 | 11.47 |
| ENSG00000102271 | KLHL4 | 3.78 | 13.38 | 0.13 | 3.12 |
| ENSG00000106689 | LHX2 | 8.83 | 29.38 | 1.00 | 28.25 |
| ENSG00000046604 | DSG2 | 0.16 | 0.58 | 0.24 | 6.73 |
| ENSG00000176697 | BDNF | 0.47 | 1.14 | 0.15 | 6.93 |
| ENSG00000178860 | MSC | 0.63 | 8.72 | 0.43 | 23.74 |
| ENSG00000163923 | RPL39L | 0.66 | 3.84 | 0.03 | 2.25 |
| ENSG00000132334 | PTPRE | 5.76 | 12.10 | 0.34 | 13.79 |
| ENSG00000123095 | BHLHE41 | 5.58 | 12.13 | 0.10 | 5.47 |
| ENSG00000116996 | ZP4 | 0.51 | 2.21 | 0.02 | 2.67 |
| ENSG00000205927 | OLIG2 | 0.06 | 0.76 | 0.02 | 3.38 |
| ENSG00000204969 | PCDHA2 | 2.52 | 4.76 | 0.01 | 1.08 |
| ENSG00000144481 | TRPM8 | 1.75 | 10.73 | 0.12 | 15.11 |
